# A novel approach for the synthesis of the cyclic lipopeptide globomycin†

**DOI:** 10.1039/d4md00685b

**Published:** 2024-10-21

**Authors:** Samantha J. Bann, Stephen A. Cochrane

**Affiliations:** a School of Chemistry and Chemical Engineering, Queen's University Belfast David Keir Building, Stranmillis Road Belfast BT9 5AG UK s.cochrane@qub.ac.uk

## Abstract

Cyclic lipopeptides (CLiPs) are a highly diverse class of secondary metabolites produced by bacteria and fungi. Examples of CLiPs have been found that possess potent antimicrobial activity against multidrug-resistant Gram-negative bacteria. Globomycin is a 19-membered CLiP that kills both Gram-positive and Gram-negative bacteria through inhibition of lipoprotein signal peptidase II (Lsp). It can only be obtained in small quantities from its *Streptomyces* producer strain, so there has been much interest in development of synthetic methods to access globomycin and analogues. Globomycin contains an N-terminal anti-α-methyl-β-hydroxy nonanoyl lipid tail, whose hydroxyl group forms an ester with the C-terminal carboxylate. Constructing the anti-arrangement between the α-methyl and β-hydroxy is synthetically challenging and previous globomycin syntheses are not compatible with diversification of the lipid tail after the stereocenters have been installed. Herein, we describe a new approach for the synthesis of globomycin that allows for facile lipid diversification. Using an anti-Evans Aldol condensation, a common intermediate is obtained that allows different “lipid swapping” through Grubbs-catalyzed cross-metathesis. Upon auxiliary cleavage, the resulting lipid can then be utilized in solid-phase peptide synthesis. Given the plethora of lipopeptides that contain β-hydroxy lipids, this method offers a convenient approach for convergent generation of lipopeptide analogues.

## Abstract 2

Lipid swapping: a new approach for the synthesis of globomycin that allows for facile lipid diversification.

## Graphical abstract

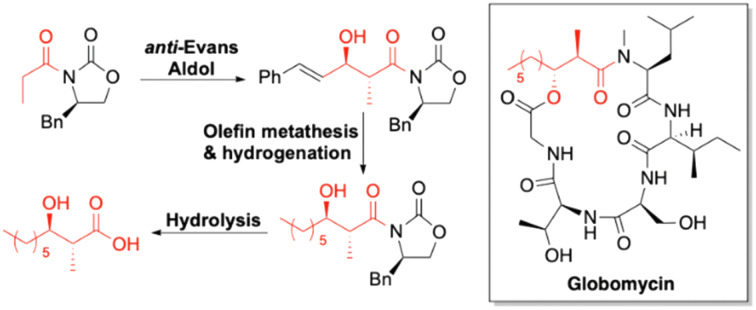

## Introduction

The ever-increasing threat of antimicrobial resistance (AMR) to the quality and sustainability of healthcare is well documented and it is predicted that by 2050, 10 million people will die annually due to AMR.^1–4^ Data from two Chinese hospitals in the midst of the COVID-19 pandemic found that secondary bacterial infections were associated with 50% of deaths, while only one survivor suffered from co-pathogenesis.^5^ These findings highlight the immense threat and opportunism of bacteria to cause both primary and co/secondary infections that increase both morbidity and mortality rates of viral infections.^6^ A recent global analysis of AMR, published in the Lancet in 2022, further highlights the perilous situation to which human healthcare could plummet, associating 4.95 million deaths to AMR in 2019 alone.^7^ The dangers of AMR are perpetuated by a growing innovation gap in the discovery of novel classes of antibiotics, only exacerbated by big pharma reducing or removing their antimicrobial R&D programs.^3,8,9^ Clinical overuse and misuse is another increasing concern as it is reported that between 2000 and 2010, the medicinal use of antibiotics rose by nearly 40%.^2,4^ Antimicrobial peptides (AMPs) are a diverse class of antimicrobial compounds. As of 2019, there were 34 AMPs in the preclinical stage of testing and 27 in clinical trials. Of those in clinical trials (phases I–III), nearly 80% were cationic in nature, highlighting the effectiveness with which peptides of this nature can target the negatively charged bacterial cell membrane.^10^

**Fig. 1:**
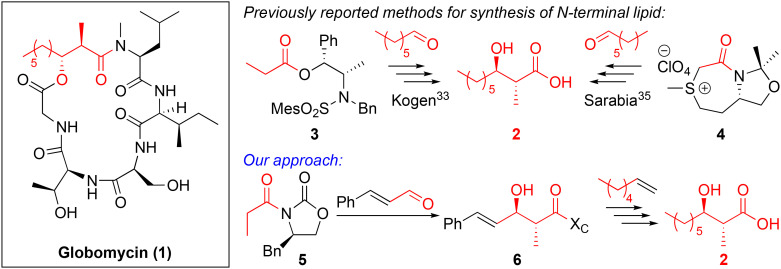
Structure of globomycin (1) (left), previously reported synthesis of the chiral lipid tail required for globomycin synthesis (top right) and our novel approach for preparation of this chiral lipid tail (bottom). X_C_ = chiral auxiliary.

Despite the interest in AMPs as drug candidates, the challenges associated with their industrial production and clinical application, such as cost,^11^ potential cytotoxicity and instability *in vivo*,^12^ have spawned only modest efforts in R&D.^13^ Making clinically viable AMPs can be problematic, owing to their rapid hydrolysis *in vivo*, toxicity issues and the cost of synthesis.^14^ Macrocyclic AMPs can benefit from increased permeability across the bacterial membrane and improved stability against enzymatic hydrolysis.^12,15^ Murepavadin was a promising peptidomimetic antibacterial candidate for treating Pneumonia; the first of its kind as an outer-membrane protein-targeting antibiotic. Unfortunately, its phase III clinical trial was terminated due to unexpected toxicity levels not initially found in healthy subjects.^16^ Cyclic lipopeptides (CLiPs) are a sub class of AMPs. They are non-ribosomally synthesized, contain a cyclic core, and are N-terminally acylated, offering diverse structures that can kill bacteria *via* several different avenues.^17,18^ Our current last-line-of-defence antibiotics for treating Gram-positive infections (daptomycin), and Gram-negative infections (polymyxin), are both CLiPs, highlighting that these compounds still have an important role to play in our fight against AMR. In recent years, there has been a resurgence in CLiP R&D, including the ornicidines,^19–22^ menaquinone-binding CLiPs^23–25^ and globomycin.^26,27^ Globomycin (1) (Fig. 1) was first discovered in 1978 and is produced by *Streptomyces* strains.^28^ Its antimicrobial activity against the Gram-negative strain *Pseudomonas aeruginosa* is due to its coordination and inhibition of lipoprotein signal peptidase II enzyme LspA.^29^ This enzyme is involved in the posttranslational processing of lipoproteins and is not found in humans, offering a selective drug target.^30^ Globomycin binds to the aspartic acid catalytic dyad in LspMrs (Lsp from MRSA) *via* hydrogen bonding from its Ser3 hydroxy group.^29^ This prevents cleavage of the signal peptide, which is responsible for anchoring the lipopeptide to the cytoplasmic membrane. LspA inhibition blocks the lipoprotein outer-membrane localization (Lol) pathway and results in accumulation of the lipoprotein precursor, prolipoprotein, causing cell death.^31^

**Scheme 1:**
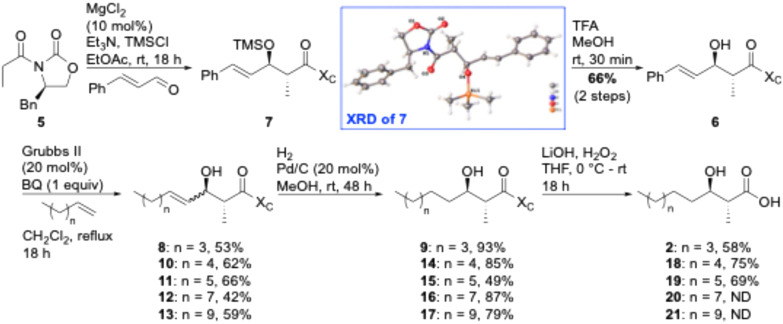
Novel synthesis of globomycin lipid tail. BQ = benzoquinone; ND = not determined; TFA = trifluoroacetic acid; TMS = trimethylsilyl; X_C_ = chiral auxiliary; XRD = X-ray diffraction.

Globomycin is a 19-membered cyclic depsipeptide that contains an N-terminal anti-α-methyl-β-hydroxy nonanoyl lipid tail. The β-hydroxy group is cyclized to the C-terminus through an ester bond. Globomycin is produced in small quantities (10 mg L^−1^ titre) by its producer strain,^28^ making total chemical synthesis the only viable option for larger quantities of material. Despite only being a pentapeptide, the total synthesis of globomycin is not trivial. Constructing the anti-α-methyl-β-hydroxy lipid tail is one of the main synthetic challenges. In the first reported total synthesis of globomycin,^32,33^ Kogen *et al.* utilized an anti-selective boron-mediated asymmetric aldol reaction (developed by Abiko and Masamune^34^) for preparation of carboxylic acid 2 (Fig. 1), which was a key intermediate in their solution-phase synthesis of globomycin. This approach provides acid 2 in excellent yields (93%, 94% diastereomeric excess) but chiral auxiliary 3 is very expensive (∼£500 per g, Sigma-Aldrich), or must be prepared in three steps from norepinephrine.^34^ In 2011, Sarabia *et al.* used a combination of solid-phase synthesis (peptide chain assembly) and solution-phase synthesis (cyclization step) to synthesize globomycin.^35^ For construction of acid 2, they employed an asymmetric epoxidation of heptanal using sulfur ylide 4 as a key step. From heptanal, acid 2 was obtained in 42.5% yield over four steps. However, sulfur ylide 4 must also be synthesized, requiring a four step-synthesis from l-methionine (46% overall) that includes two distillations and a recrystallization.^36^ We rationalized that use of Evans-type auxiliary 5 could allow for a cheaper, more-efficient synthesis of acid 2. Use of an α,β-unsaturated aldehyde in an anti-selective Aldol condensation would yield unsaturated lipid 6 that could then be diversified by “lipid swapping” through Grubbs-catalyzed cross metathesis reactions (Scheme 1). Inspired by previous total syntheses of brevicidine and laterocidine analogues,^19,20,37^ we aimed to perform the entirety of globomycin synthesis on resin.

## Results and discussion

We have previously utilized acetyl or propanoyl Evans-type auxiliaries in Aldol condensations to prepare the chiral lipids required for solid-phase peptide synthesis (SPPS) of tridecaptins^38,39^ and cerexins.^40^ For the synthesis of acid 2, propanoyl Evans auxiliary 5 was chosen as a cheap (∼£15 per g, Sigma-Aldrich) and readily available starting material. To allow lipid diversification after the α and β stereocentres have been set through an Aldol condensation, we required an alkene to be present in the resulting product. To achieve this, Evans previously reported procedure for magnesium-halide catalyzed anti-Aldol reactions of chiral *N*-acyloxazolidinones^41^ was used to perform an Aldol condensation between *trans*-cinnamaldehyde and Evans auxiliary 5 (Scheme 1), yielding enol 6 in 66% yield. The use of TMSCl in this reaction is required for silyation of the intermediate metal aldolate, turning over the metal center and improving diastereoselectivity. Serendipitously, during one synthesis of enol 6 the TMS deprotection step was omitted, resulting in isolation of TMS ether 7, which readily crystallized in column fraction tubes. An X-ray crystal structure of TMS ether 7 was obtained, unambiguously showing that the desired anti-selectivity had been achieved (see ESI†). Next, the use of Grubbs II-catalyzed cross-metathesis (CM), followed by alkene hydrogenation, was investigated to swap out the phenyl group with a pentene chain to give the anti-α-methyl-β-hydroxy nonanoyl lipid tail found in globomycin. We chose the Grubbs II catalyst as it's cheaper than Hoveyda–Grubbs variants. During optimization of the CM step, we found that enolization of the enol starting material 6 and product 8, to yield α-methyl-β-ketones, was a major competing side reaction. This side reaction was almost entirely eliminated by addition of 1 equiv. of benzoquinone. Alkene 8 was isolated as a mixture of *cis*- and *trans*-isomers, which were then reduced using H_2_ over a Pd/C catalyst to provide oxazolidinone 9 in 93% yield. In our initial efforts, we carried the crude CM reaction mixture through to hydrogenation and then performed column chromatography to isolate the desired product. However, reaction mixtures were complex and often resulted in co-elution of side-products with desired products. Therefore, we performed column chromatography after both the CM and hydrogenation steps. Finally, acid 2 was obtained by hydrolysis of oxazolidinone 9. Overall, this approach provides acid 2 in 19% yield over 4 steps. Before using acid 2 in the synthesis of globomycin, we investigated whether alkene 8 could be used as a common intermediate for the synthesis of chiral lipids of varying chain lengths. Gratifying, cross metathesis reactions between a range of linear terminal alkenes and alkene 8 proceeded smoothly to yield metathesis products 10–13, with the general trend being that product yields decreased as the chain length of the alkene increased. Hydrogenation of these alkenes yielded alkanes 14–17, mostly in excellent yields. Auxiliary cleavage proceeded smoothly to yield acids 18 and 19 but as chain length increased it became more difficult to separate the desired acid products from oxazolidinone by product.

**Scheme 2:**
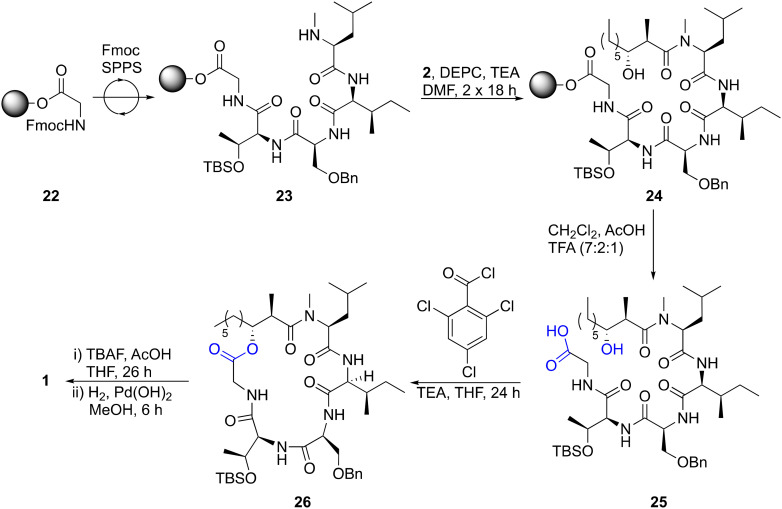
Synthesis of globomycin (1). Bn = benzyl; DEPC = diethyl cyanophosphonate; DMF = dimethylformamide; Fmoc = fluorenylmethyloxycarbonyl; TBAF = tetrabutylammonium fluoride; TBS = *tert*-butyldimethylsilyl; TEA = triethylamine; TFA = trifluoroacetic acid.

Next, we utilized acid 2 in the synthesis of globomycin (1) using the method previously reported by Sarabia *et al.* (Scheme 2).^35^ Fmoc-Gly-2CT (22) was first prepared with a loading of 0.5 mmol g^−1^, and Fmoc SPPS then used to synthesize resin-bound pentapeptide 23. Acylated peptide 24 was then prepared by treatment with two portions of acid 2, DEPC and TBS, Bn-protected pentapeptide 25 was cleaved from resin using 7 : 2 : 1 CH_2_Cl_2_ : AcOH : TFA and a Yamaguchi esterification used to prepare cyclic lipopeptide 26. Following sequential treatment with TBAF and H_2_/Pd(OH)_2_ to remove the TBS and Bn groups respectively, RP-HPLC purification was performed, providing pure globomycin (1) in 10% overall yield. HPLC co-injection with natural globomycin (Merck: G1424) showed only a single peak, providing confirmation that the synthetic product is identical to natural globomycin.

**Scheme 3:**
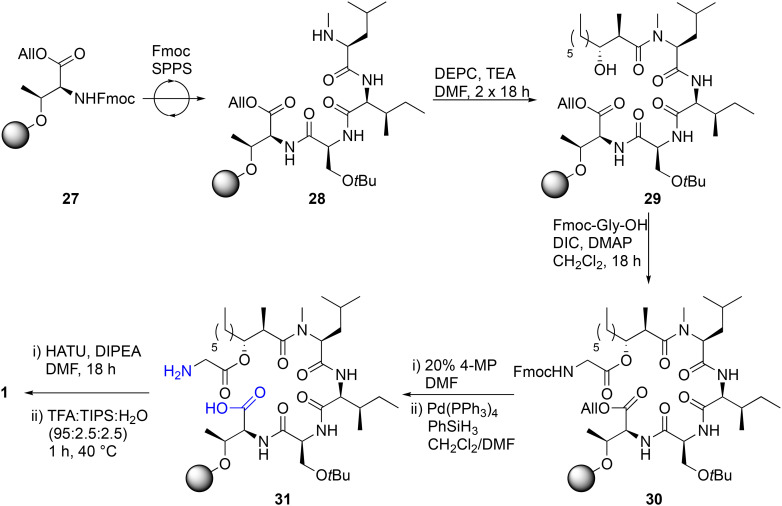
Unsuccessful total solid-phase synthesis of globomycin (1). All = allyl; Alloc = allyloxycarbonyl; DEPC = diethyl cyanophosphonate; DIC = diisopropylcarbodiimide; DIPEA = diisopropylethylamine; DMAP = 4-dimethylaminopyridine; DMF = dimethylformamide; Fmoc = fluorenylmethyloxycarbonyl; PyBOP = (1*H*-1,2,3-benzotriazol-1-yloxy)-tris(pyrrolidino)-phosphonium hexafluorophosphate; TEA = triethylamine; TIPS = triisopropylsilane.

We then embarked on an attempt to perform the total solid-phase synthesis of globomycin. To do this, we envisaged using a similar approach that we previously used to synthesize laterocidine.^19,20^ This first required immobilization of Fmoc-*allo*-Thr-OAllyl to an appropriate resin, ideally with a loading of 0.1–0.2 mmol g^−1^ so as to minimize dimerization during the on-resin cyclization step. A variety of resins were screened for their loading capacity of Fmoc-*allo*-Thr-OAllyl, including trityl (Trt) chloride, 2-chlorotrityl (2-CT) chloride and brominated Wang resin. However, none were able to anchor the desired residue *via* its side-chain hydroxy group in loadings high enough for fruitful SPPS. Eventually we identified that 4-methoxybenzhydryl (4-MeO-BH) bromide resin, previously utilized by Ficht *et al.* in the synthesis of thioester-containing peptides, allowed the desired loadings to be obtained (∼0.2 mmol g^−1^) (Scheme 3).^42^ Immobilized Fmoc-*allo*-Thr-OAllyl 27 was elongated into tetrapeptide 28 using Fmoc SPPS, wherein Fmoc deprotections were completed using 20% 4-methylpiperidine (4-MP) in DMF (2 × 1′, 1 × 5′) and couplings performed using HATU/DIPEA/DMF (1 h). To couple acid 2 on to the secondary amine of tetrapeptide 28, we utilized the method previously reported by Sarabia *et al.* (DEPC, TEA, DMF, 2 × 18 h), providing acylated tetrapeptide 29.^35^ Gly-1 was then installed through a Steglich esterification (DIC, DMAP, CH_2_Cl_2_, 18 h), and Fmoc and allyl protecting groups sequentially removed. A resin microcleavage was performed and HPLC analysis revealed a mixture of the desired linear precursor 31 (LC-MS, [M + H]^+^ calcd for C_36_H_67_N_5_O_10_H 730.5, found 730.3), as well as a side-product in which the glycine ester moiety had been hydrolyzed and allyl group deprotected (LC-MS, [M + H]^+^ calcd for C_34_H_64_N_4_O_9_H 673.5, found 673.3). We were unable to ascertain if this occurred due to incomplete Fmoc-Gly-OH coupling in the previous step, hydrolysis during subsequent basic Fmoc deprotections, or during the resin cleavage step. Therefore, it was decided to proceed with screening conditions for the final on-resin macrolactonisation step. Unfortunately, the final on-resin macrolactamization proved extremely challenging. Several conditions were screened, including BOP/DIPEA, DIC/HOBt/DMAP and HATU/DIPEA. Only when HATU was used as a coupling agent was any trace of desired product identified, and even then, the HPLC purification trace was very messy, and product was not present in sufficient quantities to isolate. In our previous synthesis of globomycin analogues in which the ester was replaced with amides or alkenes,^27^ we also found that on-resin cyclization steps were messy and low-yielding, suggesting these problems are amino acid sequence dependent. The results are in stark-contrast to on-resin cyclization of brevicidine or laterocidine, which proceed quantitatively with minimal side products.^20^

In summary, we have developed a new synthetic route to the antimicrobial peptide globomycin. Using an anti-Evans Aldol condensation, we obtained a common intermediate that allows for efficient lipid swapping *via* Grubbs-catalyzed cross-metathesis reactions. This approach addresses the synthetic challenges of constructing the anti-arrangement between the α-methyl and β-hydroxy groups and enables a streamlined synthesis of globomycin analogues with varied lipid tails. Although our investigations into a total solid-phase synthesis of globomycin did not surpass existing methods, they provided valuable insights that may prove useful in the future development of new methods to prepare globomycin. Future syntheses may also be able to incorporate chiral cinnamyl precursor 6 into globomycin, allowing late-stage lipid swapping. Overall, this approach offers a flexible and efficient framework for the synthesis of chiral lipid variants of antimicrobial peptides, which could be used in developing new antibiotic candidates.

## Data availability

The data supporting this article have been included as part of the ESI.† Crystallographic data for compound 7 was deposited at the CCDC on Sept 3rd 2024 (deposition number 2381556) and the URL for this is: https://www.ccdc.cam.ac.uk/structures/Search?Ccdcid=2381556&DatabaseToSearch=Published.

## Conflicts of interest

There are no conflicts of interest to declare.

## Supplementary Material

MD-OLF-D4MD00685B-s001
