# Supplementary material for: A novel approach for the synthesis of the cyclic lipopeptide globomycin
Source: RSC Med Chem. 2024 Oct 21;16(1):373–8. doi: 10.1039/d4md00685b (PMC11528322; doi:10.1039/d4md00685b)
Supplement: MD-016-D4MD00685B-s001 [file MD-016-D4MD00685B-s001.pdf]

Supplementary Information (SI) for RSC Medicinal Chemistry.
This journal is © The Royal Society of Chemistry 2024

Supporting Information for:

A Novel Approach for the Synthesis of the Cyclic Lipopeptide Globomycin

Samantha J Bann[1] and Stephen A. Cochrane[1]*

[1] School of Chemistry and Chemical Engineering, David Keir Building, Stranmillis Road, Queen's University Belfast, Belfast, BT9 5AG, UK.

*Address correspondence to the corresponding author:

Dr. Stephen Cochrane, Email: s.cochrane@qub.ac.uk

TABLE OF CONTENTS

S2: General information
S2: Compound characterization
S3: Peptide Purification
S4: Determination of peptide purity with analytical HPLC
S5: Chemical synthesis and compound characterization
S5: Enol 6
S7: TMS ether 7
S8: Alkene 8
S9: Alkane 9
S9: Acid 2
S10: Alkene 10
S11: Alkene 11
S11: Alkene 12
S12: Alkene 13
S13: Alkane 14
S13: Alkane 15
S14: Alkane 16
S14: Alkane 17
S15: Acid 18
S16: Acid 19
S16: Acid 20
S17: Acid 21
S17: Fmoc-Gly-2CT resin (22)
S18: Pentapeptide 23
S19: Acylated pentapeptide 24
S20: Synthesis of globomycin (1) using a solution-phase cyclization
S21: Fmoc-allo-Thr-OAll DiPh resin (27)
S22: Tetrapeptide 28
S23: Acylated tetrapeptide 29
S24: Unsuccessful synthesis of globomycin (1) by solid-phase cyclization
S25: Reagent combinations screened for solid-phase cyclisation
S26: HPLC comparison of synthetic globomycin with natural globomycin
S26: References
S27: Spectroscopic data of synthesized compounds

Experimental section

General information

All commercially available reagents and solvents were purchased from Merck Ltd., Thermo Fisher Scientific Ltd., Fluorochem Ltd., Alfa Aesar Ltd., Chem-Impex International Inc., VWR International or TCI UK Ltd., and used without further purification, unless otherwise stated. Deionized water was obtained from a Milli-Q water system (ThermoScientific, Davidson & Hardy Ltd., 18.2 MΩ x cm at 25 °C). Air/moisture-sensitive reactions were carried out in flame-dried glassware, in dried solvents under argon. Where stated, solvents were dried over activated 4 Å molecular sieves for 24 h and degassed under a stream of argon in flame-dried glassware. Reaction mixtures and pure samples were concentrated under vacuum using a Büchi Rotavapor® at a water bath temperature of 40 °C. Reactions were monitored via thin-layer chromatography (TLC) on aluminium plates (2.5 x 5 cm) pre-coated with silica gel (Merck 60 F_{254}), developed using potassium permanganate stain and heat, and visualized under shortwave UV light (254 nm). Silica gel (Fluorochem, 60 Å, 40 – 63u) and HPLC grade hexane (≥ 97% purity) were used for flash column chromatography purifications of smaller organic molecules. An automated CombiFlash NextGen 100 system was also used alongside Luknova SuperSep cartridges for purification of smaller organic molecules. Peptides were purified by preparative RP-HPLC using a water – acetonitrile (HPLC grade > 99.9%) gradient system, with an acidic (0.1% trifluoroacetic acid) buffer.

Compound characterization

Nuclear magnetic resonance (NMR) spectra were obtained using either a Bruker Ultrashield 400 MHz or Bruker Ascend 600 MHz magnet. Samples were dissolved in deuterated solvents and the respective residual solvent peaks referenced as follows: $CDCl_3$ δ 7.26, DMSO-d_6 δ 2.50 and CD_3OD δ 3.31. ^{13}C NMR chemical shifts were also referenced to $CDCl_3$ δ 77.16, DMSO-d_6 δ 39.52 and CD_3OD δ 49.00. $CDCl_3$ was stored over anhydrous potassium carbonate under argon, CD_3OD stored over anhydrous sodium sulfate under argon, and DMSO-d_6 was stored over activated 4Å molecular sieves. All chemical shifts were referenced to an internal tetramethylsilane standard and reported in ppm, while coupling

constants (J) are denoted in Hz. Peak multiplicities were described using the following abbreviations: s, singlet; d, doublet; t, triplet; q, quartet; m, multiplet; br. s, broad singlet; br. m, broad multiplet; *app.* t, apparent triplet. 2D NMR experiments were carried out for structure elucidation; homonuclear COrrelation SpectroscopY (COSY) was used to identify spin-systems with $^3J_{H\text{-}H}$ (vicinal) coupling, TOtal Correlation SpectroscopY (TOCSY) is helpful for protons within an isolated spin system, and Nuclear Overhauser Effect SpectroscopY (NOESY) produces cross peaks between protons with spatial proximity. These 2D experiments were particularly useful for peptide structure elucidation, as one can walk along the peptide backbone due to NOESY correlations between an amide proton of one amino acid and the Hα of an adjacent residue. 2D spectra involving ^{13}C nuclei were also utilised, namely Heteronuclear Single Quantum Correlation (HSQC) for the direct coupling between a proton bound to a ^{13}C nuclei, and Heteronuclear Multiple Bond Correlation (HMBC) of ^{13}C/^{1}H nuclei across two or three bonds. High resolution mass spectrometry (HR-MS) of all compounds were recorded and analysed by Analytical Services and Environmental Projects (ASEP) at Queen's University Belfast on a Waters LCT Premier ToF mass spectrometer, using the electrospray ionisation (ESI) technique. Liquid chromatography-mass spectrometry (LC-MS) analysis was conducted using a multifaceted system, run on a water-acetonitrile gradient, with an acidic (0.1% formic acid) buffer. Samples were first injected onto an Agilent Infinitylab poroshell 108 120 column (2.7 μm, 2.1 x 150 mm) using an Agilent 1260 HPLC system (including an Infinity II quaternary pump, vial sampler, integrated column compartment and variable wavelength detector). Compound ionisation was then detected using a single quadrupole mass spectrometer. Optical rotations, $[\alpha]^{20}_D$, were measured using a Perkin Elmer polarimeter (Model 341) with a 1 dm cell and 589 nm sodium lamp, at 20 °C. $CHCl_3$ or CH_2Cl_2 were used to calibrate a background reading.

Peptide Purification

Crude peptides were purified via Reversed-Phase High Performance Liquid Chromatography (RP-HPLC) using a Phenomenex Luna C18 column (5 μm, 250 x 21.2 mm) with a 2 mL sample loop. ThermoFisher Chromeleon 7.2 software was used to operate the Perkin Elmer HPLC system. Flow rate

was set at 10 mL/min and the UV/Vis detector measuring at 220 nm. Gradient elution was employed with 0.1 % trifluoracetic acid (TFA) in H_2O (A) and 0.1 % TFA in MeCN (B). Starting from 5% B and 95% A for 5 min, B was slowly ramped up to 40% over 25 min. B was then ramped up to 95% over 10 min and held for a further 5 min, before ramping down to 5% B over 0.1 min, and being held for the remaining 4.9 min.

All peaks containing the desired peptide were concentrated *in vacuo* and lyophilized on a Labyo Mini Freeze Drier (Frozen in Time). Electrospray ionisation high resolution mass spectrometry (ESI-HRMS) was then carried out on all purified peptides by the Analytical Services and Environmental Projects (ASEP) department at Queen's University Belfast. A Waters LCT Premier ToF mass spectrometer was used to obtain the relevant spectra.

Determination of peptide purity with analytical HPLC

Peptide purity was quantified by analytical HPLC, again using a ThermoFisher Chromeleon 7.2 operated Perkin Elmer HPLC system. A Phenomenex Luna C18 column (5 µm, 150 x 4.6 mm) with 200 µL sample loop was used with a flow rate of 2 mL/min and the UV/Vis detector measuring at 220 nm. The gradient began at 5% B and 95% A for 2 min, before ramping to 95% acetonitrile over 18 min. B was then decreased to 5% acetonitrile over 0.1 min and held for 3.9 min.

Chemical synthesis and compound characterization

Enol 6

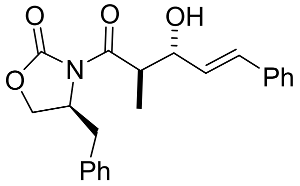

Compound **6** was synthesised according to a previously reported literature method[1]. To a flame-dried round-bottom flask was added added (*S*)-4-phenyl-3-propionyloxazolidin-2-one (2.30 g, 10 mmol, 1.0 equiv), anhydrous $MgCl_2$ (95 mg, 10 mol%, 1 mmol), dry triethylamine (2.8 mL, 20 mmol, 2.0 equiv), trans-cinnamaldehyde (1.8 mL, 14 mmol, 1.4 equiv), TMSCl (1.9 mL, 15 mmol, 1.5 equiv), and dry EtOAc (0.2M), creating a suspension. The reaction mixture was stirred under argon at rt for 18 h. The suspension was passed through a silica plug in diethyl ether, concentrated, and stirred in a methanolic deprotection solution containing 1-2 drops of TFA at rt. After 1 h, the crude mixture was concentrated and purified by automated flash chromatography using a 65 g Luknova SuperSep silica cartridge, across a 0 – 15% EtOAc/hexane gradient, ran at 40 mL/min over 40 min. Product-containing fractions were pooled and concentrated *in vacuo* to yield enol **6** as a white solid (2.45 g, 66%); *Rf* 0.17 (20% EtOAc/Hex); $[\alpha]^{20}_{D}$ = +31.5 ° (*c* = 1.7, CH_2Cl_2, lit. = +37.1 °); ^{1}H NMR (400 MHz; $CDCl_3$): δ 7.41 – 7.27 (br. m, 7H, aromatic), 7.26 – 7.18 (3H, br. m, -Ph), 6.68 (1H, d, *J* = 16 Hz, -HC=C<u>H</u>), 6.29 (1H, dd, *J* = 16, 7 Hz, -<u>H</u>C=CH), 4.74 – 4.68 (1H, m, -NC<u>H</u>), 4.47 (1H, app. t, *J* = 7 Hz, Hβ), 4.22 – 4.09 (3H, m, -OC$\underline{H_2}$ & Hα), 3.28 (1H, dd, *J* = 14, 3 Hz, -C$\underline{H}_2$Ph), 2.88 (1H, br. s, -OH), 2.71 (1H, dd, *J* = 14, 10 Hz, -C$\underline{H}_2$Ph), 1.26 (3H, d, *J* = 7 Hz, -αCH_3). ^{13}C NMR (151 MHz; $CDCl_3$): δ 176.4, 153.7, 136.5, 135.3, 132.4, 129.6, 129.1, 129.7, 128.8, 128.0, 127.5, 126.7, 75.8, 66.2, 55.6, 43.4, 37.9, 14.8. HRMS *m/z* $[2M+Na]^+$ calcd for $C_{44}H_{46}N_2O_8Na$ 753.3153, found 753.3141.

TMS ether 7

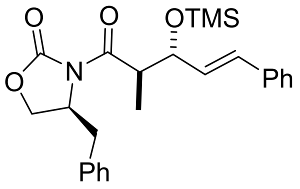

TMS ether **7** was synthesized according to a previously reported literature procedure[1].To a flame-dried round-bottom flask was added (*S*)-4-phenyl-3-propionyloxazolidin-2-one (2.79 g, 12 mmol, 1.0 equiv), anhydrous $MgCl_2$ (115 mg, 10 mol%), dry triethylamine (3.35 mL, 24 mmol, 2.0 equiv), trans-cinnamaldehyde (2.12 mL, 16.8 mmol, 1.4 equiv), and TMSCl (2.3 mL, 18 mmol, 1.5 equiv), and dry EtOAc (0.2M), creating a suspension. The reaction mixture was stirred under argon at rt for 18 h. The suspension was passed through a silica plug in diethyl ether and concentrated (Normally, TMS ether **7** is not purified but taken directly forward to the next deprotection step (see below). However, in this instance the TMS deprotection step was serendipitously omitted. We are reporting this because purified TMS ether **7** readily crystallized, allowing unambiguous stereochemical assignment). Crude product was purified by automated flash chromatography using a 40 g Luknova SuperSep silica cartridge, across a 0 – 15% EtOAc/hexane gradient, ran at 40 mL/min over 40 min. During purification, product spontaneously crystallized and crystals were filtered for subsequent XRD. *Rf* 0.74 (30% EtOAc/Hex); $[\alpha]^{20}_{D}$ = -24.8 ° (*c* = 0.28, $CHCl_3$); ^{1}H NMR (400 MHz; $CDCl_3$): δ 7.41 – 7.24 (10H, br. m, -Ph), 6.56 (1H, d, *J* = 16 Hz, -HC=C<u>H</u>), 6.17 (1H, dd, *J* = 16, 8 Hz, -<u>H</u>C=CH), 4.77 – 4.71 (1H, br. m, -NC<u>H</u>), 4.59 (1H, m, Hβ), 4.21 – 4.07 (3H, br. m, -OC$\underline{H_2}$ & Hα), 3.31 (1H, dd, *J* = 13, 3 Hz, -C$\underline{H_2}$Ph), 2.73 (1H, dd, *J* = 13, 10 Hz, -C$\underline{H_2}$Ph), 1.11 (3H, d, *J* = 7 Hz, -αCH_3), 0.11 (9H, s, -Si$\underline{(CH_3)_3}$); ^{13}C NMR (151 MHz; $CDCl_3$): δ 175.8, 153.3, 136.7, 135.6, 132.6, 130.4, 129.6, 129.1, 128.8, 128.0, 127.5, 126.7, 76.8 (obscured by $CHCl_3$), 65.9, 55.3, 44.6, 38.2, 14.1, 0.71. HRMS *m/z* $[M+Na]^+$ calcd for $C_{25}H_{31}NO_4SiNa$ 460.1921, found 460.1938.

*XRD analysis of TMS-protected anti-aldol **6**.* Crystals of **6** were analysed by Laura J McCormick McPherson (UK National Crystallography Service), from whom results, and experimental data were also obtained. Single colourless rod-shaped-shaped crystals of 2023NCS0597_1a were supplied from which a crystal was chosen for analysis. A suitable crystal 0.360×0.040×0.030 mm^3 was selected and mounted

on a MITIGEN holder in oil on a Rigaku 007HF equipped with ArcSec VHF Varimax confocal mirrors and a UG2 goniometer and HyPix-Arc 100 detector. The crystal was kept at a steady *T* = 100(2) K during data collection. The structure was solved with the ShelXT 2018/2 (Sheldrick, 2018) structure solution program using the using dual methods solution method and by using Olex2 1.5 (Dolomanov et al., 2009) as the graphical interface. The model was refined with version 2018/3 of ShelXL 2018/3 (Sheldrick, 2015) using full matrix least squares minimisation on $\boldsymbol{F}^2$ minimisation.

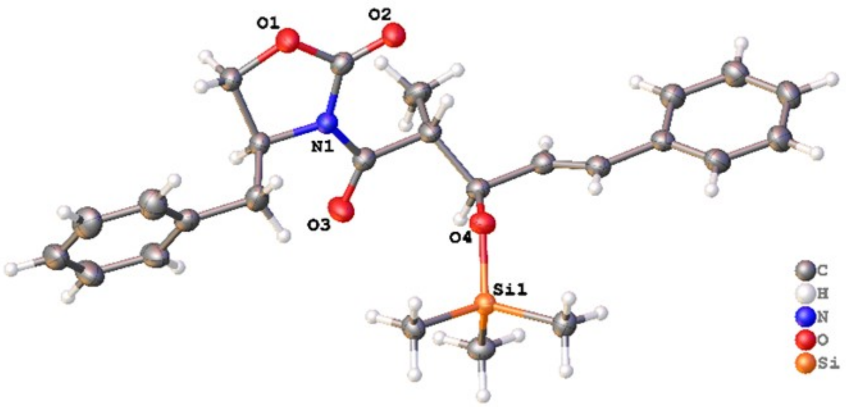

Figure X. Crystal Data for $C_{25}H_{31}NO_4Si$, M_r = 437.60, monoclinic, $P2_1$ (No. 4), a = 11.65355(9) Å, b = 5.86208(4) Å, c = 18.35384(16) Å, *b* = 108.0587(9)˚, *a* = *g* = 90˚, *V* = 1192.061(17) Å^3, *T* = 100(2) K, *Z* = 2, *Z'* = 1, *m*(Cu K$_a$) = 1.112 mm^{-1}, 213681 reflections measured, 4481 unique (R_{int} = 0.0675) which were used in all calculations. The final wR_2 was 0.0662 (all data) and R_1 was 0.0241 (I≥2 *s*(I)).

Alkene 8

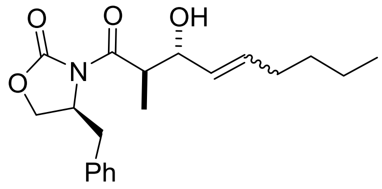

To a flame-dried round-bottom flask containing dry DCM (30 mL), was added enol **6** (1.77 g, 4.80 mmol, 1.0 equiv), hexene (3 mL, 24.2 mmol, 5 equiv), 1,4-benzoquinone (530 mg, 4.84 mmol, 1.0 equiv), and Grubbs II catalyst (20 mol%, 835 mg). The reaction mixture was refluxed for 18 h before being cooled and DMSO (3.45 mL, 50 equiv. relative to catalyst loading) added. The mixture was stirred at rt for a further 18 h and then concentrated to remove residual hexene. The crude mixture was then redissolved in DCM (50 mL), washed with warm H_2O (30 °C) (3x 50 mL) and the combined aqueous washings back extracted with DCM (1x 50 mL). The combined organic layers were then washed with brine and dried over anhydrous Na_2SO_4. The crude reaction mixture was purified via automated flash chromatography, using a 40 g Luknova SuperSep silica cartridge, across a 5 – 20% EtOAc/hexane gradient, ran at 40 mL/min over 60 min. The product-containing fractions were pooled and concentrated *in vacuo* to obtain alkene **8** as a blue oil (880 mg, 53%), *Rf* 0.47 (30% EtOAc/Hex); $[\alpha]^{20}_D$ = +14.2 ° (*c* = 0.75, $CHCl_3$); ^1H NMR (400 MHz; $CDCl_3$): δ 7.36 – 7.23 (5H, m, -Ph), 5.80 – 5.72 (1H, m, -HC=C$\underline{H}$), 5.51 (1H, ddt, *J* =15, 7, 1 Hz, -$\underline{H}$C=CH), 4.73 – 4.67 (1H, m, -NC$\underline{H}$), 4.25 – 4.15 (3H, m, -OC$\underline{H_2}$ and Hβ), 3.98 – 3.91 (1H, m, Hα), 3.30 (1H, dd, *J* = 13, 3 Hz, -C$\underline{H}_2$Ph), 2.78 (1H, dd, *J* = 14, 9 Hz, -C$\underline{H}_2$Ph), 2.52 (1H, d, *J* = 7 Hz, -OH), 2.07 (2H, q, *J* = 7 Hz, -HC=CHC$\underline{H_2}$), 1.39 – 1.31 (4H, br. m, -HC=CHCH$_2$C$\underline{H_2}$$\underline{CH_2}$), 1.17 (3H, d, *J* = 7 Hz, -αCH_3), 0.89 (3H, t, *J* = 7 Hz, -CH_3). ^{13}C NMR (101 MHz; CDCl3): δ 176.5, 153.6, 135.4, 134.6, 130.2, 129.6, 129.0, 127.4, 76.0, 66.1, 55.6, 43.5, 37.9, 32.0, 31.3, 22.3, 14.6, 14.0. HRMS *m/z* $[2M+Na]^+$ calcd for $C_{40}H_{54}N_2O_8Na$ 713.3779, found 713.3765.

Alkane 9

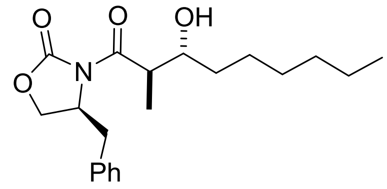

Alkene **8** (880 mg, 2.55 mmol, 1.0 equiv) was dissolved in dry MeOH (50 mL) and added to a flame-dried round-bottom flask. The system was flushed with argon before adding 10% wt Pd/C (20 mol%, 545 mg). Once added, the system was saturated with H_2 gas for 10 min and then sealed and stirred under an atmosphere of H_2 for 48 h. Upon completion, the system was flushed with argon, Pd/C was removed by passing the crude mixture through a celite plug, and the filtrate was concentrated to yield the alkane **9** as a colourless oil, requiring no further purification (826 mg, 93%); *Rf* 0.45 (30% EtOAc/Hex); $[\alpha]^{20}_D$ = +8.3 ° (*c* = 1.1, $CHCl_3$); ^1H NMR (400 MHz; $CDCl_3$): δ 7.35 – 7.23 (5H, m, -Ph), 4.69 (1H, ddt, *J* = 10, 7, 3 Hz, -NC<u>H</u>), 4.22 – 4.15 (2H, m, -OC<u>H₂</u>), 3.89 (1H, apparent quintet, *J* = 7 Hz, Hα), 3.76 – 3.69 (1H, br. m, Hβ), 3.32 (1H, dd, *J* = 13, 3 Hz, -C<u>H₂</u>Ph), 2.77 (1H, dd, *J* = 14, 10 Hz, -C<u>H₂</u>Ph), 2.56 – 2.53 (1H, br. m, -OH), 1.63 – 1.26 (10H, br. m, -(C<u>H₂</u>)₅CH₃), 1.22 (3H, d, *J* = 7 Hz, -αCH₃), 0.88 (3H, t, *J* = 7 Hz, -CH₃). ^{13}C NMR (151 MHz; $CDCl_3$): δ 177.1, 153.7, 135.4, 129.6, 129.1, 127.5, 74.9, 66.2, 55.7, 43.4, 38.0, 35.2, 31.9, 29.4, 25.6, 22.8, 14.8, 14.2. HRMS *m/z* $[2M+Na]^+$ calcd for $C_{40}H_{58}N_2O_8Na$ 717.4092, found 717.4106.

Acid 2

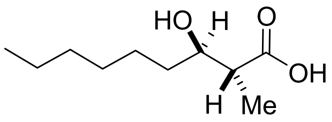

Auxiliary-bound intermediate **9** (283 mg, 0.74 mmol, 1.0 equiv) was dissolved in THF (0.1 M; 7.5 mL) and cooled to 0 °C. 30% aq. H_2O_2 solution (455 μL, 4.4 mmol, 6 equiv) and LiOH•H_2O (62 mg, 1.48 mmol, 2 equiv) were added and the reaction stirred at rt overnight. The crude mixture was quenched with sat. aq. Na_2SO_3 (1 mL) at 0 °C and concentrated *in vacuo*, before being resuspended in H_2O and washed with DCM (2 x 10 mL). The resulting aqueous layer was acidified to ~pH 2 using 1M HCl and extracted with DCM (3 x 10 mL), and the combined organic layers washed with brine and dried over

anhydrous Na_2SO_4. The crude filtrate was purified by automated flash chromatography using a 12 g Luknova SuperSep silica cartridge, across a 0 – 8% MeOH/DCM + 0.1% AcOH gradient, ran at 20 mL/min over 40 min. Product fractions were pooled and concentrated *in vacuo* to yield natural lipid **13** as a colourless oil (81 mg, 58%); *Rf* 0.25 (5% MeOH/DCM); $[\alpha]^{20}_D$ = +1.9 ° (*c* = 0.53, $CHCl_3$, lit. = +4.8 °); ^1H NMR (400 MHz; $CDCl_3$): δ 3.74 – 3.69 (1H, br. m, Hβ), 2.62 – 2.55 (1H, m, Hα), 1.56 – 1.41 (3H, br. m, $-(C\underline{H_2})_5CH_3$), 1.37 – 1.23 (10H, br. m, $-(C\underline{H_2})_5CH_3$ & $-\alpha CH_3$), 0.92 – 0.89 (3H, m, $-CH_3$). ^{13}C NMR (151 MHz; $CDCl_3$): δ 181.0, 73.5, 45.4, 34.8, 31.9, 29.4, 25.6, 22.8, 14.4, 14.1. HRMS *m/z* $[M-H]^-$ calcd for $C_{10}H_{19}O_3$ 187.1335, found 187.1324.

Alkene 10

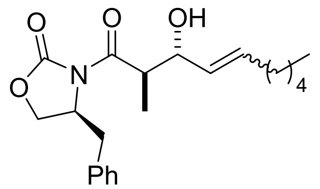

Alkene **10** was synthesized from enol **6** using the conditions outlined for the synthesis of alkene **8**. Enol **6** was cross-coupled with 1-heptene (5 equiv) and isolated using purifications conditions for cross-metathesis product **8**, yielding the product as a blue oil (60 mg, 62%); *Rf* 0.51 (30% EtOAc/Hex); $[\alpha]^{20}_D$ = +24.9 ° (*c* = 0.51, $CHCl_3$); ^1H NMR (400 MHz; $CDCl_3$) *δ* 7.34 – 7.27 (3H, m, -Ph), 7.24 – 7.22 (2H, m, -Ph), 5.79 – 5.72 (1H, m, $-HC=C\underline{H}$), 5.51 (1H, ddt, *J* = 15.3, 7.3, 1.4 Hz, $-\underline{H}C=CH$), 4.69 (1H, ddt, *J* = 9.4, 7.3, 3.3 Hz, $-NC\underline{H}$), 4.24 – 4.14 (3H, m, $-OC\underline{H_2}$ and Hβ), 3.97 – 3.90 (1H, m, Hα), 3.29 (1H, dd, *J* = 13.5, 3.4 Hz, $-C\underline{H_2}Ph$), 2.77 (1H, dd, *J* = 13.5, 9.4 Hz, $-C\underline{H_2}Ph$), 2.05 (2H, q, *J* = 7.1 Hz, $-HC=CHC\underline{H_2}$), 1.38 (2H, dt, *J* = 14.5, 7.3 Hz, $-HC=CHCH_2C\underline{H_2}$), 1.29 – 1.25 (4H, m, $-\underline{(CH_2)_2}CH_3$), 1.16 (3H, d, *J* = 6.9 Hz, $-\alpha CH_3$), 0.87 (3H, t, *J* = 6.9 Hz, $-CH_3$). ^{13}C NMR (101 MHz; CDCl3): δ 176.5, 153.6, 135.4, 134.6, 130.2, 129.6, 129.0, 127.4, 76.0, 66.1, 55.6, 43.4, 37.9, 32.3, 31.5, 28.8, 22.6, 14.6, 14.1. HRMS *m/z* $[M+Na]^+$ calcd for $C_{21}H_{29}NO_4Na$ 382.1995, found 382.1978.

Alkene 11

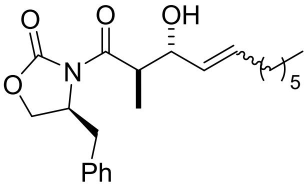

Alkene **11** was synthesized according to the conditions outlined for cross-metathesis product **8**. Enol **6** was cross-coupled with 1-octene (5 equiv) and isolated using purifications conditions for cross-metathesis product **8**. Product-containing fractions were pooled and concentrated to yield a blue oil (67 mg, 66%); *Rf* 0.51 (30% EtOAc/Hex); $[\alpha]^{20}_{D}$ = +25.9 ° (*c* = 0.49, $CHCl_3$); ^1H NMR (400 MHz; $CDCl_3$) *δ* 7.35 – 7.27 (3H, m, -Ph), 7.25 – 7.23 (2H, m, -Ph), 5.79 – 5.72 (1H, m, -HC=C<u>H</u>), 5.54 – 5.48 (1H, dt, *J* = 15.3, 7.3, 1.4 Hz, -<u>H</u>C=CH), 4.69 (1H, ddt, *J* = 9.4, 7.3, 3.3 Hz, -NC<u>H</u>), 4.24 – 4.14 (3H, br. m, -OC$\underline{H_2}$ and Hβ), 3.98 – 3.89 (1H, m, Hα), 3.30 (1H, dd, *J* = 13.5, 3.4 Hz, -C$\underline{H_2}$Ph), 2.77 (1H, dd, *J* = 13.5, 9.4 Hz, -C$\underline{H_2}$Ph), 2.57 – 2.55 (1H, m, -OH), 2.06 (2H, q, *J* = 7.0 Hz, -HC=CHC$\underline{H_2}$), 1.40 – 1.34 (2H, m, -HC=CHCH$_2$C$\underline{H_2}$), 1.30 – 1.25 (6H, m, -$\underline{(CH_2)_3}$CH$_3$), 1.17 (3H, d, *J* = 6.9 Hz, -αCH_3), 0.87 (3H, t, *J* = 6.8 Hz, -CH_3). ^{13}C NMR (101 MHz; CDCl3): δ 176.6, 153.6, 135.4, 134.7, 130.2, 129.6, 129.1, 127.4, 76.0, 66.1, 55.6, 43.5, 37.9, 32.4, 31.8, 29.2, 29.0, 22.7, 14.6, 14.2. HRMS *m/z* $[M+NH_4]^+$ calcd for $C_{22}H_{31}NO_4Na$ 391.2597, found 391.2570.

Alkane 12

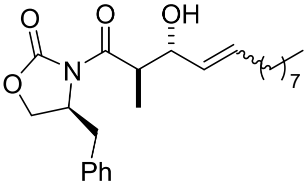

Compound **12** was synthesized according to the conditions outlined for cross-metathesis product **8**. Enol **6** was cross-coupled with 1-decene (5 equiv) and isolated using purifications conditions described for cross-metathesis product **8**. Product-containing fractions were pooled and concentrated to yield a blue oil (120 mg, 42%); *Rf* 0.73 (30% EtOAc/Hex); $[\alpha]^{20}_{D}$ = +22.8 ° (*c* = 0.55, $CHCl_3$); ^1H NMR (400 MHz; $CDCl_3$) *δ* 7.35 – 7.27 (3H, m, -Ph), 7.25 – 7.23 (2H, m, -Ph), 5.80 – 5.72 (1H, m, -HC=C<u>H</u>), 5.54 – 5.48 (1H, m, -<u>H</u>C=CH), 4.72 – 4.67 (1H, m, -NC<u>H</u>), 4.24 – 4.14 (3H, br. m, -OC$\underline{H_2}$ and Hβ), 3.94 (1H,

m, Hα), 3.30 (1H, dd, J = 13.5, 3.3 Hz, -C$\underline{H_2}$Ph), 2.77 (1H, dd, J = 13.5, 9.5 Hz, -C$\underline{H_2}$Ph), 2.55 (1H, d, J = 4.4 Hz, -OH), 2.05 (2H, q, J = 7.1 Hz, -HC=CHC$\underline{H_2}$), 1.41 – 1.34 (2H, br. m, -HC=CHCH$_2$C$\underline{H_2}$), 1.30 - 1.24 (15H, m, -$\underline{(CH_2)_5}$CH$_3$, plus grease), 1.17 (3H, d, J = 6.9 Hz, -αCH$_3$), 0.88 (6H, m, -CH$_3$ plus grease). ^{13}C NMR (101 MHz; CDCl3): δ 176.6, 153.6, 135.4, 134.7, 130.2, 129.6, 129.1, 127.4, 76.0, 66.1, 55.6, 43.5, 38.0, 32.4, 32.0, 31.7, 29.6, 29.4, 29.2, 22.8, 14.7, 14.2. HRMS m/z [M+Na]$^+$ calcd for $C_{24}H_{35}NO_4Na$ 424.2465, found 424.2438.

Alkene 13

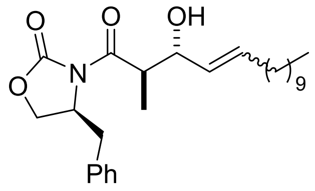

Compound **13** was synthesized according to the conditions outlined for cross-metathesis product **8**. Enol **6** was cross-coupled with 1-dodecene (5 equiv) and isolated using purifications conditions described for cross-metathesis product **8**. Product-containing fractions were pooled and concentrated to yield a blue oil (169 mg, 59%); Rf 0.73 (30% EtOAc/Hex); $[\alpha]^{20}_D$ = +19.3 ° (c = 0.46, CHCl$_3$); ^{1}H NMR (400 MHz; CDCl$_3$) δ 7.35 – 7.26 (3H, m, -Ph), 7.26 – 7.23 (2H, m, -Ph), 5.79 – 5.72(1H, m, -HC=C$\underline{H}$), 5.54 – 5.48 (1H, m, -$\underline{H}$C=CH), 4.72 – 4.66 (1H, m, -NC$\underline{H}$), 4.25 – 4.14 (3H, br. m, -OC$\underline{H_2}$ and Hβ), 3.98 – 3.91 (1H, m, Hα), 3.30 (1H, dd, J = 13.5, 3.4 Hz, -C$\underline{H_2}$Ph), 2.77 (1H, dd, J = 13.5, 9.4 Hz, -C$\underline{H_2}$Ph), 2.58 (1H, d, J = 6.8 Hz, -OH), 2.05 (2H, q, J = 7.1 Hz, -HC=CHC$\underline{H_2}$), 1.39 - 1.34 (2H, m, -C=CHCH$_2$C$\underline{H_2}$), 1.31 - 1.21 (14H, br. m, -$\underline{(CH_2)_7}$CH$_3$), 1.17 (3H, d, J = 6.9 Hz, -αCH$_3$), 0.87 (3H, t, J = 6.9 Hz, -CH$_3$). ^{13}C NMR (101 MHz; CDCl3): δ 176.5, 153.6, 135.4, 134.7, 130.2, 129.6, 129.0, 127.4, 76.0, 66.1, 55.6, 43.4, 37.9, 32.4, 32.0, 29.73, 29.71, 29.6, 29.4, 29.3, 29.2, 22.8, 14.6, 14.2. HRMS m/z [M+Na]$^+$ calcd for $C_{26}H_{39}NO_4Na$ 452.2778, found 452.2758.

Alkane 14

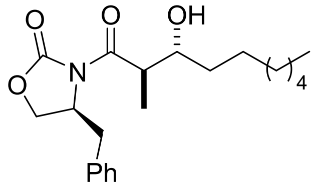

Compound **14** was synthesized according to the conditions outlined for hydrogenation product **9**. Alkene **10** was reduced under hydrogen and isolated as a colourless oil (22 mg, 85%); *Rf* 0.34 (20% EtOAc/Hex); ^{1}H NMR (400 MHz; $CDCl_3$) *δ* ^{1}H NMR (400 MHz; $CDCl_3$) *δ* 7.35 – 7.28 (3H, m, -Ph), 7.24 – 7.22 (2H, m, -Ph), 4.71 – 4.66 (1H, m, -NC<u>H</u>), 4.21 – 4.14 (2H, m, -OC$\underline{H_2}$), 3.89 (1H, quintet, *J* = 6.9 Hz, Hα), 3.74 – 3.73 (1H, m, Hβ), 3.32 (1H, dd, *J* = 13.5, 3.3 Hz, -C$\underline{H_2}$Ph), 2.77 (1H, dd, *J* = 13.5, 9.6 Hz, -C$\underline{H_2}$Ph), 2.56 (1H, br. s, -O<u>H</u>), 1.64 – 1.40 (4H, br. m, aliphatic protons), 1.33 – 1.25 (8H, br. m, aliphatic protons), 1.21 (3H, d, *J* = 6.9 Hz, -αCH_3), 0.88 (3H, t, *J* = 6.8 Hz, -CH_3). ^{13}C NMR (101 MHz; $CDCl_3$): *δ* 177.0, 153.7, 135.4, 129.6, 129.1, 127.5, 74.8, 66.2, 55.7, 43.4, 38.0, 35.1, 31.9, 29.7, 29.4, 25.6, 22.8, 14.8, 14.2. HRMS *m/z* $[M+CH_3OH+Na]^+$ calcd for $C_{22}H_{35}NO_5Na$ 416.2414, found 416.2409.

Alkane 15

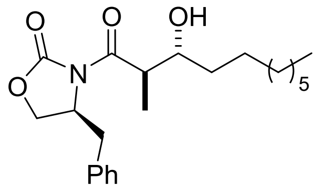

Compound **15** was synthesized according to the conditions outlined for hydrogenation product **9**. Alkene **11** was reduced under hydrogen and isolated as a colourless oil (33 mg, 49%); *Rf* 0.34 (20% EtOAc/Hex); ^{1}H NMR (400 MHz; $CDCl_3$) *δ* ^{1}H NMR (400 MHz; $CDCl_3$) δ 7.36 – 7.27 (3H, m, -Ph), 7.25 – 7.23 (2H, m, -Ph), 4.69 (1H, ddt, *J* = 9.8, 6.7, 3.2 Hz, -NC<u>H</u>), 4.22 – 4.15 (2H, m, -OC$\underline{H_2}$), 3.89 (1H, quintet, *J* = 6.9 Hz, Hα), 3.74 – 3.71 (1H, m, Hβ), 3.33 (1H, dd, *J* = 13.5, 3.4 Hz, -C$\underline{H_2}$Ph), 2.77 (1H, dd, *J* = 13.5, 9.6 Hz, -C$\underline{H_2}$Ph), 2.54 (1H, d, J = 7.2 Hz, -O<u>H</u>), 1.64 – 1.41 (4H, br. m, aliphatic protons), 1.33 – 1.24 (10H, m, aliphatic protons), 1.22 (3H, d, *J* = 6.9 Hz, -αCH), 0.88 (3H, t, *J* = 6.9 Hz, -CH_3). ^{13}C NMR (101 MHz; $CDCl_3$): δ 177.1, 153.7, 135.4, 129.6, 129.1, 127.5, 74.9, 66.2, 55.7, 43.4, 38.0, 35.2,

32.0, 29.73, 29.70, 29.4, 25.6, 22.8, 14.8, 14.2. HRMS *m/z* $[M+CH_3OH+Na]^+$ calcd for $C_{22}H_{33}NO_4Na$ 398.2308, found 398.2352.

Alkane 16

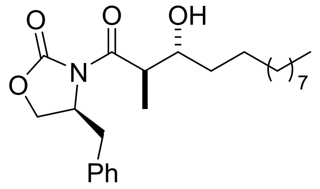

Compound **16** was synthesized according to the conditions outlined for hydrogenation product **9**. Alkene **12** was reduced under hydrogen and isolated as a colourless oil (94 mg, 87%); *Rf* 0.24 (20% EtOAc/Hex); ^{1}H NMR (600 MHz; $CDCl_3$) *δ* 7.35 – 7.32 (2H, m, -Ph), 7.30 – 7.27 (1H, m, -Ph), 7.24 (2H, d, *J* = 7.6 Hz, -Ph), 4.70 – 4.67 (1H, m, -NC$\underline{H}$), 4.21 – 4.16 (2H, m, -OC$\underline{H_2}$), 3.89 (1H, quintet, *J* = 6.8 Hz, Hα), 3.75 – 3.70 (1H, m, Hβ), 3.33 (1H, dd, *J* = 13.7, 3.4 Hz, -C$\underline{H_2}$Ph), 2.77 (1H, dd, *J* = 13.6, 9.6 Hz, -C$\underline{H_2}$Ph), 2.52 (1H, d, *J* = 8.4 Hz, -O$\underline{H}$), 1.66 – 1.47 (6H, br. m, aliphatic protons & solvent impurities), 1.33 – 1.27 (14H, br. m, aliphatic protons), 1.22 (3H, d, *J* = 6.9 Hz, -αCH_3), 0.88 (3H, t, *J* = 7.0 Hz, -CH_3). ^{13}C NMR (151 MHz; CDCl3): δ 177.1, 153.7, 135.4, 129.6, 129.1, 127.5, 74.9, 66.2, 55.7, 43.4, 38.0, 35.2, 32.1, 29.78, 29.77, 29.76, 29.74, 29.5, 25.6, 22.8, 14.8, 14.3. HRMS *m/z* $[M+CH_3OH+H]^+$ calcd for $C_{25}H_{42}NO_5$ 436.3064, found 436.3069.

Alkane 17

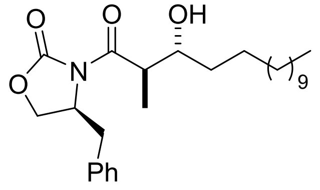

Alkane **17** was synthesized according to the conditions outlined for hydrogenation product **9**. Alkene **13** was reduced under hydrogen and isolated as a colourless oil, exhibiting a sickly-sweet odour (129 mg, 79%); *Rf* 0.24 (20% EtOAc/Hex); ^{1}H NMR (600 MHz; $CDCl_3$) *δ* 7.33 (2H, t, *J* = 7.4 Hz, -Ph), 7.28 (1H, d, *J* = 7.2 Hz, -Ph), 7.24 (2H, d, *J* = 7.3 Hz, -Ph), 4.69 (1H, ddt, *J* = 9.9, 6.8, 3.2 Hz, -NC$\underline{H}$), 4.21 –4.16

(2H, m, -OC$\underline{H_2}$), 3.89 (1H, quintet, *J* = 6.9 Hz, Hα), 3.76 – 3.70 (1H, m, Hβ), 3.33 (1H, dd, *J* = 13.4, 3.3 Hz, -C$\underline{H_2}$Ph), 2.77 (1H, dd, *J* = 13.5, 9.6 Hz, -C$\underline{H_2}$Ph), 2.52 (1H, d, *J* = 8.7 Hz, -OH), 1.61 – 1.56 (2H, br. m, aliphatic protons), 1.29 – 1.26 (20H, br. m, aliphatic protons), 1.22 (3H, d, *J* = 6.9 Hz, -αCH_3), 0.88 (3H, t, *J* = 7.0 Hz, -CH_3). ^{13}C NMR (151 MHz; CDCl3): δ 177.1, 153.7, 135.4, 129.6, 129.1, 127.5, 74.9, 66.2, 55.7, 43.4, 38.0, 35.2, 32.1, 29.83, 29.81, 29.80, 29.78, 29.76, 29.74, 29.5, 25.6, 22.8, 14.8, 14.3. HRMS *m/z* [M+CH_3OH+Na]$^+$ calcd for $C_{25}H_{41}NO_5Na$ 486.3196, found 486.3274.

Acid 18

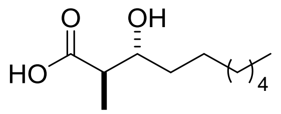

Compound **18** was synthesized according to the conditions outlined for acid **2**. Hydrogenation product **14** (16.4 mg, 45 μmol, 1 equiv.) was dissolved in THF (1 mL) and cooled to 0 °C. 30% aq. H_2O_2 solution (28 μL, 0.27 mmol, 6 equiv) and LiOH•H_2O (4 mg, 0.09 mmol, 2 equiv) were added and the reaction stirred at rt overnight. The crude mixture was quenched with sat. aq. Na_2SO_3 (100 μL) at 0 °C and concentrated *in vacuo*, before being resuspended in H_2O and washed with DCM (2 x 3 mL). The resulting aqueous layer was acidified to ~pH 2 using 1M HCl and extracted with DCM (3 x 3 mL), and the combined organic layers washed with brine and dried over anhydrous Na_2SO_4. The filtrate was concentrated *in vacuo* to obtain acid **18** as a colourless oil (6.9 mg, 75%); *Rf* 0.29 (4% MeOH/DCM + 0.1% AcOH; ^{1}H NMR (600 MHz; MeOD) *δ* 3.72 - 3.69 (1H, m, Hβ), 2.48 (1H, quintet, *J* = 7.0 Hz, Hα), 1.54—1.29 (13H, m, aliphatic & solvent), 1.12 (3H, d, *J* = 7.1 Hz, -αCH_3), 0.90 (3H, t, *J* = 6.9 Hz, -CH_3). ^{13}C NMR (151 MHz; MeOD): δ 179.2, 74.0, 47.4, 34.8, 33.0, 30.7, 30.4, 26.7, 23.7, 14.4, 13.6. HRMS *m/z* [M+Na]$^+$ calcd for $C_{11}H_{22}O_3Na$ 225.1468, found 225.1465.

Acid 19

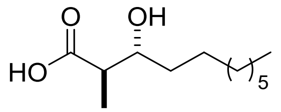

Compound **19** was synthesized according to the conditions outlined for acid **2**. Hydrogenation product **15** (11.8 mg, 31 μmol, 1 equiv.) was dissolved in THF (1 mL) and cooled to 0 °C. 30% aq. H_2O_2 solution (20 μL, 0.19 mmol, 6 equiv) and LiOH•H_2O (2.8 mg, 0.06 mmol, 2 equiv) were added and the reaction stirred at rt overnight. The crude mixture was quenched with sat. aq. Na_2SO_3 (50 μL) at 0 °C and concentrated *in vacuo*, before being resuspended in H_2O and washed with DCM (2 x 3 mL). The resulting aqueous layer was acidified to ~pH2 using 1M HCl and extracted with DCM (3 x 3 mL), and the combined organic layers washed with brine and dried over anhydrous Na_2SO_4. The filtrate was concentrated *in vacuo* to obtain acid **19** as a colourless oil (4.7 mg, 69%); *Rf* 0.29 (4% MeOH/DCM + 0.1% AcOH); ^{1}H NMR (600 MHz; MeOD) *δ* 3.72 - 3.69 (1H, m, Hβ), 2.50 - 2.46 (1H, m, Hα), 1.55 - 1.48 (2H, m, aliphatic), 1.40 - 1.30 (12H, m, aliphatic), 1.12 (3H, d, *J* = 7.1 Hz, -αCH_3), 0.90 (3H, t, *J* = 7.0 Hz, -CH_3). ^{13}C NMR (151 MHz; MeOD): δ 177.8, 72.6, 46.0, 33.4, 31.6, 29.32, 29.30, 29.0, 25.3, 22.3, 13.0, 12.2. HRMS *m/z* $[M+Na]^+$ calcd for $C_{12}H_{24}O_3Na$ 239.1624, found 239.1623.

Acid 20

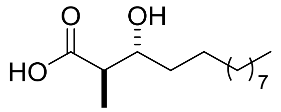

Compound **20** was synthesized according to the conditions outlined for acid **2**. Hydrogenation product **16** (93 mg, 0.23 mmol, 1 equiv.) was dissolved in THF (2.5 mL) and cooled to 0 °C. 30% aq. H_2O_2 solution (43 μL, 1.38 mmol, 6 equiv) and LiOH•H_2O (19 mg, 0.46 mmol, 2 equiv) were added and the reaction stirred at rt overnight. The crude mixture was quenched with sat. aq. Na_2SO_3 (100 μL) at 0 °C and concentrated *in vacuo*, before being resuspended in H_2O and washed with DCM (2 x 5 mL). The resulting aqueous layer was acidified to ~pH2 using 1M HCl and extracted with DCM (3 x 5 mL), and

the combined organic layers washed with brine and dried over anhydrous Na_2SO_4. Purification of the crude reaction mixture by flash chromatography resulted in co-elution, preventing isolation of acid **20**.

Acid 21

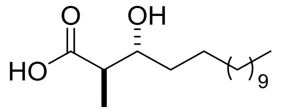

Compound **21** was synthesized according to the conditions outlined for acid **2**. Hydrogenation product **17** (120 mg, 0.28 mmol, 1 equiv.) was dissolved in THF (3 mL) and cooled to 0 °C. 30% aq. H_2O_2 solution (52 µL, 1.68 mmol, 6 equiv) and LiOH•H_2O (23 mg, 0.56 mmol, 2 equiv) were added and the reaction stirred at rt overnight. The crude mixture was quenched with sat. aq. Na_2SO_3 (100 µL) at 0 °C and concentrated *in vacuo*, before being resuspended in H_2O and washed with DCM (2 x 5 mL). The resulting aqueous layer was acidified to ~pH2 using 1M HCl and extracted with DCM (3 x 5 mL), and the combined organic layers washed with brine and dried over anhydrous Na_2SO_4. Purification of the crude reaction mixture by flash chromatography resulted in co-elution, preventing isolation of acid **21**.

Fmoc-Gly-2CT resin (22)

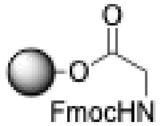

To a manual SPPS vessel was added 2-chlorotrityl (CT) chloride resin (500 mg, 1.0 equiv), followed by a solution of Fmoc-Gly-OH (803 mg, 2.7 mmol, 3.0 equiv) and DIPEA (550 µL, 3.15 mmol, 3.5 equiv) in dry DMF (10 mL). The suspension was agitated under argon for 18 h before being flushed and washed with DMF (3 x 10 mL) and DCM (3 x 10 mL). Unreacted resin sites were capped by bubbling the resin in a capping solution of MeOH:DIPEA:DCM (10:5:85, 10 mL) for 1 h. The resin was then flushed, washed with DCM (3 x 10 mL) and dried under a steady stream of argon. An estimate of resin loading was carried out using a literature Fmoc-loading test[2]. A small amount of resin was tested for loading (~5 - 10

mg) by liberation of the Fmoc-group in 2% DBU/DMF solution (2 mL) with 30 minutes agitation. This test solution was then diluted to 10 mL in MeCN and an aliquot (2 mL) diluted further to 25 mL in MeCN. The absorbance of both test and reference solutions (no resin) were recorded at 304 nm to obtain two sets of absorbance readings from which the loading could be deduced.

$$Fmoc\ loading\ (mmol \cdot g^{-1}) = (Abs_{sample} - Abs_{ref})\ x\ (16.4\ /\ mg\ of\ resin)$$

Pentapeptide 23

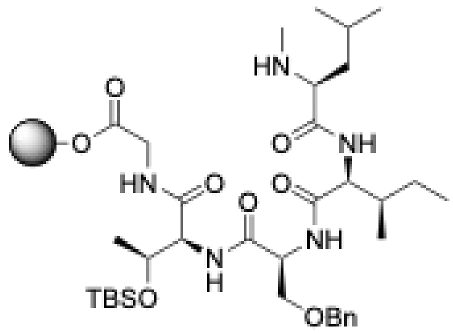

Compound **23** was synthesized according to a previously reported literature procedure[3]. To a manual SPPS vessel was added 2-chlorotrityl resin pre-loaded with Fmoc-glycine (200 mg, 0.1 mmol, 1.0 equiv). The resin was swelled in DCM (6 mL) for 30 min before the solvent was discharged and the Fmoc-protecting group removed using 20% 4-methylpiperidine (4-MP) in DMF solution (3 x 6 mL; 10 min). DMF washes followed each deprotection cycle (3 x 6 mL), with a final dry DMF wash carried out before the next amino acid coupling. A coupling solution of Fmoc-*a*Thr(OTBS)-OH[3] (92 mg, 0.2 mmol, 2.0 equiv), HOBt (31 mg, 0.2 mmol, 2.0 equiv) and DIC (39 µL, 0.25 mmol, 2.5 equiv) in dry DMF (3 mL) was prepared, and once pre-activated (5 min), was added to the SPPS vessel and the resulting suspension agitated under argon for 24 h. Upon completion of the coupling reaction, the resin was drained and washed with dry DMF (5 x 3 mL). The aforementioned Fmoc-deprotection cycle was again carried out before the next amino acid coupling. Fmoc-Ser(Bzl)-OH (125 mg, 0.3 mmol, 3.0 equiv), HOBt (46 mg, 0.3 mmol, 3.0 equiv) and DIC (55 µL, 0.35 mmol, 3.5 equiv) were dissolved in dry DMF (3 mL) and added to the reaction vessel. After 24 h agitation under argon, the resin was drained and washed with dry DMF (5 x 3 mL), before another round of Fmoc-deprotection. The next coupling solution of

Fmoc-*a*Ile-OH (71 mg, 0.2 mmol, 2.0 equiv), HOBt (31 mg, 0.2 mmol, 2.0 equiv) and DIC (39 µL, 0.25 mmol, 2.5 equiv) in dry DMF (3 mL) was prepared and added to the reaction vessel for 24 h agitation under argon. The resin was then drained, washed and the Fmoc-protecting group removed before the final amino acid coupling. Fmoc-*N*-Me-Leu-OH (92 mg, 0.25 mmol, 2.5 equiv), HOBt (38 mg, 0.25 mmol, 2.5 equiv) and DIC (50 µL, 0.32 mmol, 3.2 equiv) were dissolved in dry DMF (3 mL) and added to the reaction vessel. After 24 h agitation under argon, the resin was drained and washed with dry DMF (5 x 3 mL), DCM (3 x 3 mL), MeOH (3 x 3 mL) and Et_2O (3 x 3 mL), before being dried under a steady stream of argon. A microcleavage was performed to confirm pentapeptide **23** had been formed – a few beads of resin (10 mg) were shaken in 20% HFIP/DCM (2 mL) for 2 h, filtered through glass wool and then concentrated for HPLC and MS analysis. HPLC analysis yielded a single peak at t_R 14.0 min (see **Figure S63**) that was identified as the TBS deprotected analogue of pentapeptide **23** ($[M+H]^+$ calcd for $C_{44}H_{57}N_5O_{10}$ 816.4185, found 816.4337) due the acid lability of silyl protecting groups under acidic conditions.

Acylated pentapeptide 24

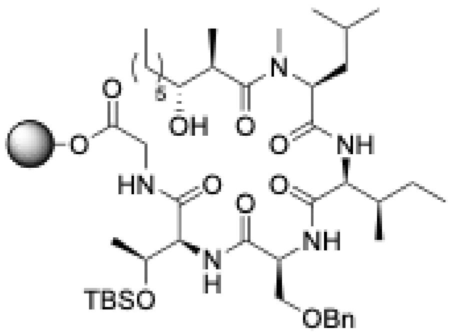

Resin-bound peptide **24** was synthesized according to a previously reported literature procedure[3]. Resin-bound pentapeptide **23** (0.095 mmol) was swollen in DCM (6 mL) for 30 min and then drained. The N-terminal Fmoc-protecting group was removed with a solution of 4-MP in DMF (3 x 6 mL, 10 min), and washed with dry DMF following the final deprotection step (3 x 6 mL). A solution of acid **2** (54 mg, 0.29 mmol, 3.0 equiv), triethylamine (40 µL, 0.29 mmol, 3.0 equiv) and DEPC (43 µL, 0.29 mmol, 3.0 equiv) in dry DMF (4 mL) was prepared and added to the reaction vessel. The resulting suspension was

agitated under argon for 18 h, before the solvent was discharged and the resin washed with dry DMF (3 x 5 mL) and dry DCM (3 x 5 mL). This lipid coupling step was repeated for another 18 h and the resin again drained and washed after such time. A microcleavage was performed to identify the acylated pentapeptide intermediate in which a few beads of resin (10 mg) were shaken in 20% HFIP/DCM (2 mL) for 2 h, filtered through glass wool and then concentrated for HPLC and MS analysis. HPLC analysis yielded a major peak at t_R 13.3 min (see **Figure S64**) that was identified as acylated pentapeptide **24** ($[M+H]^+$ calcd for $C_{45}H_{79}N_5O_{10}Si$ 878.5674, found 878.6220).

Synthesis of globomycin (1) using a solution-phase cyclization

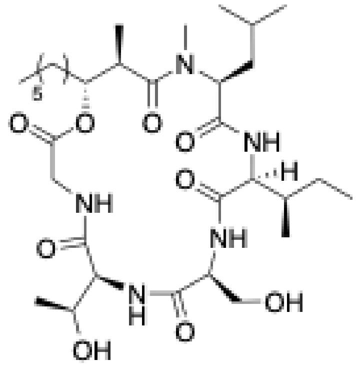

Globomycin (**1**) was synthesized according to a previously reported literature procedure[3]. Resin-bound precursor **24** (117 mg, 0.048 mmol) was swollen in DCM (6 mL) for 30 min and then drained. The linear precursor was then liberated from resin by agitating in a cleavage cocktail of DCM:AcOH:TFE (7:2:1, 3 mL) for 30 min under argon. The resin was drained and washed with DCM (2 x 3 mL) and the filtrate concentrated under reduced pressure to yield linear precursor as a white solid (33.6 mg, 0.038 mmol). This intermediate was dissolved in dry THF (1.7 mL) and stirred with dry triethylamine (20 µL, 0.14 mmol, 3.0 equiv.) and 2,4,6-trichlorobenzoyl chloride (9 µL, 0.058 mmol, 1.2 equiv.) for 18 h at rt. The generated acid anhydride was then diluted in dry toluene (23 mL) and over the next 5 h, added via syringe pump to a refluxed solution of 4-DMAP (117 mg, 0.96 mmol, 20 equiv.) in dry toluene (25 mL). The mixture was refluxed for a further 1.5 h and once cooled, the solvents were removed under reduced pressure and the resulting crude solid diluted with EtOAc and washed sequentially with 1M HCl and saturated $NaHCO_3$ and brine solutions. The organic layer was dried over anhydrous $NaSO_4$, filtered and

the solvent evaporated under reduced pressure to yield the crude product as an orange-brown solid. The protected macrolactone (0.043 mmol) was dissolved in dry THF (2.7 mL) with acetic acid (190 µL, 3.2 mmol, 75 equiv.) and tetra-*n*-butylammonium fluoride (2.34 mL, 2.8 mmol, 54 equiv.) and the mixture stirred at rt for 18 h. The crude mixture was then diluted with EtOAc, washed with saturated $NaHCO_3$ and brine solutions, dried over anhydrous $NaSO_4$ and concentrated under reduced pressure. The resulting solid was dissolved in dry MeOH (12 mL, 3.6 mM) and $Pd(OH)_2$ (20 mg, 20 wt%) added under argon. Once purged under argon, the system was then saturated with H_2 and stirred at rt under a positive pressure of H_2 for 18 h. Upon completion of the final deprotection step, the system was purged of H_2 and the crude mixture diluted in MeOH (3 mL) for catalyst removal via filtration. The crude peptide mixture was concentrated under reduced pressure and purified via RP-HPLC using the method outlined in 'peptide purification'. Product-containing fractions were concentrated under reduced pressure, frozen and lyophilized to yield depsipeptide **1** as a white solid (3 mg, 10% from Fmoc-Gly-CT).

Fmoc-*allo*-Thr-OAll DiPh resin (27)

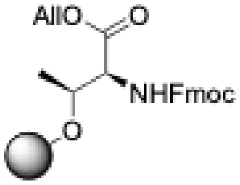

4-Methoxybenzhydryl bromide resin was loaded with Fmoc-*allo*-Thr-OAllyl according to a previously reported literature method[4]. Initially, unloaded resin (3.1 g, 5.2 mmol, 1.0 equiv) was added to a manual SPPS vessel and washed with DMF (5 x 10 mL) and DCM (10 x 10 mL), before being swollen in DCM (10 mL) for 1 h in the absence of light. The solvent was discharged and a solution of Fmoc-*allo*Thr-OAll (2.0 g, 5.2 mmol, 1.0 equiv) and DIPEA (5.5 mL, 31.4 mmol, 6.0 equiv) in DCM (30 mL) added and the resin agitated under argon for 48 h. The resin was then drained and washed with DCM (5 x 10 mL), DMF (10 x 10 mL) and DCM (3 x). Unreacted sites were capped using a solution of acetic anhydride/pyridine (1:9 by volume, 30 mL) under argon for 30 min. After flushing the capping solution, DCM (5 x 10 mL), DMF (5 x 10 mL) and DCM (2 x) washes were performed, before the resin was left to dry under a steady stream of argon to obtain the β-hydroxy loaded resin (**27**; loading = 0.23 mmol/g).

Tetrapeptide 28

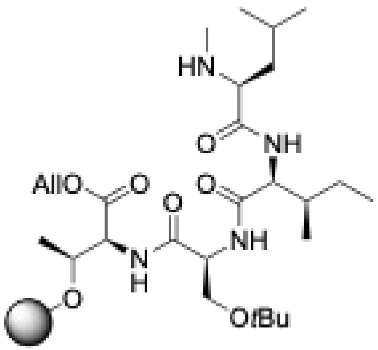

Linear tetrapeptide **28** was synthesised from *a*Thr-OAllyl building block **27** using standard HATU/DIPEA coupling conditions. To this end, resin **27** (3g, 0.24 mmol, 1.0 equiv) was added to a manual SPPS vessel and swelled in DCM (30 mL) for 30 min. The resin was drained and washed with DMF (3 x 30 mL) before being subjected to Fmoc-deprotection cycles with 20% 4-MP, DMF solution (3 x 30 mL; 2 x 1 min, 1 x 10 min). Deprotections were monitored under UV and DMF washes were performed after each deprotection cycle (3 x 30 mL). Once Fmoc removal was complete, amino acids were sequentially coupled by dissolving the respective Fmoc-aa-OH (5 equiv.) in DMF (30 mL) alongside HATU (5 equiv.) and DIPEA (10 equiv.). Iterative SPPS cycles were carried out under argon for 2 h each to yield resin bound tetrapeptide **28**. A portion of resin (500 mg, 0.04 mmol) was cleaved for characterisation by suspending in a cleavage cocktail of TFA:TIPS:H_2O (95:2.5:2.5, 10 mL) at 40 °C for 2 h. The cleaved resin was then filtered through glass wool and the filtrate concentrated under reduced pressure to form a yellow oil that was then precipitated in cold diethyl ether at 0 °C. After 1 h, the suspension was centrifuged and the supernatant decanted off. The remaining pellets were resuspended in cold diethyl ether and the process repeated again. Crude peptide pellets were dissolved in 30% MeCN/H_2O (+0.1% TFA) and purified via RP-HPLC to yield tetrapeptide **28** as a white powder (11 mg, 56%); ^{1}H NMR (600 MHz; DMSO-d_6) δ 8.80 (1H, br. s, -*N*MeLeu-N<u>H</u>), 8.65 (1H, d, *J* = 9.2 Hz, Ile-N<u>H</u>), 8.20 (1H, d, *J* = 7.8 Hz, Ser-N<u>H</u>), 8.09 (1H, d, *J* = 8.2 Hz, aThr-N<u>H</u>), 5.88 (1H, ddt, *J* = 17.2, 10.6, 5.3 Hz, -OCH$_2$C<u>H</u>), 5.33 (1H, dd, *J* = 17.3, 1.7 Hz, -OCH$_2$CHC<u>H</u>=CH), 5.19 (1H, dd, *J* = 10.6, 1.5 Hz, -OCH$_2$CHCH=C<u>H</u>), 5.05 (1H, d, *J* = 5.8 Hz, *a*Thr-O<u>H</u>), 4.91 (1H, t, *J* = 5.3 Hz, Ser-O<u>H</u>), 4.56 (2H, m, -OC<u>H$_2$</u>CH), 4.40 (1H, dd, *J* = 8.7, 7.8 Hz, Ile-α<u>H</u>), 4.37 (1H, q, *J* = 6.8 Hz, Ser-α<u>H</u>), 4.25 (1H, dd, *J* = 8.2, 6.8 Hz, *a*Thr-α<u>H</u>), 3.86

(1H, dq, J = 12.6, 6.3 Hz, aThr-β<u>H</u>), 3.81 (1H, m, -*N*MeLeu-α<u>H</u>), 3.53 (2H, app. t, J = 5.6 Hz, Ser-β<u>H</u>), 2.44 (3H, s, -*N*MeLeu-C<u>H</u>$_3$), 1.78 (1H, m, Ile-β<u>H</u>), 1.61 – 1.54 (2H, m, -*N*MeLeu-β<u>H</u> and γ<u>H</u>), 1.51 (1H, m, -*N*MeLeu-β<u>H</u>), 1.44 (1H, m, Ile-γ<u>H</u>), 1.14 (1H, t, J = 7.3 Hz, Ile-γ<u>H</u>), 1.10 (3H, d, J = 6.4 Hz, *a*Thr-C<u>H</u>$_3$), 0.89 (3H, d, J = 6.2 Hz, -*N*MeLeu-γC<u>H</u>$_3$), 0.87 – 0.85 (6H, m, -*N*MeLeu-γC<u>H</u>$_3$ & Ile-βC<u>H</u>$_3$), 0.83 (3H, t, J = 7.4 Hz, Ile-βCH$_3$). LC-MS, $[M+H]^+$ calcd for $C_{23}H_{42}N_4O_7H$ 487.3, found 487.1.

Acylated tetrapeptide 29

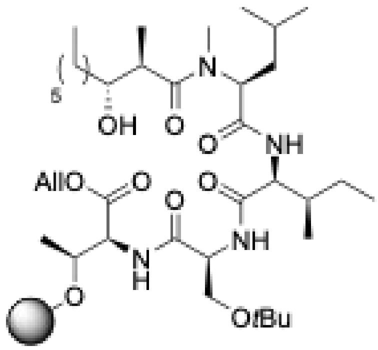

Resin-bound tetrapeptide **28** (1.23 mg, 0.1 mmol, 1.0 equiv.) was swollen in DCM (10 mL) for 30 min before coupling acid **2** to the liberated N-terminal amine. A solution of acid **2** (56 mg, 0.3 mmol, 3.0 equiv), triethylamine (42 µL, 0.3 mmol, 3.0 equiv) and DEPC (46 µL, 0.3 mmol, 3.0 equiv) in dry DMF (10 mL) was prepared and added to the reaction vessel. The resulting suspension was agitated under argon for 18 h, before the solvent was discharged and the resin washed with dry DMF (3 x 10 mL) and dry DCM (3 x 10 mL). This lipid coupling step was repeated for another 18 h and the resin again drained and washed. A microcleavage was performed to identify the acylated tetrapeptide intermediate in which a few beads of resin (10 mg) were shaken in 20% HFIP/DCM (2 mL) for 2 h, filtered through glass wool and then concentrated for HPLC and MS analysis. HPLC analysis yielded a major peak at 1.6 min that was identified as the allyl deprotected analogue of acylated tetrapeptide **29** ($[M+Na]^+$ calcd for $C_{34}H_{64}N_4O_9Na$ 695.5, found 695.9) owing to the acid lability of the allyl ester protecting group.

Unsuccessful synthesis of globomycin (1) by solid-phase cyclization

To resin-bound acylated tetrapeptide **29** was added a coupling solution of Fmoc-Gly-OH (595 mg, 2 mmol, 20 equiv.), benzoyl chloride (235 µL, 2 mmol, 20 equiv.), 4-DMAP (10 mg, 0.08 mmol, 0.8 equiv.) and dry DIPEA (700 µL, 4 mmol, 40 equiv.) in dry DCM (10 mL). The suspension was stirred under a positive pressure of argon for 4 days before being filtered through a manual SPPS vessel and washed with dry DCM (3 x 10 mL) and dry DMF (3 x 10 mL). The Fmoc protecting group was then removed to facilitate on-resin macrolactonisation, but also served as an *in situ* monitoring technique to ascertain the success of the on-resin esterification step. To this end dry 20% 4-MP in DMF solution (3 x 10 mL) was bubbled through the resin beads for 5 min per deprotection cycle and each step monitored by UV. The liberated dibenzofulvene by-product did indeed decrease in UV intensity throughout the deprotection steps, indicating successful coupling of the glycine residue. After each deprotection step, the resin was washed with dry DMF (3 x 10 mL).

N and C-terminal protecting groups were next removed. The C-terminal allyl protecting group was removed by treating the resin (0.05 mmol, 1.0 equiv.) with a solution of $Pd(PPh_3)_4$ (58 mg, 0.05 mmol, 1.0 equiv.), $PhSiH_3$ (62 µL, 0.5 mmol, 10 equiv.) in dry DMF:DCM (1:1, 6 mL) for 2 h in the absence of light. After that time, the resin was drained and washed with dry DCM until the effluent changed from brown to colourless. A series of washing steps were initiated to sequester the palladium catalyst, including dry DMF (3 x 10 mL), dry 0.5% sodium diethyldithiocarbamate in DMF solution (5 x 10 mL), dry DMF (3 x 10 mL) and dry DCM (3 x 10 mL). The N-terminal Fmoc protecting group was then removed under basic conditions using a 20% solution of 2-MePip in DMF (3 x 6 mL; 5 min). The resin was washed between each deprotection step using dry DMF (3 x 6 mL) before removing the C-terminal allyl protecting group as described. The deprotected resin was then dried under a steady stream of argon and a microcleavage performed in 20% HFIP/DCM for 2 h to identify the presence of the deprotected linear precursor required for the final cyclization step. HPLC analysis (see **Figure S65**) revealed a mixture of the desired linear precursor **31** (LC-MS, $[M+H]^+$ calcd for $C_{36}H_{67}N_5O_{10}H$ 730.5, found 730.3), as well as a side-product in which the glycine ester moiety had been hydrolyzed and allyl group deprotected (LC-MS, $[M+H]^+$ calcd for $C_{34}H_{64}N_4O_9H$ 673.5, found 673.3). We were unable to

ascertain if this occurred due to incomplete Fmoc-Gly-OH coupling in the previous step, hydrolysis during subsequent basic Fmoc deprotections, or during the resin cleavage step. Therefore, it was decided to proceed with screening conditions for the final on-resin macrolactonisation step.

Resin-bound linear precursor **31** was split into four equal aliquots (0.024 mmol, 285 mg) and the following conditions screened for on-resin macrolactamisation. Upon completion of the cyclization step each resin was cleaved in a cocktail of TFA:TIPS:H_2O (95:2.5.2.5; 5 mL) at 40 °C for 2 h. Each crude mixture was then subjected to the same ether work-up as outlined for tetrapeptide **28** and analysed by HPLC and MS.

Reagent combinations screened for solid-phase cyclisation

**Cyclization Conditions**		**Yield (mg, ((%))**
**Reagents**	Conditions	
**BOP, DIPEA**	dry DMF, 18 h, rt	0 (0)
**DIC, HOBt, 4-DMAP (cat.)**	dry DMF/DCM (4:1), 18 h, rt	0 (0)
**DIC, HOBt, 4-DMAP (cat.)**	dry DMF/DCM (4:1), 18 h, rt	0 (0)
**HATU, DIPEA**	dry DMF, 18 h, rt	0 (0)

HPLC comparison of synthetic globomycin with natural globomycin

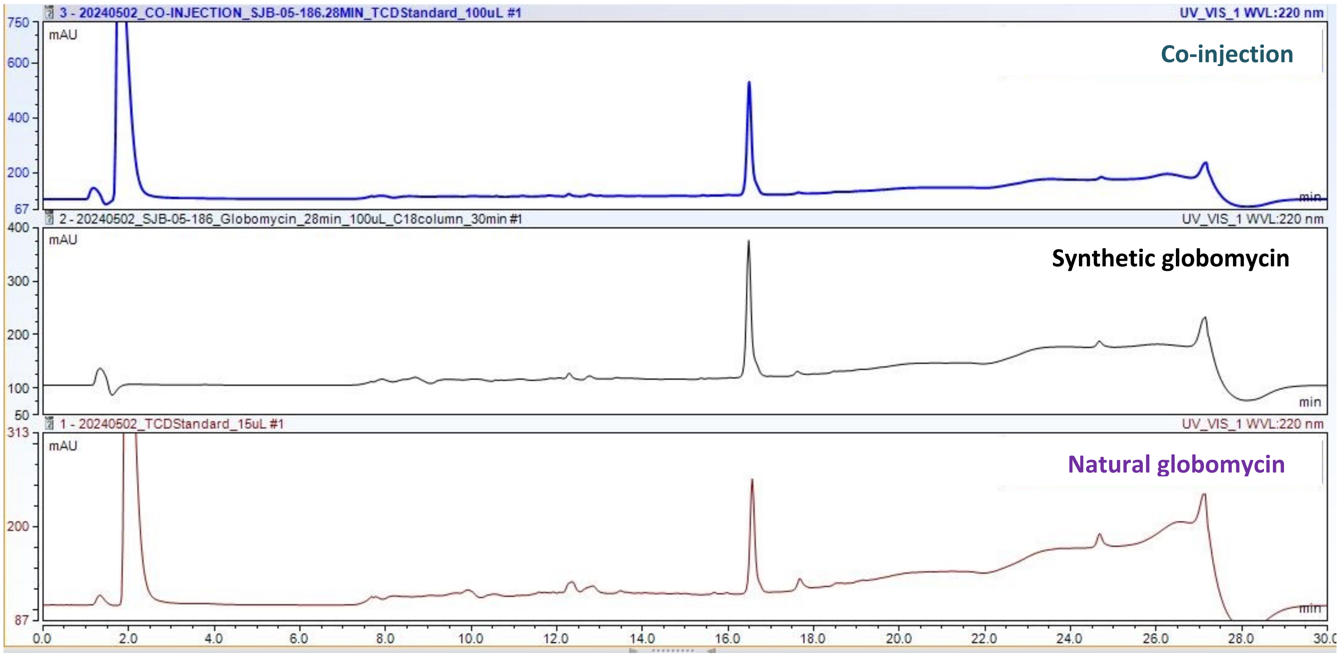

References

(1) Evans, D. A.; Tedrow, J. S.; Shaw, J. T.; Downey, C. W. Diastereoselective Magnesium Halide-Catalyzed Anti-Aldol Reactions of Chiral N-Acyloxazolidinones. J Am Chem Soc 2002, 124 (3), 392–393. https://doi.org/10.1021/ja0119548.

(2) Gude, M.; Ryf, J.; White, P. D. An Accurate Method for the Quantitation of Fmoc-Derivatized Solid Phase Supports. Letters in Peptide Science 2002, 9 (4–5), 203–206.

(3) Sarabia, F.; Chammaa, S.; García-Ruiz, C. Solid Phase Synthesis of Globomycin and SF-1902 A5. Journal of Organic Chemistry 2011, 76 (7), 2132–2144. https://doi.org/10.1021/jo1025145.

(4) Ficht, S.; Payne, R. J.; Guy, R. T.; Wong, C. H. Solid-Phase Synthesis of Peptide and Glycopeptide Thioesters through Side-Chain-Anchoring Strategies. Chemistry - A European Journal 2008, 14 (12), 3620–3629. https://doi.org/10.1002/chem.200701978.

Spectroscopic data of synthesized compounds

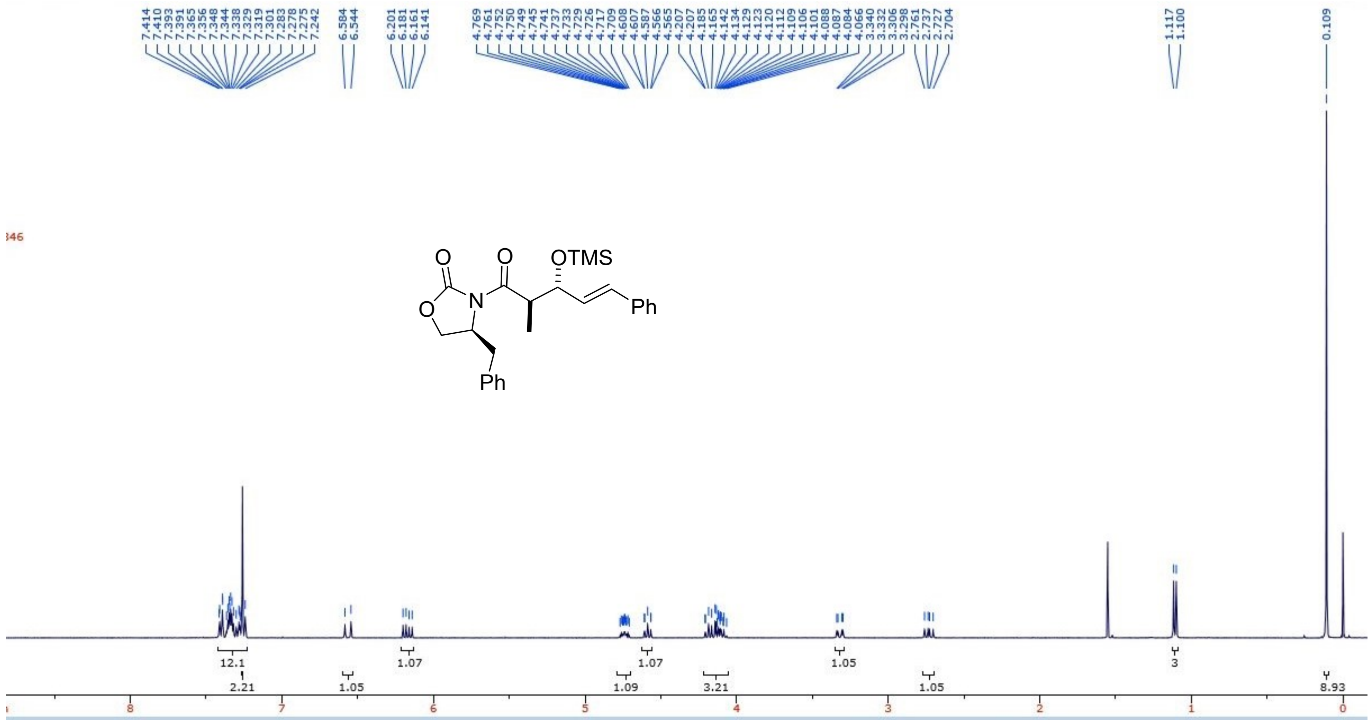

Figure S1: ^{1}H NMR spectrum (400 MHz, $CDCl_3$) of TMS ether **7**

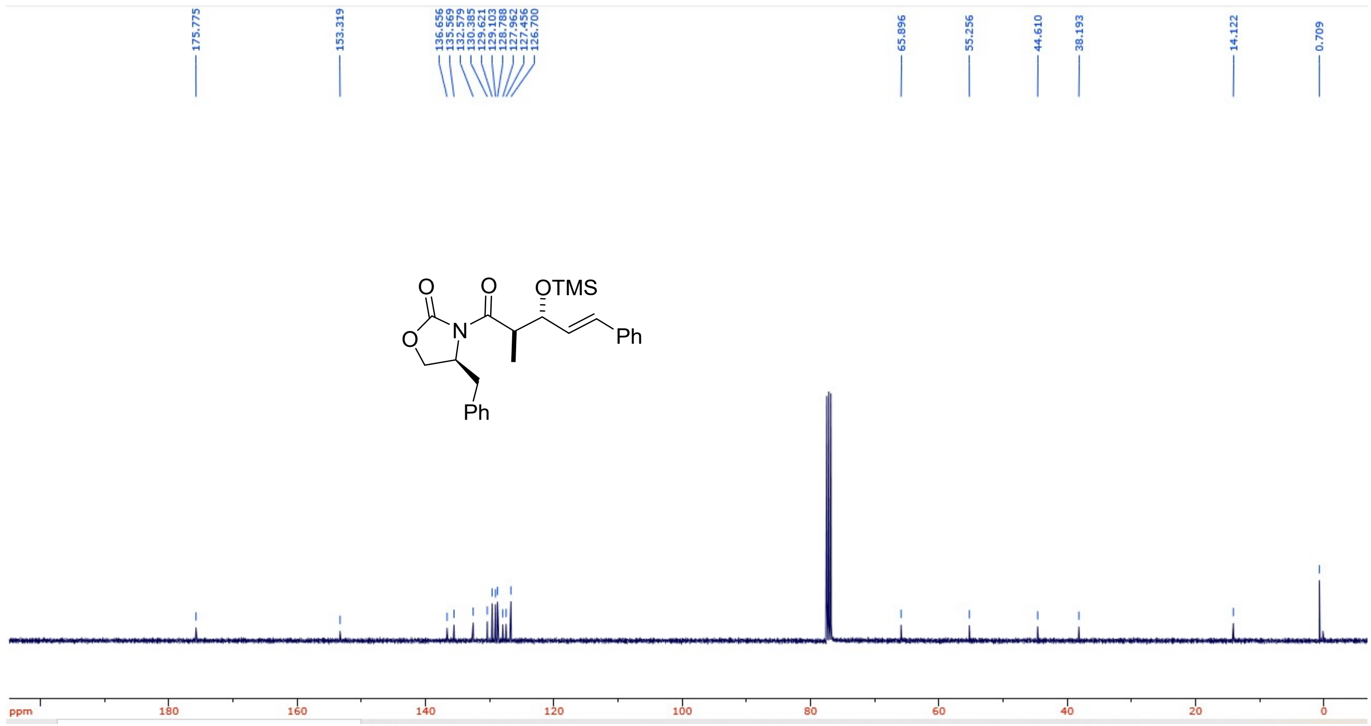

Figure S2: ^{13}C NMR spectrum (101 MHz, $CDCl_3$) of TMS ether **7**

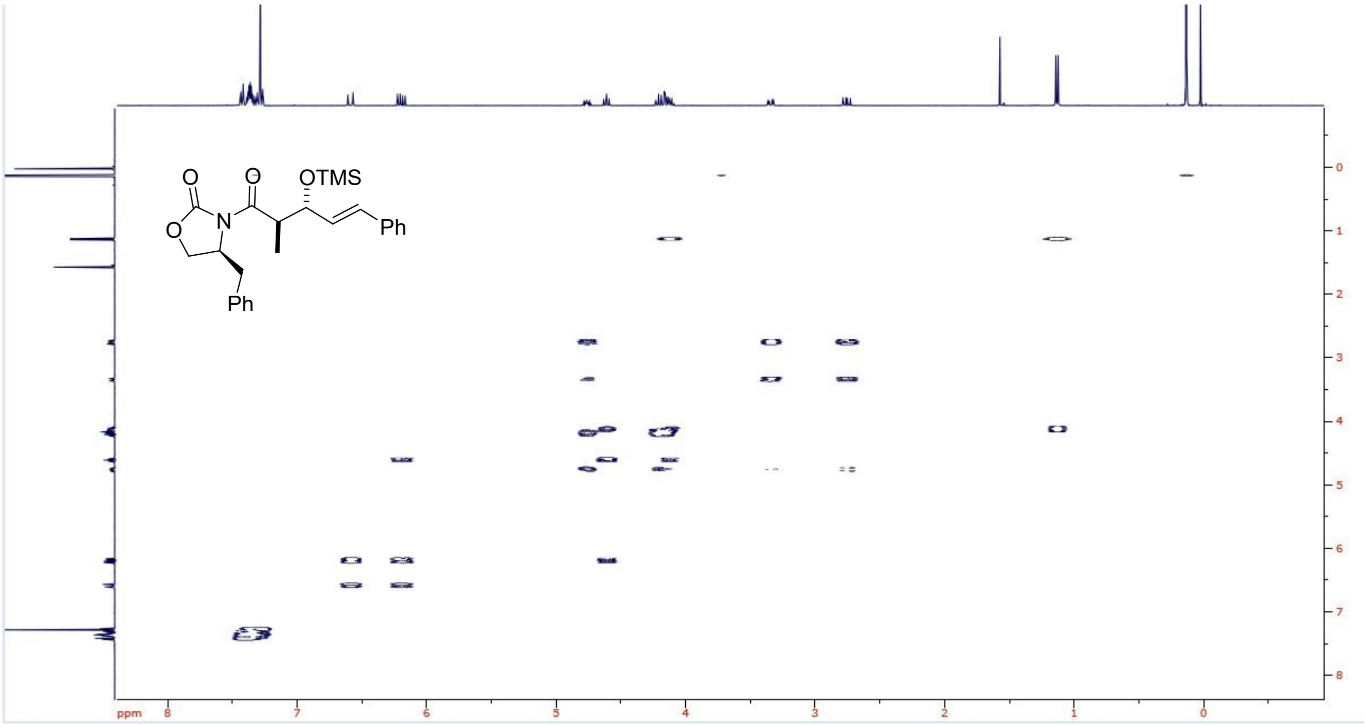

Figure S3: COSY spectrum ($CDCl_3$) of TMS ether **7**

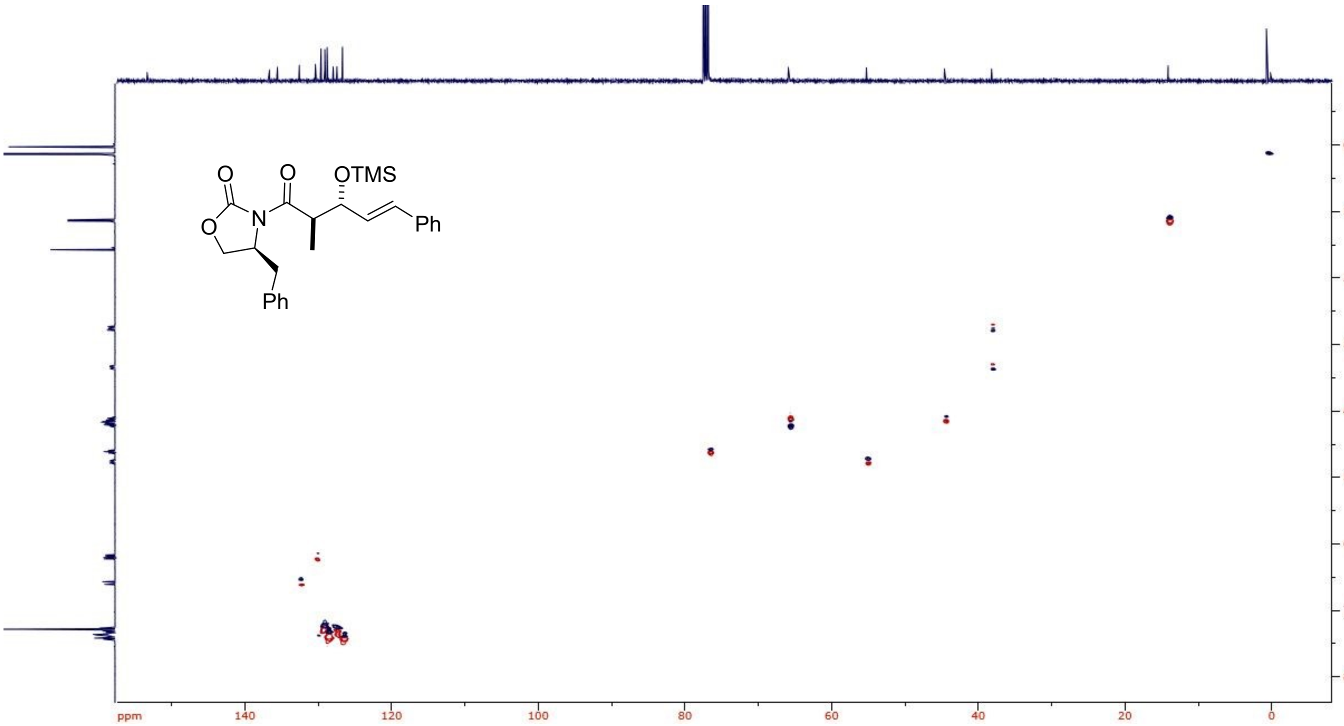

Figure S4: HSQC spectrum ($CDCl_3$) of TMS ether **7**

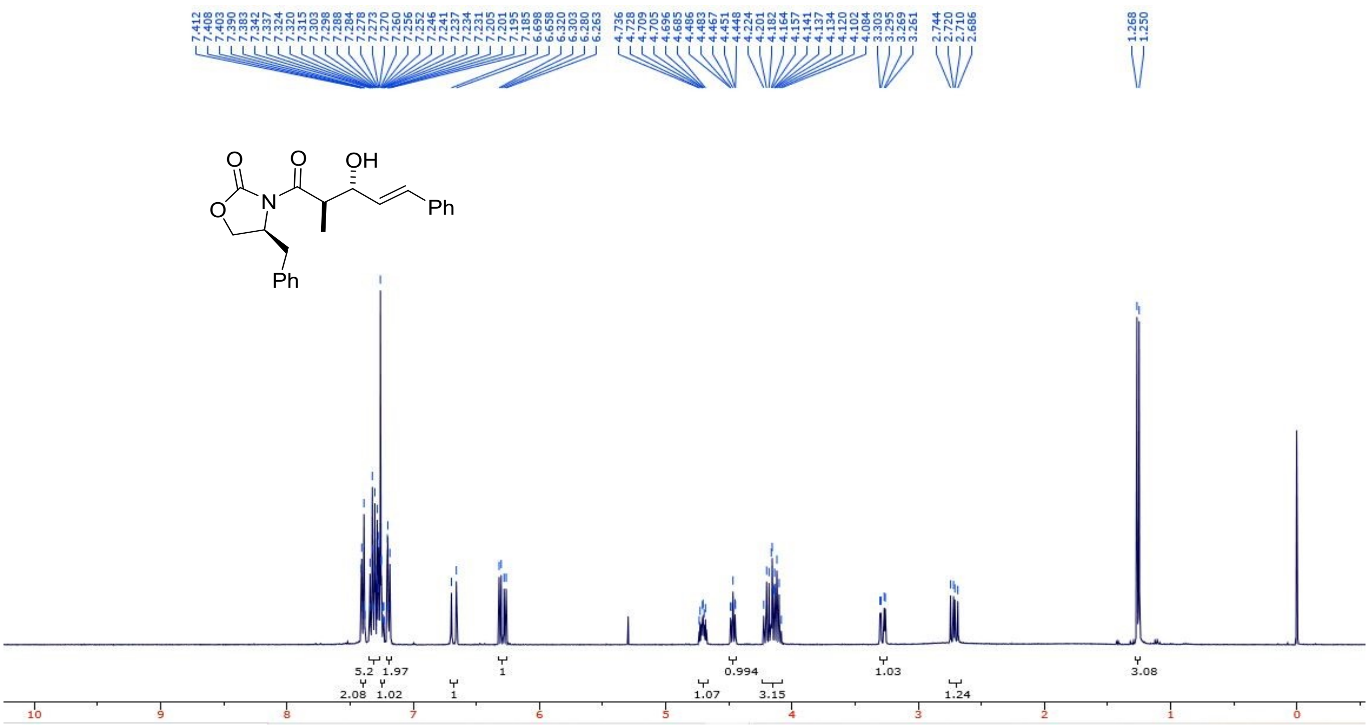

Figure S5: ^{1}H NMR spectrum (400 MHz, $CDCl_3$) of enol **6**

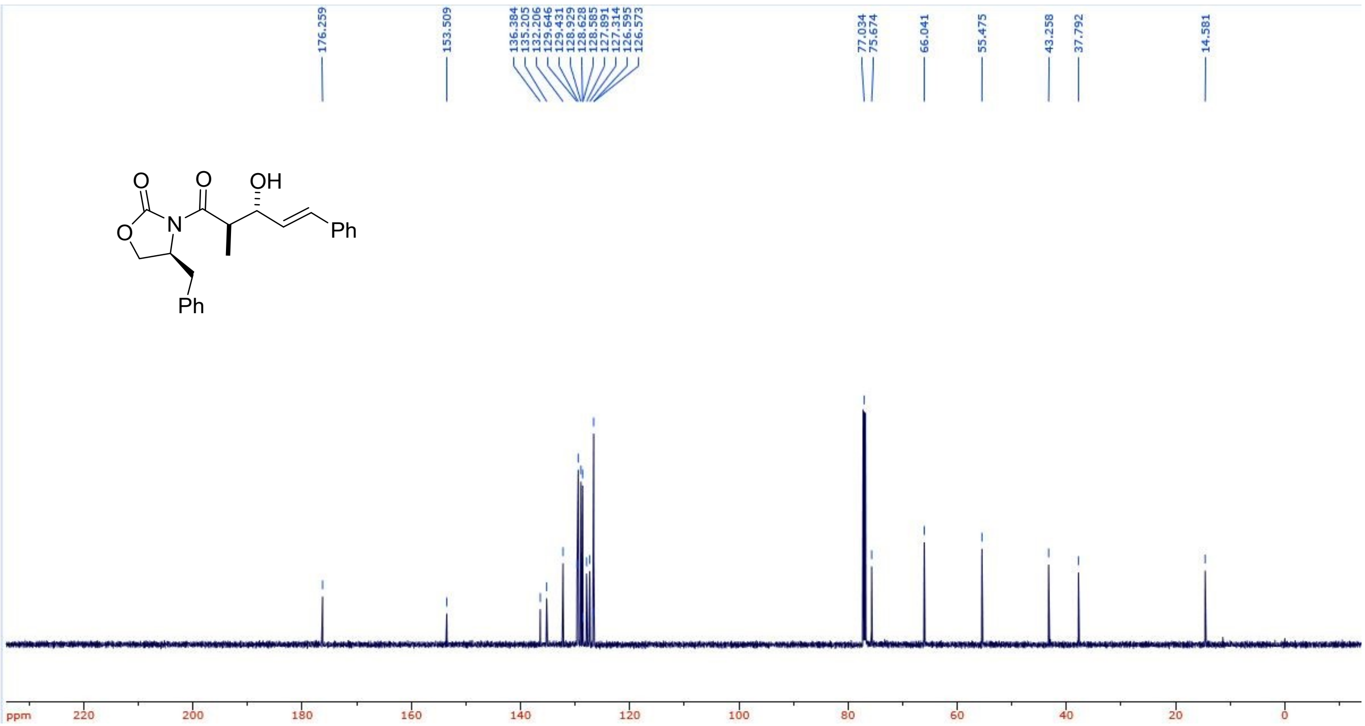

Figure S6: ^{13}C NMR spectrum (101 MHz, $CDCl_3$) of enol **6**

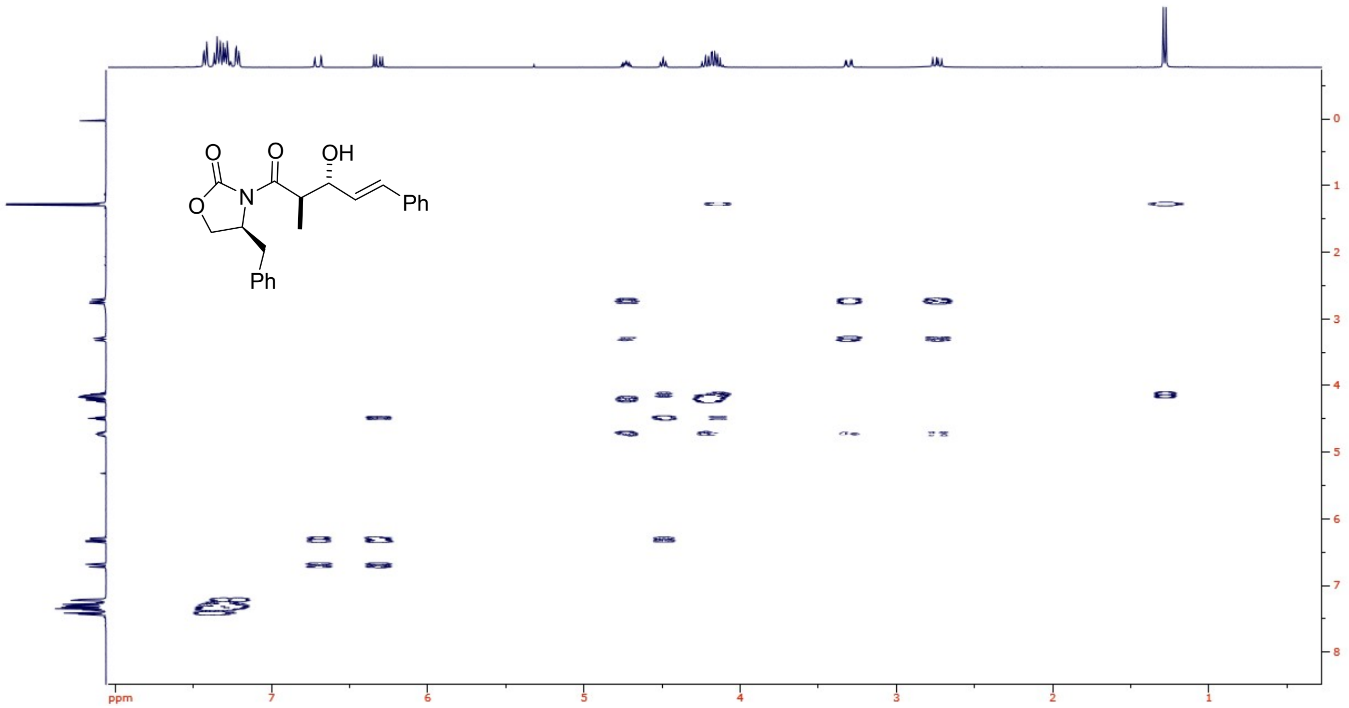

Figure S7: COSY spectrum ($CDCl_3$) of enol **6**

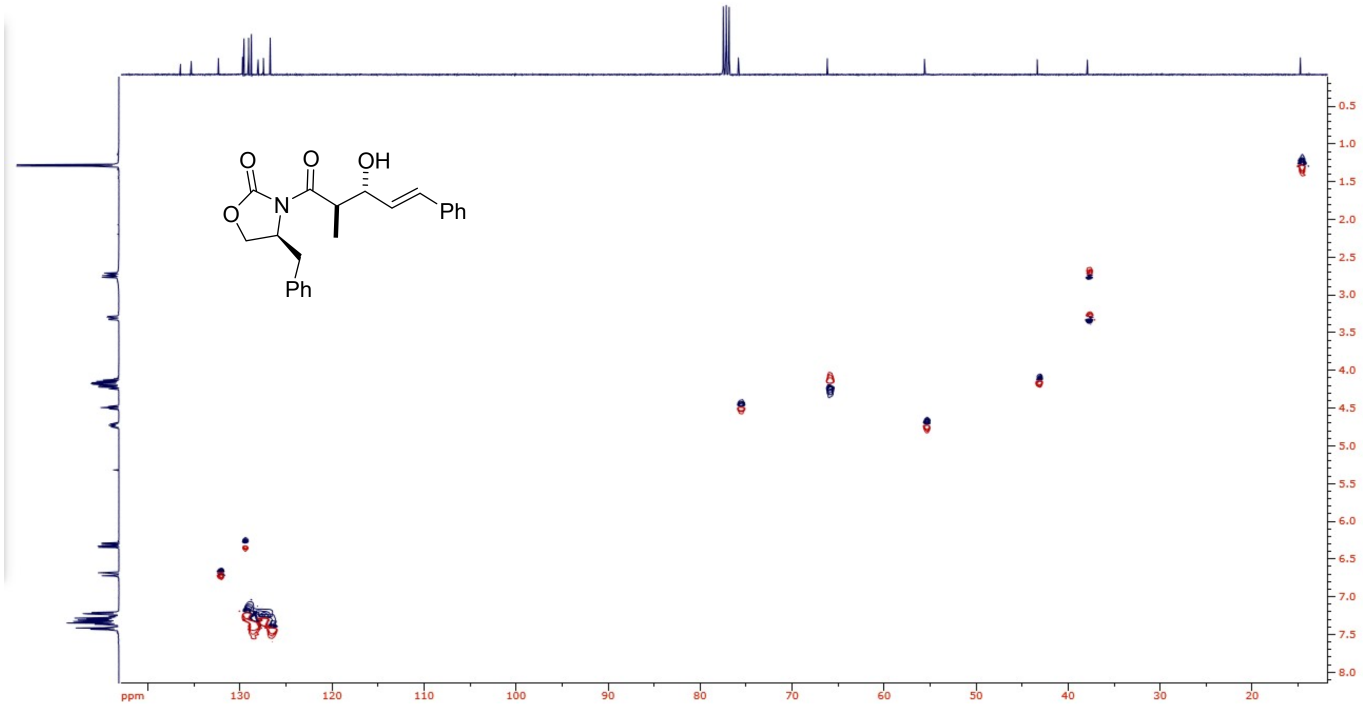

Figure S8: HSQC spectrum ($CDCl_3$) of enol **6**

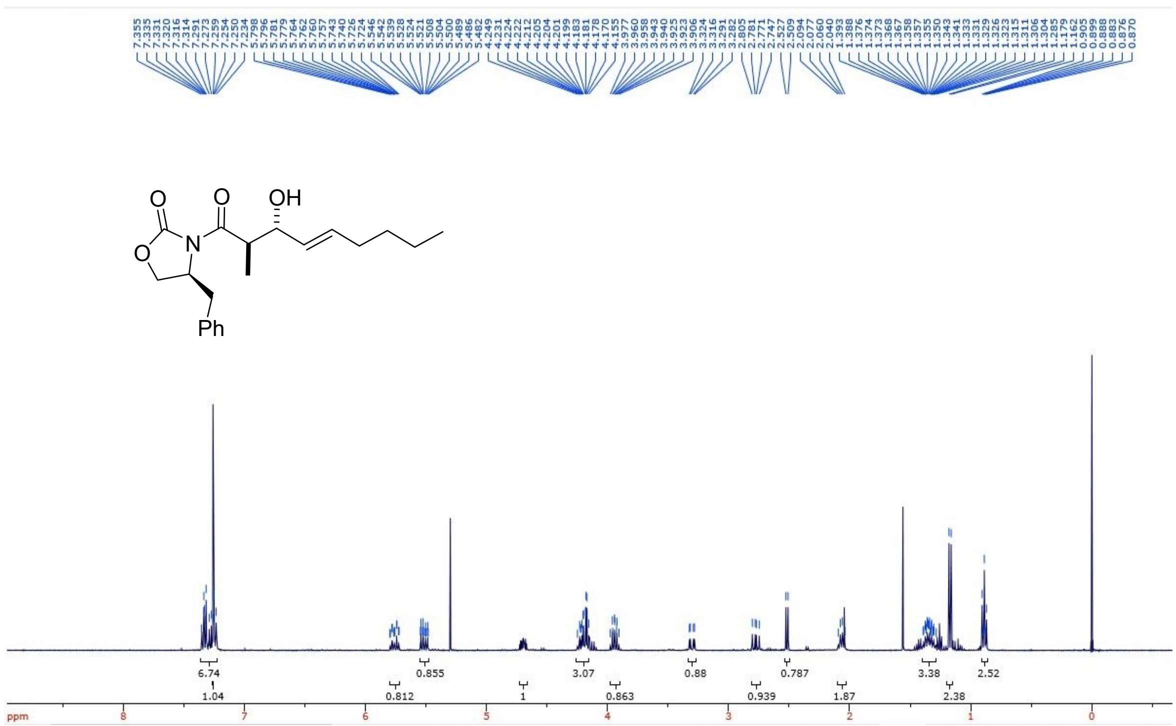

Figure S9: ^{1}H NMR spectrum (400 MHz, $CDCl_3$) of alkene **8**

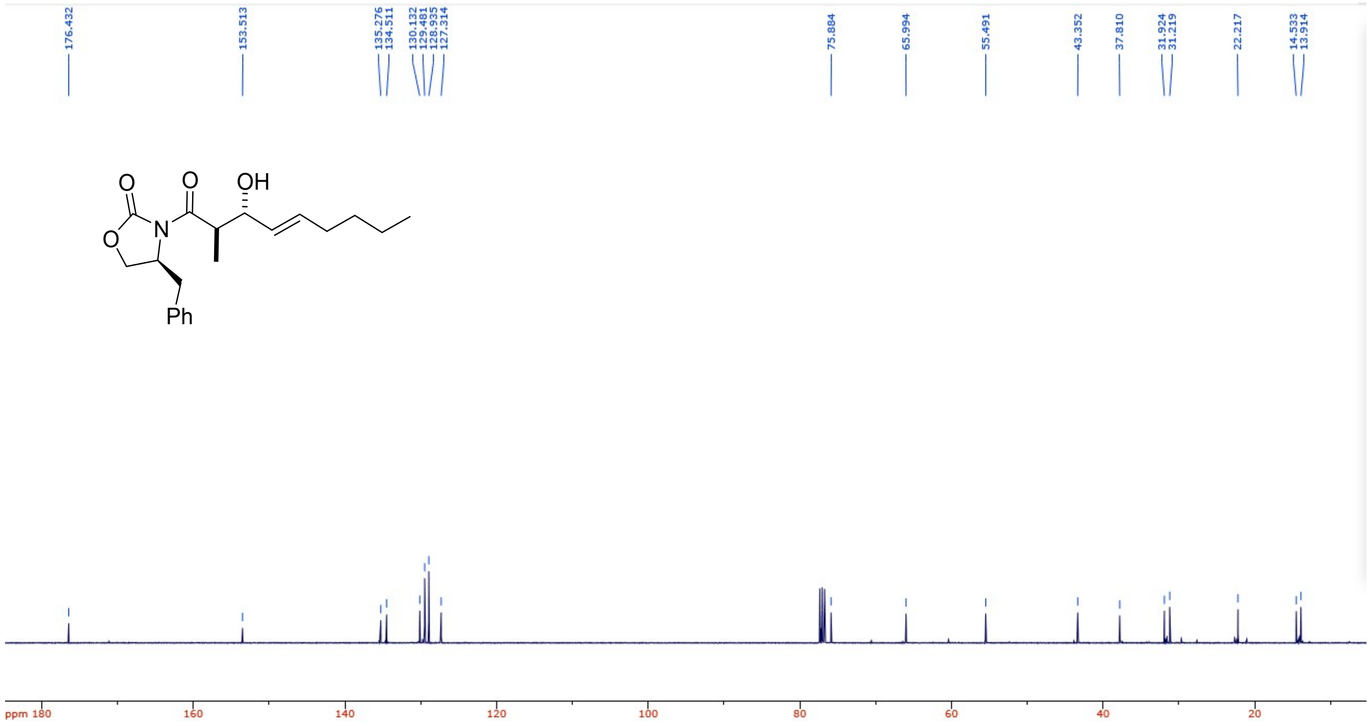

Figure S10: ^{13}C NMR spectrum (101 MHz, $CDCl_3$) of alkene **8**

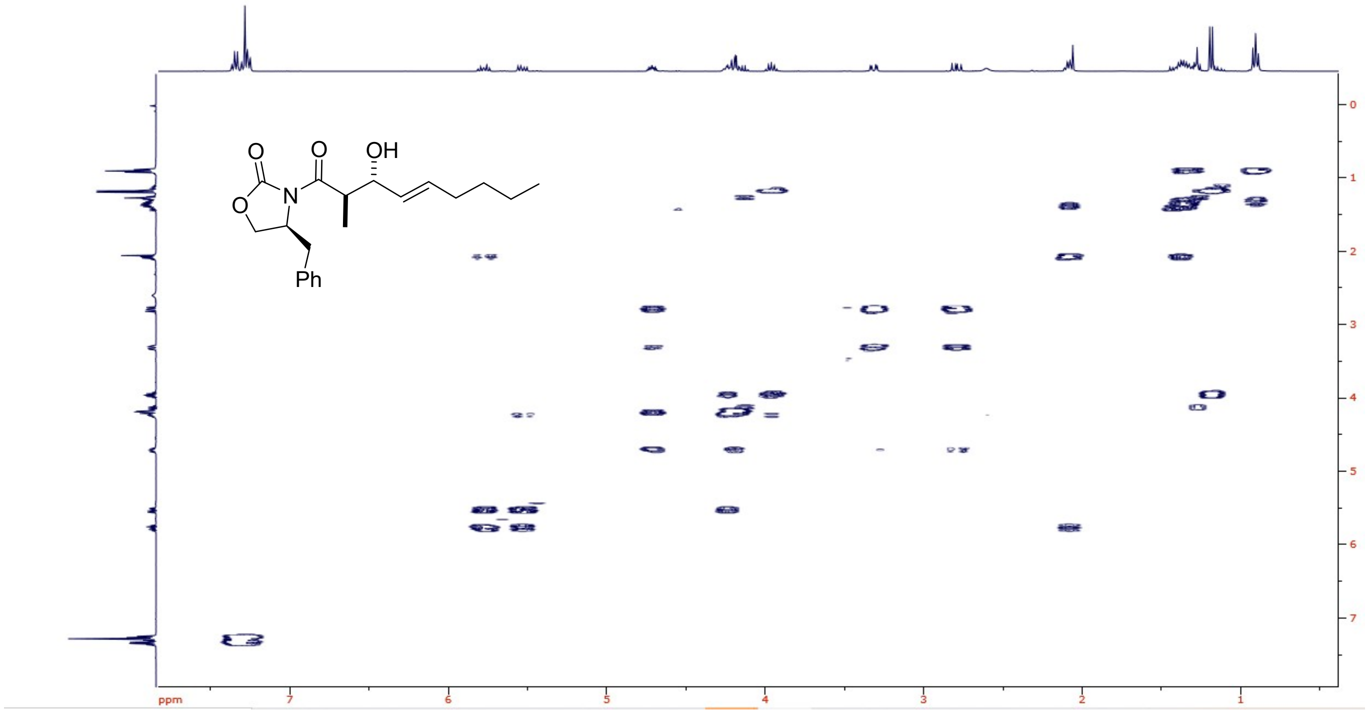

Figure S11: COSY spectrum ($CDCl_3$) of alkene **8**

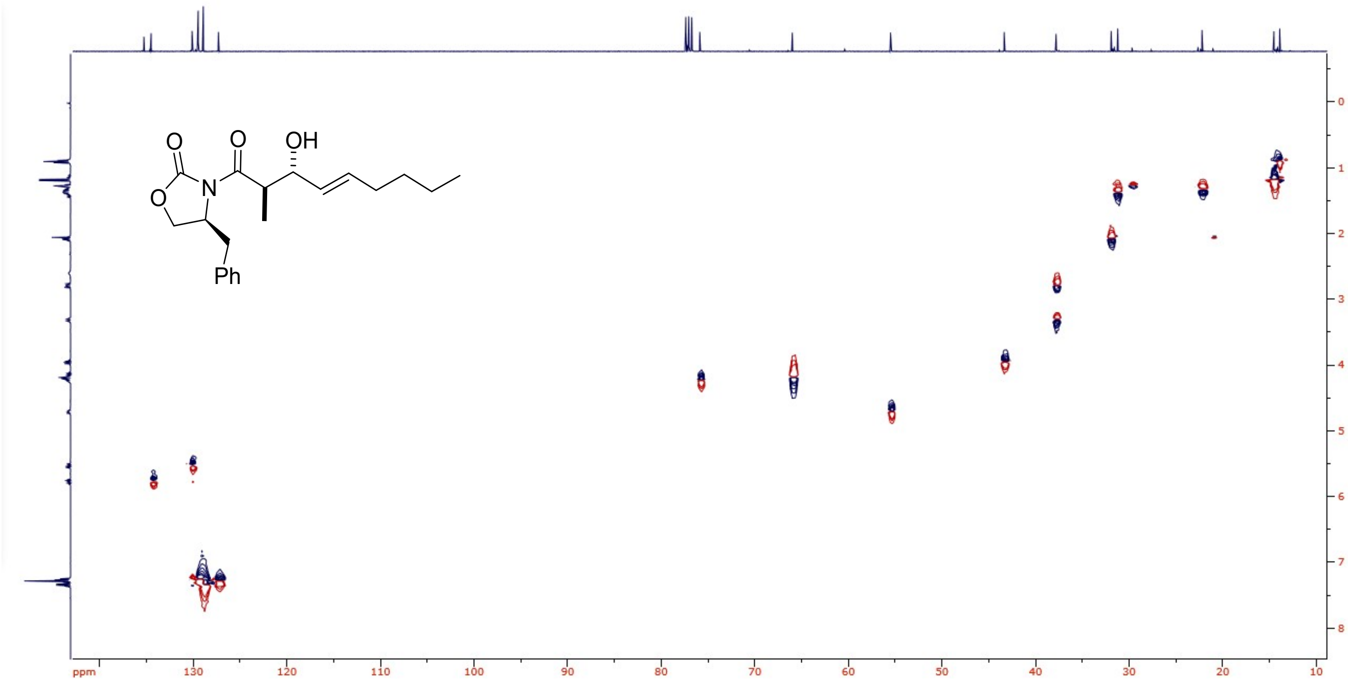

Figure S12: HSQC spectrum ($CDCl_3$) of alkene **8**

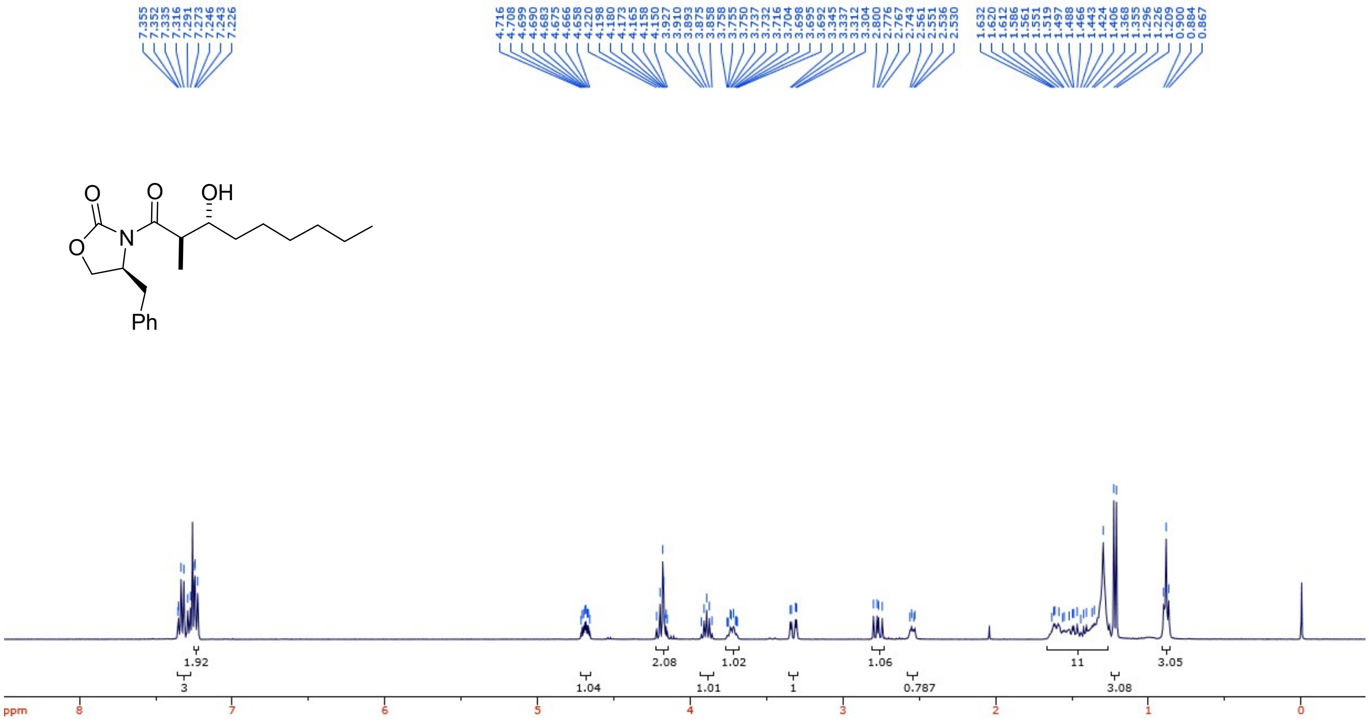

Figure S13: ^{1}H NMR spectrum (400 MHz, $CDCl_3$) of alkane **9**

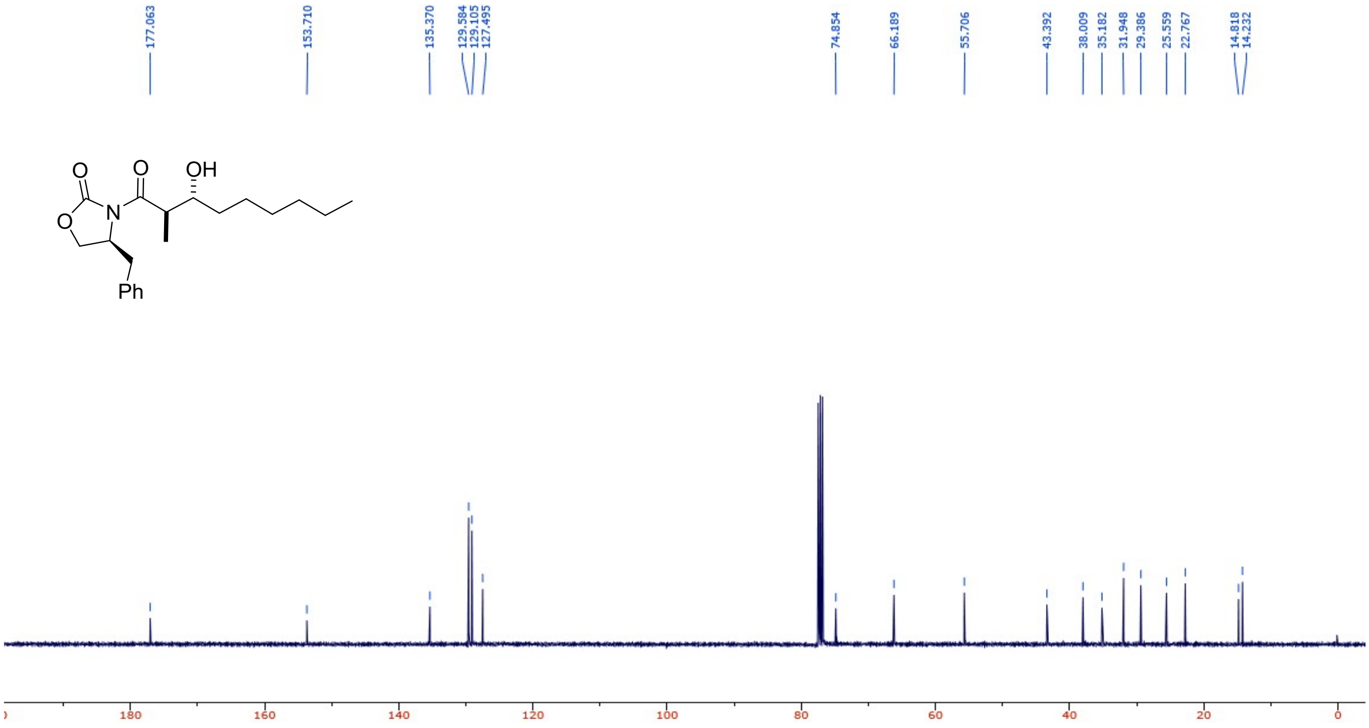

Figure S14: ^{13}C NMR spectrum (101 MHz, $CDCl_3$) of alkane **9**

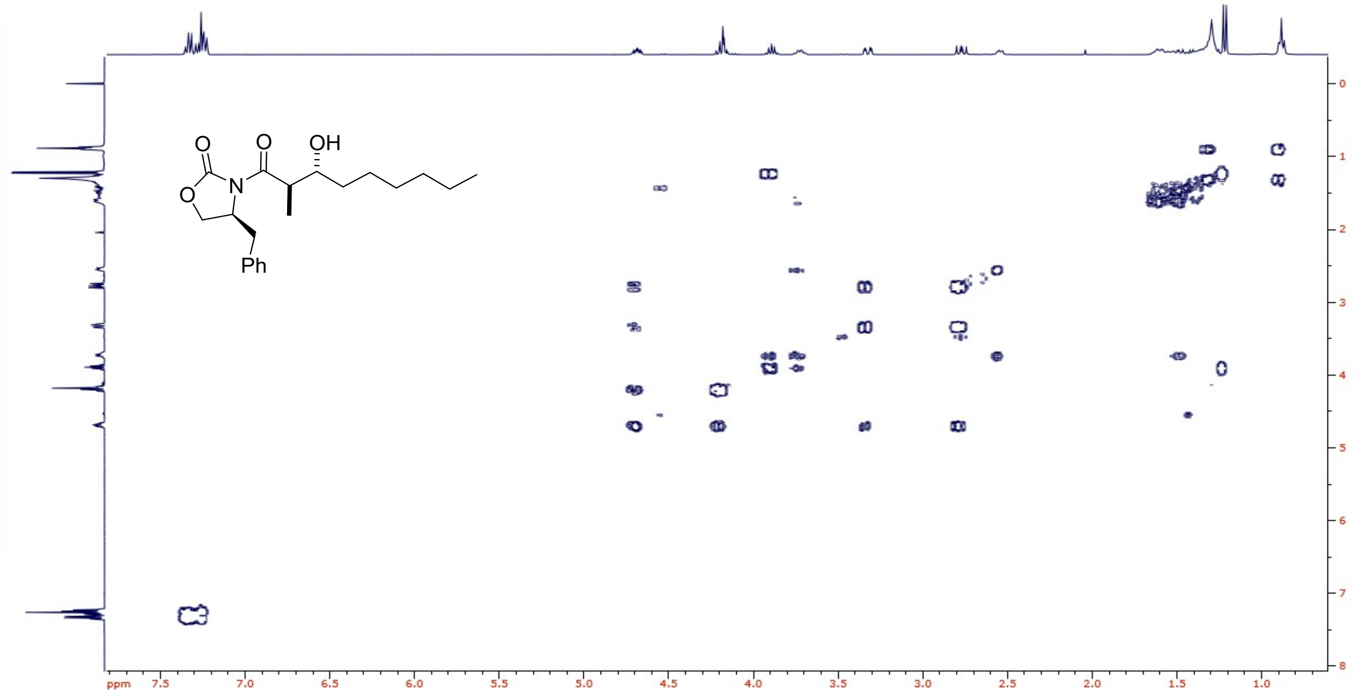

Figure S15: COSY spectrum ($CDCl_3$) of alkane **9**

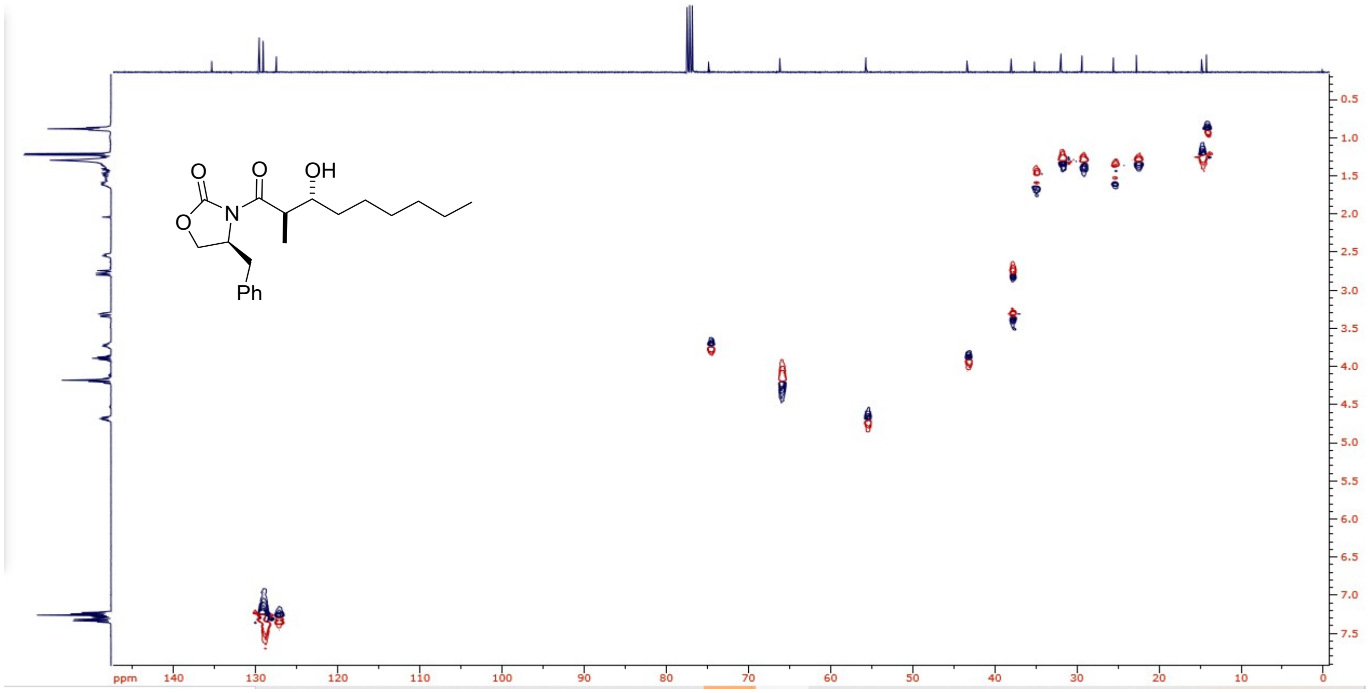

Figure S16: HSQC spectrum ($CDCl_3$) of alkane **9**

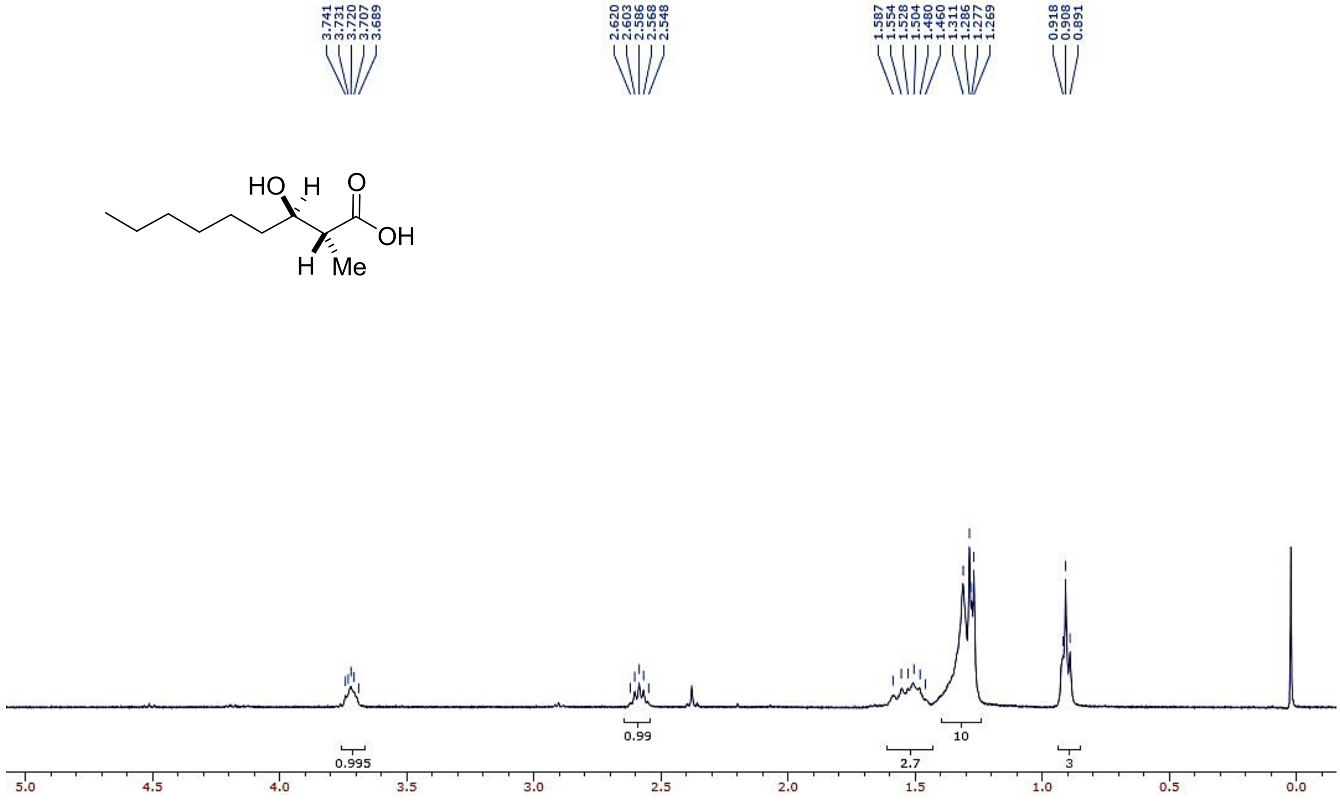

Figure S17: ^{1}H NMR spectrum (400 MHz, $CDCl_3$) of acid **2**

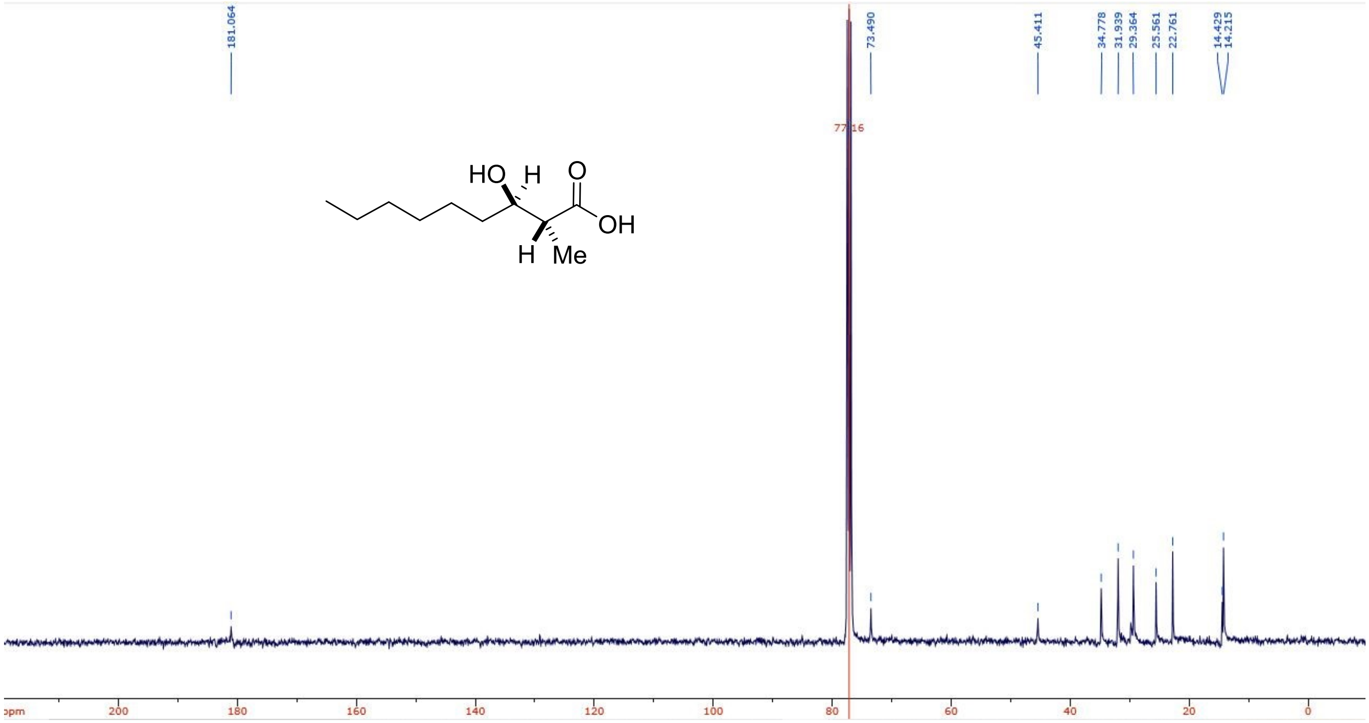

Figure S18: ^{13}C NMR spectrum (101 MHz, $CDCl_3$) of acid **2**

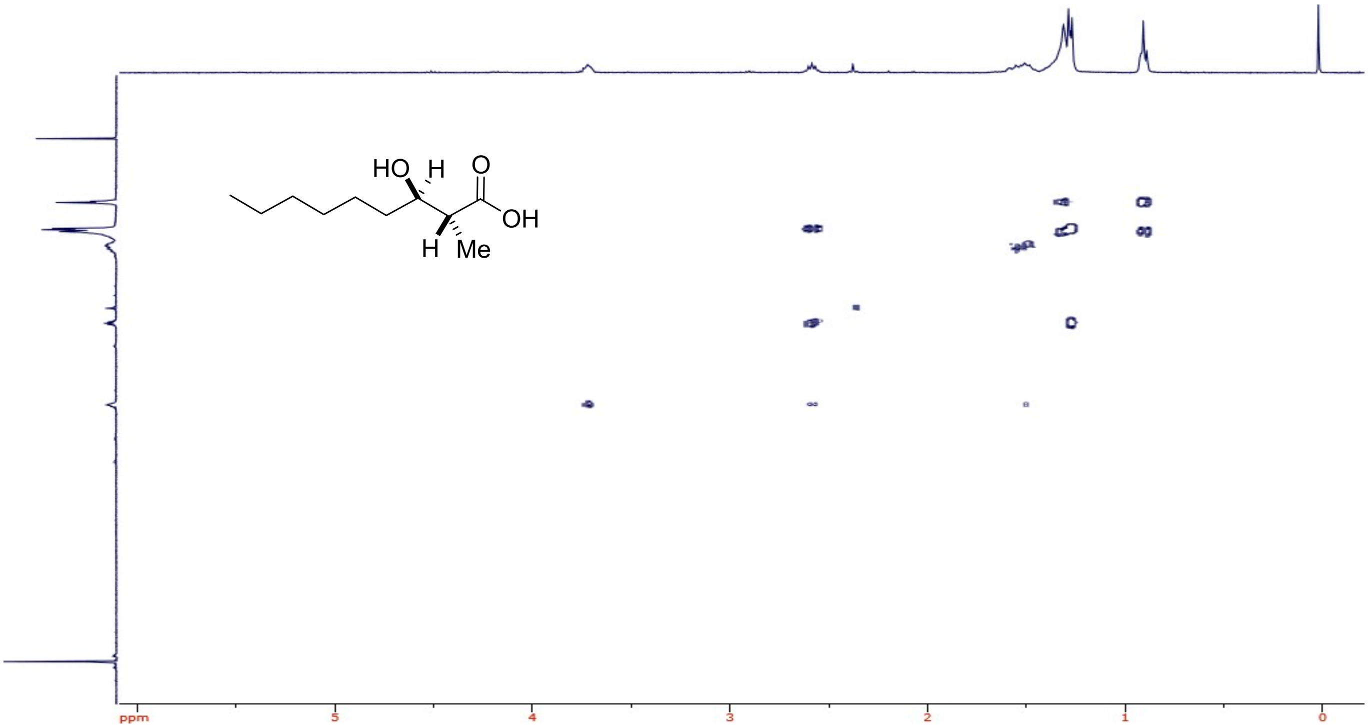

Figure S19: COSY spectrum ($CDCl_3$) of acid **2**

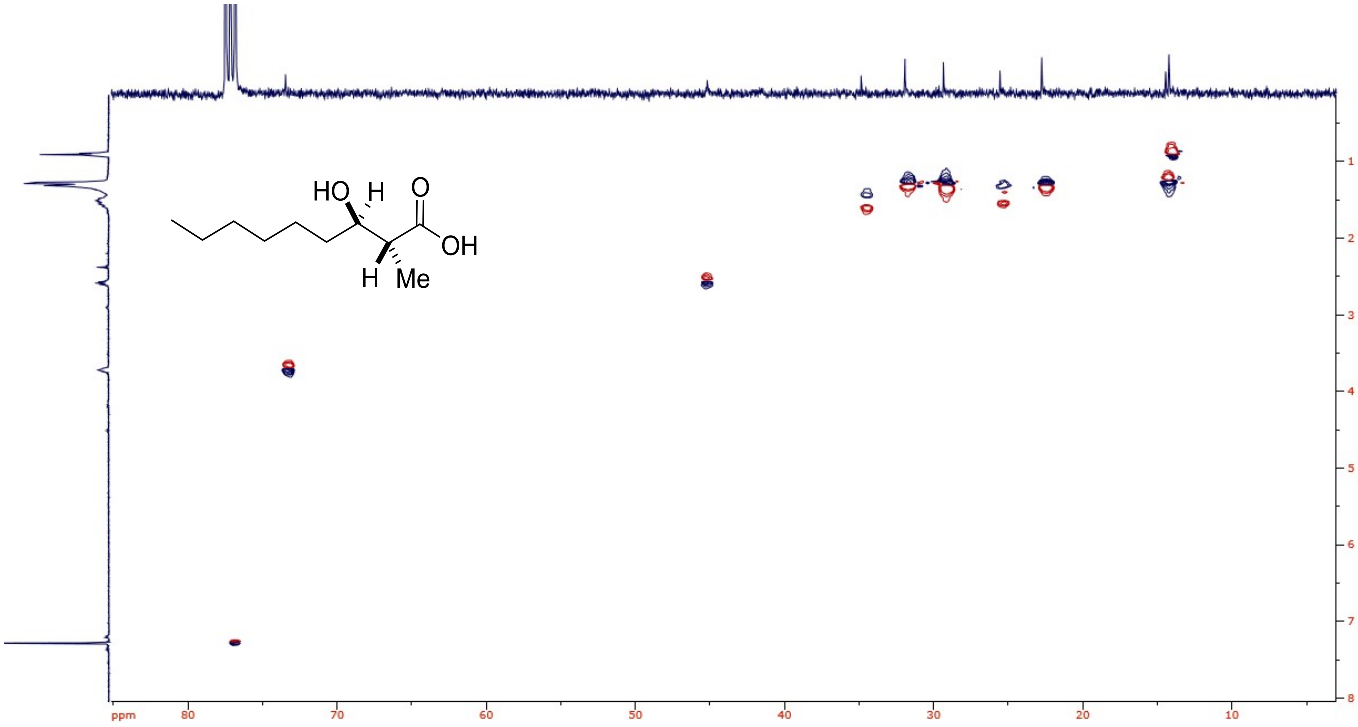

Figure S20: HSQC spectrum ($CDCl_3$) of acid **2**

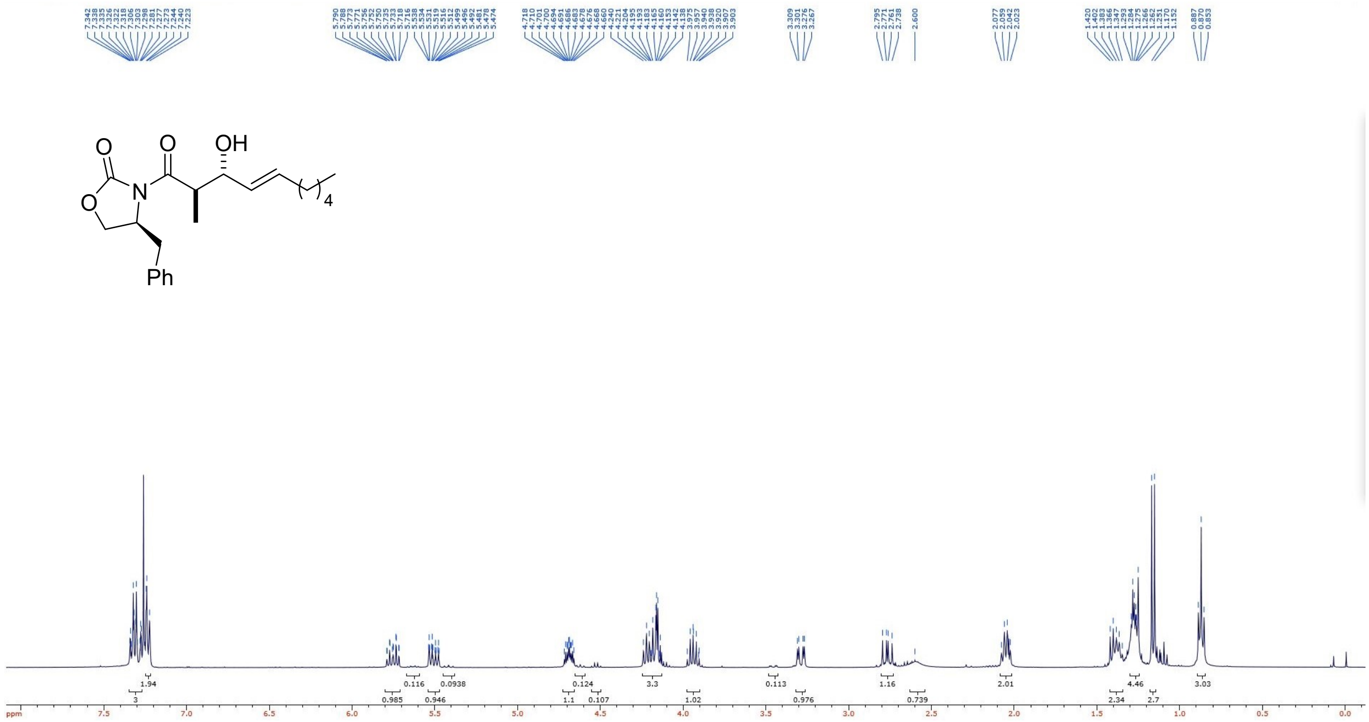

Figure S21: ^{1}H NMR spectrum (400 MHz, $CDCl_3$) of alkene **10**

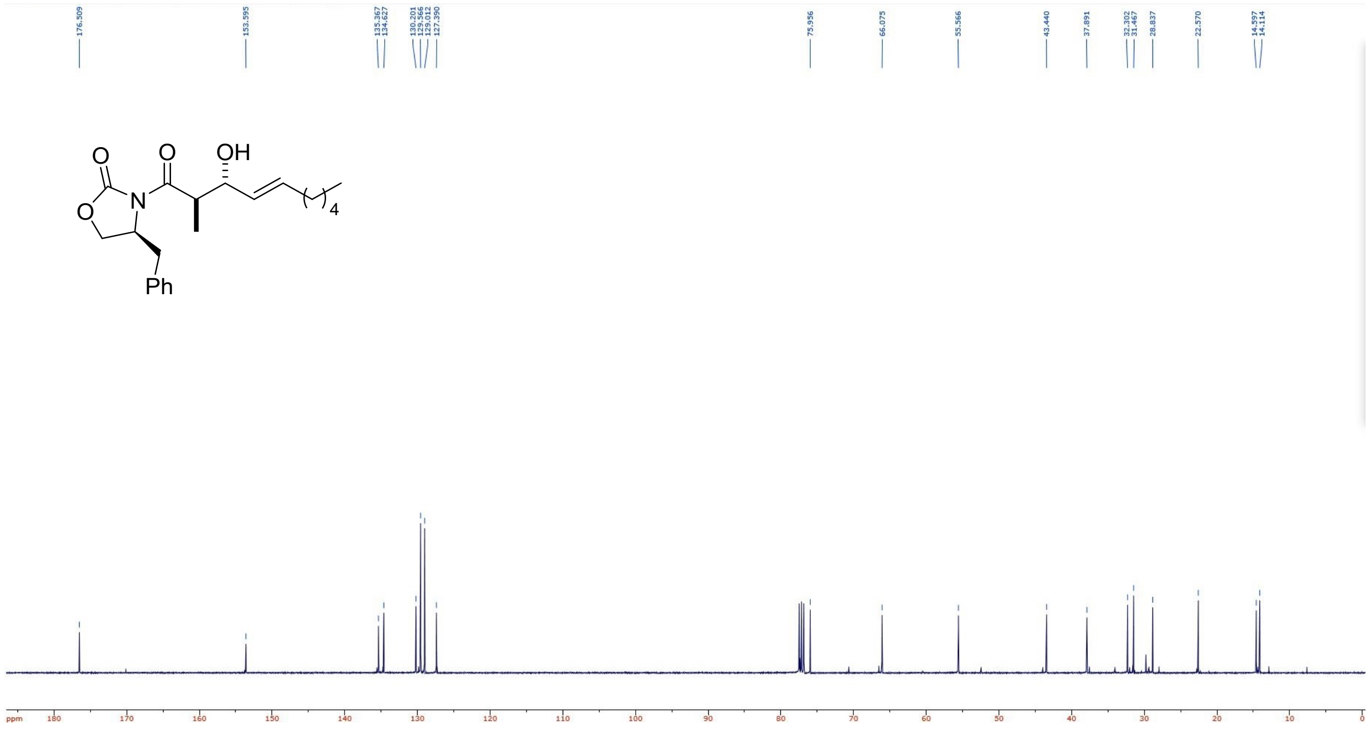

Figure S22: ^{13}C NMR spectrum (101 MHz, $CDCl_3$) of alkene **10**

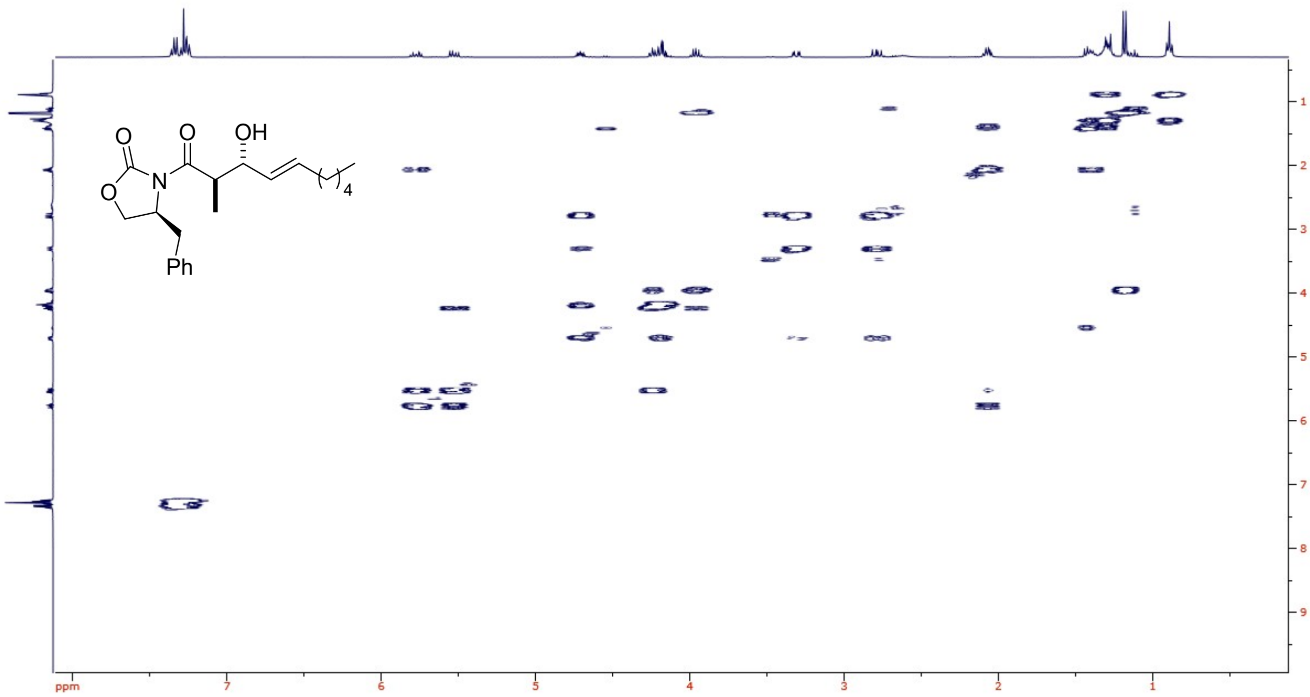

Figure S23: COSY spectrum ($CDCl_3$) of alkene **10**

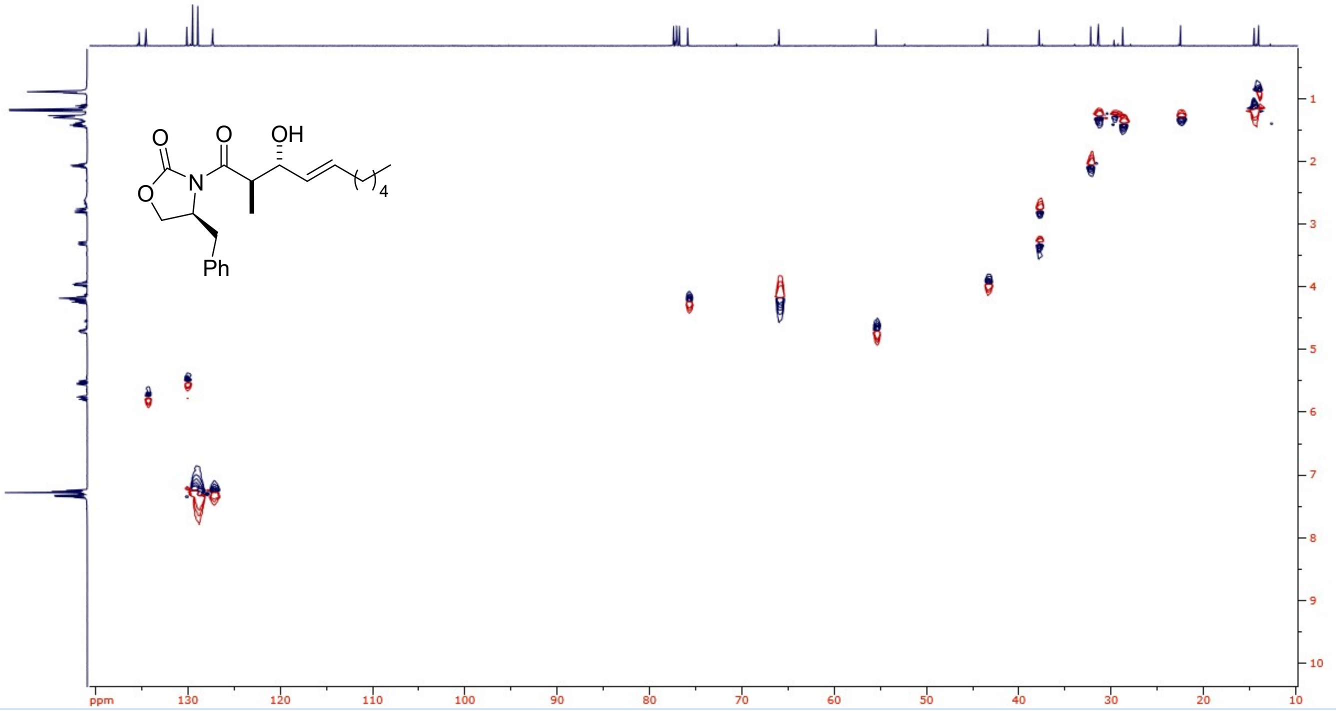

Figure S24: HSQC spectrum ($CDCl_3$) of alkene **10**

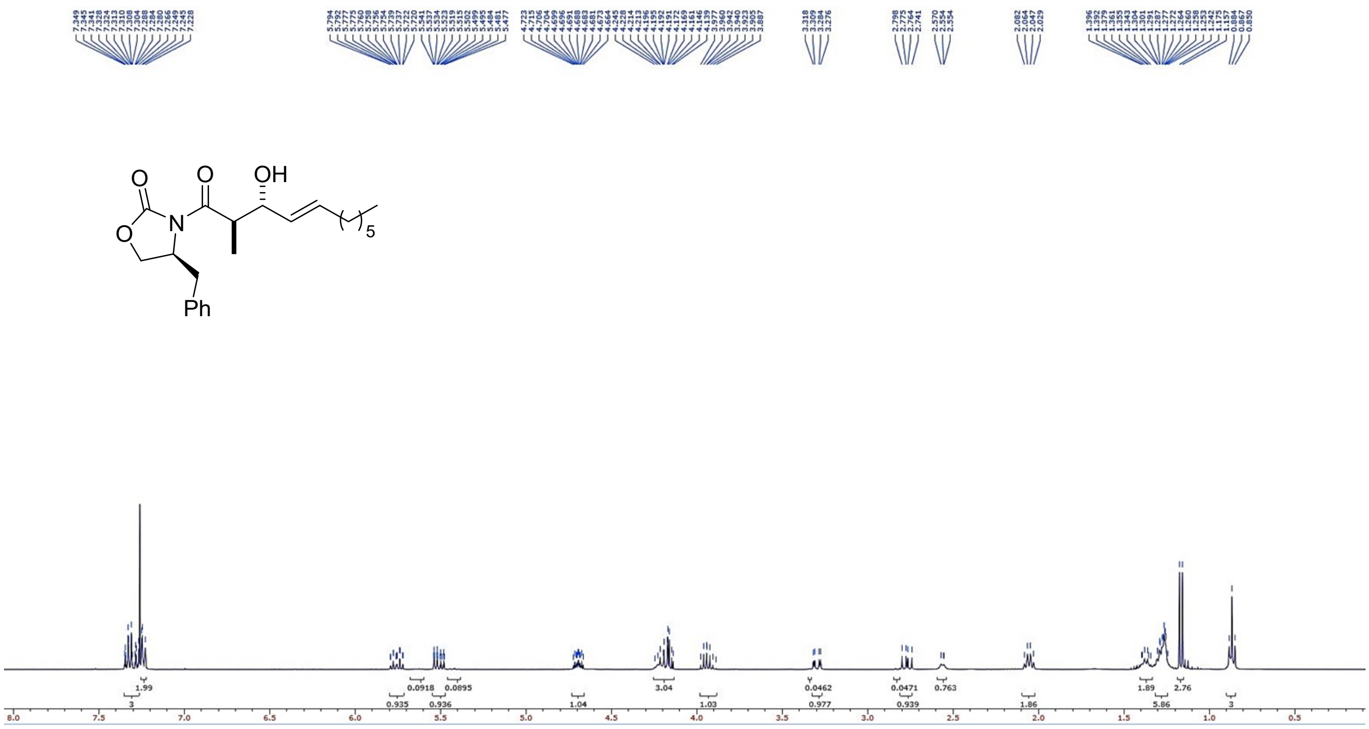

Figure S25: ^{1}H NMR spectrum (400 MHz, $CDCl_3$) of alkene **11**

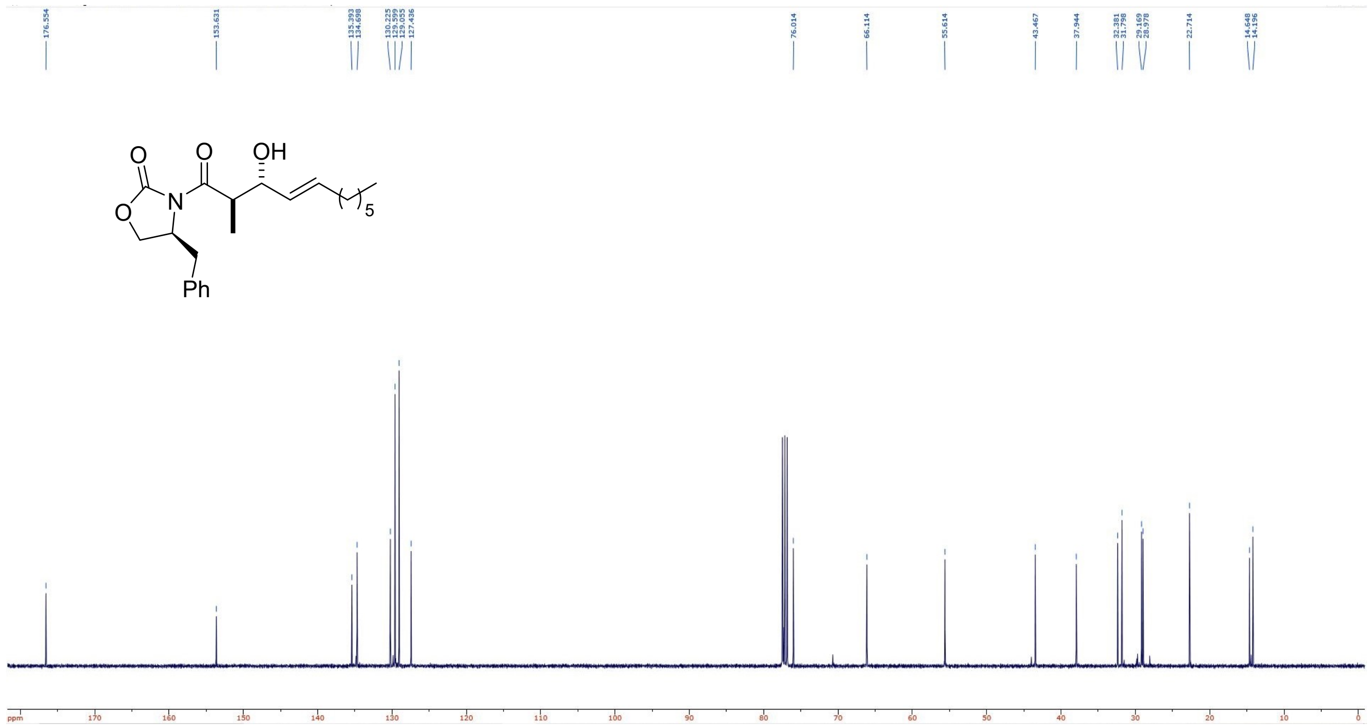

Figure S26: ^{13}C NMR spectrum (101 MHz, $CDCl_3$) of alkene **11**

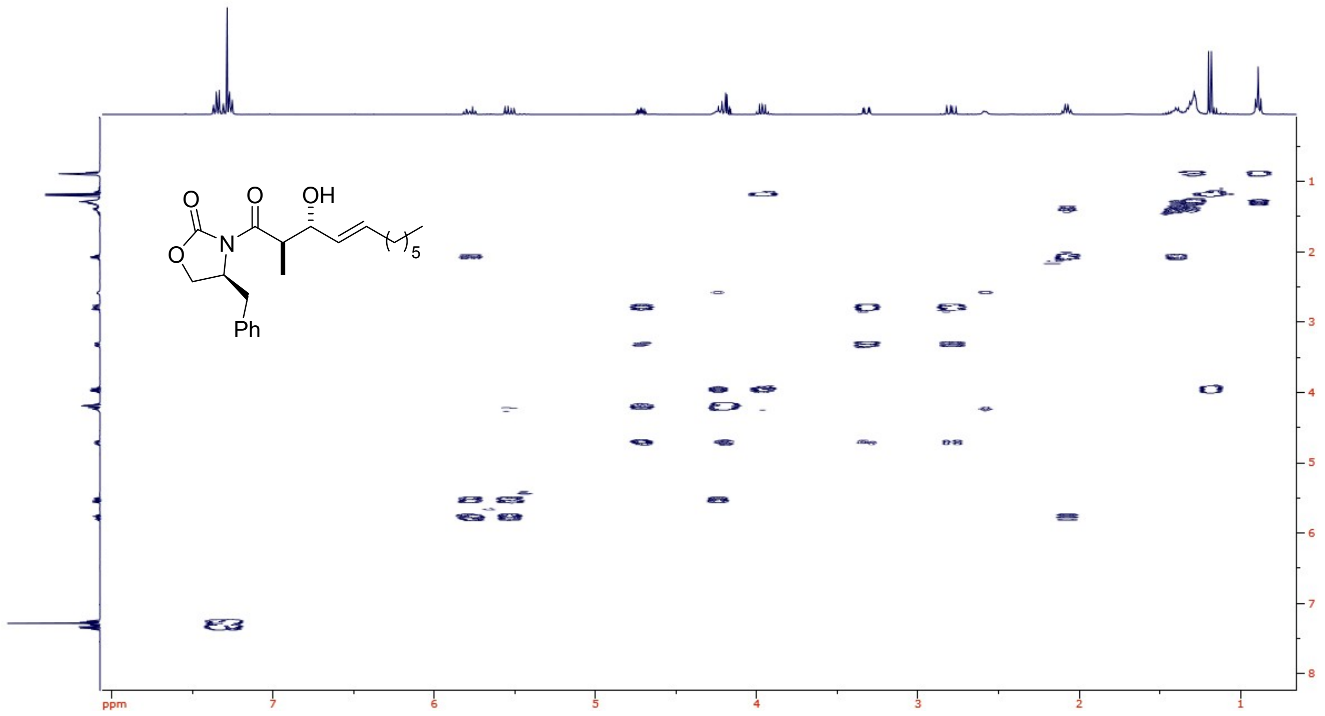

Figure S27: COSY spectrum ($CDCl_3$) of alkene **11**

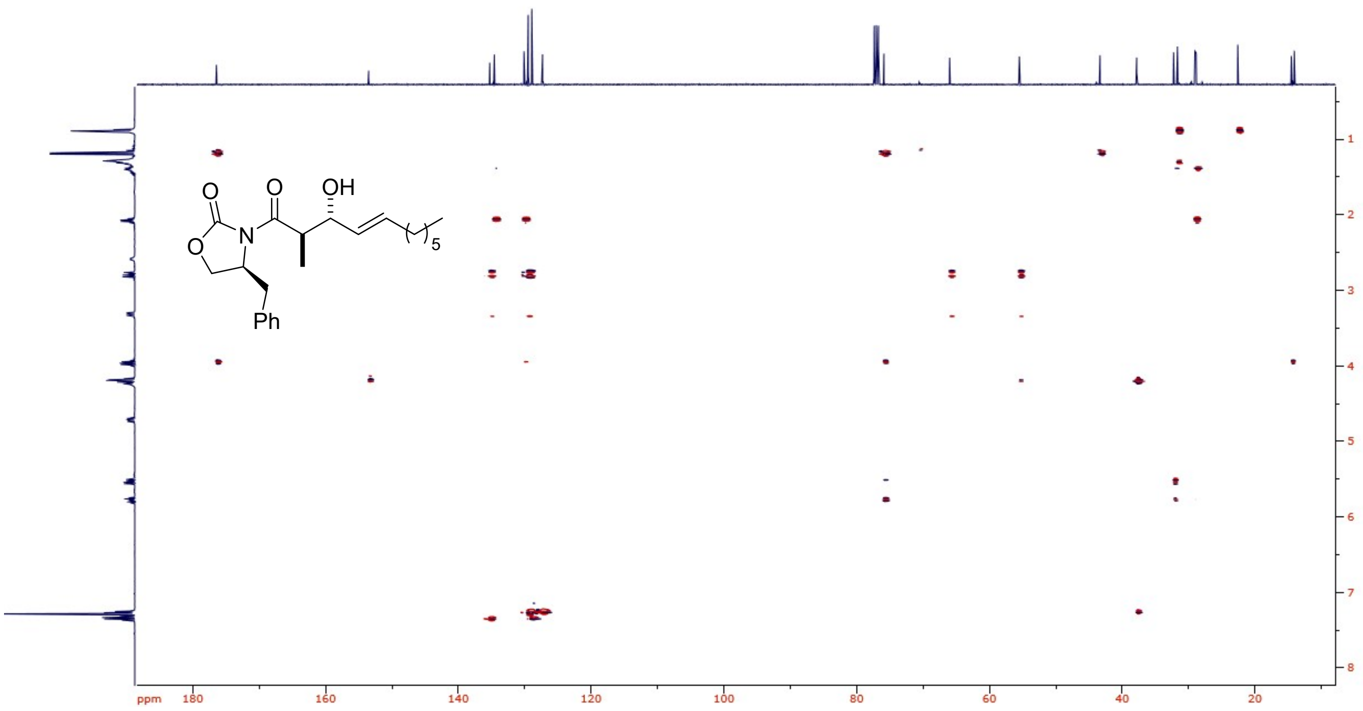

Figure S28: HMBC spectrum ($CDCl_3$) of alkene **11**

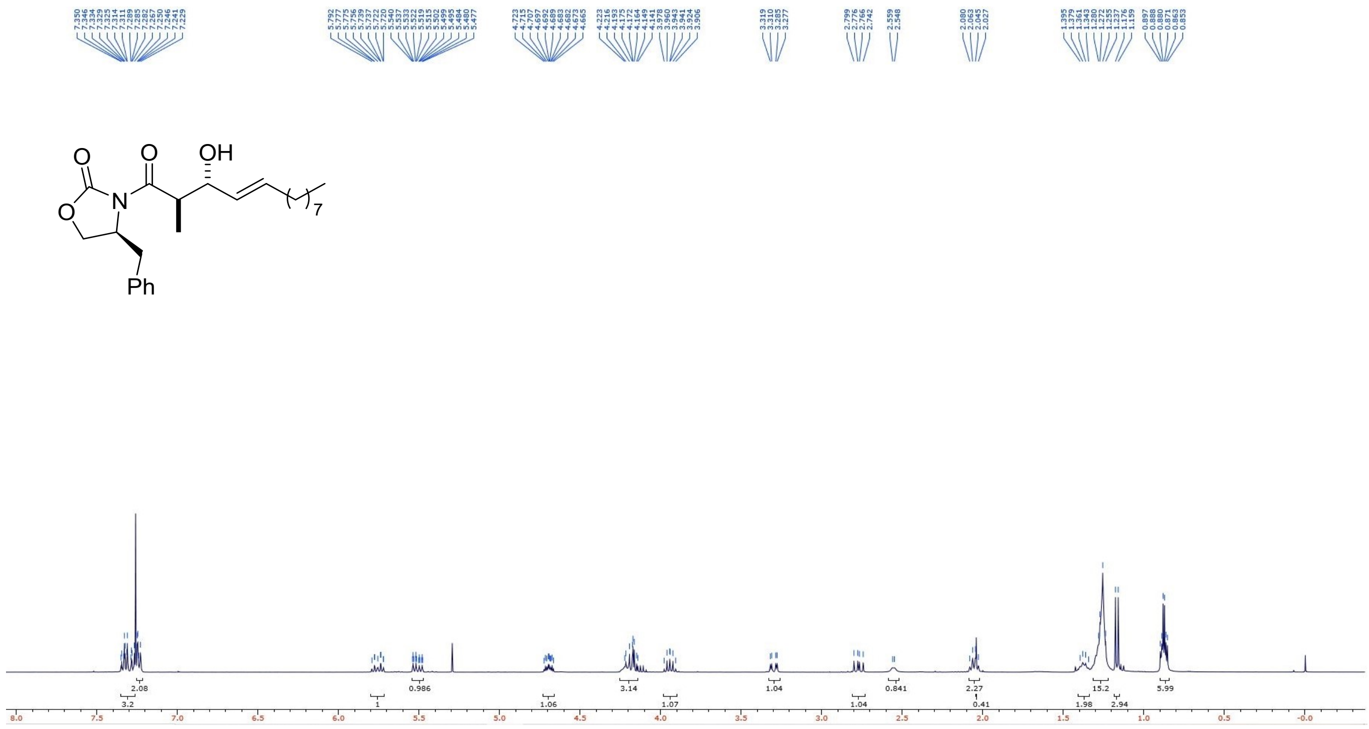

Figure S29: ^{1}H NMR spectrum (400 MHz, $CDCl_3$) of alkene **12**

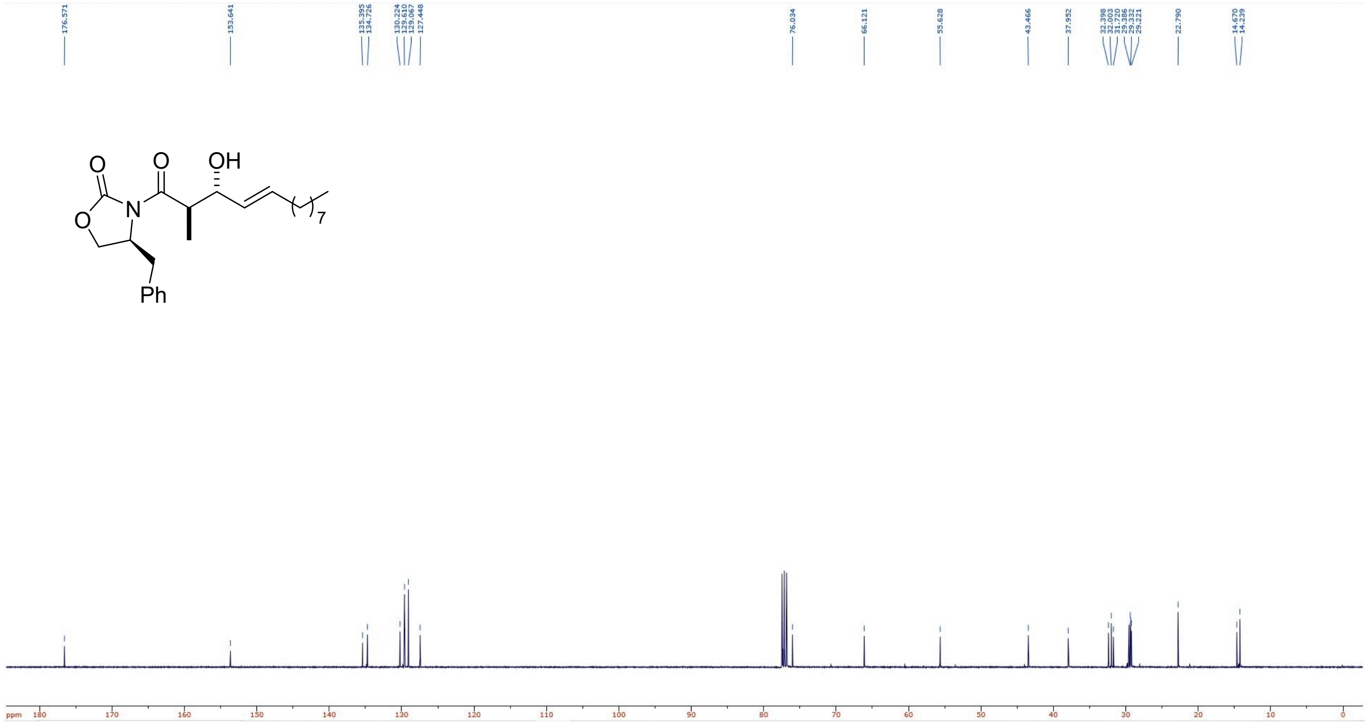

Figure S30: ^{13}C NMR spectrum (101 MHz, $CDCl_3$) of alkene **12**

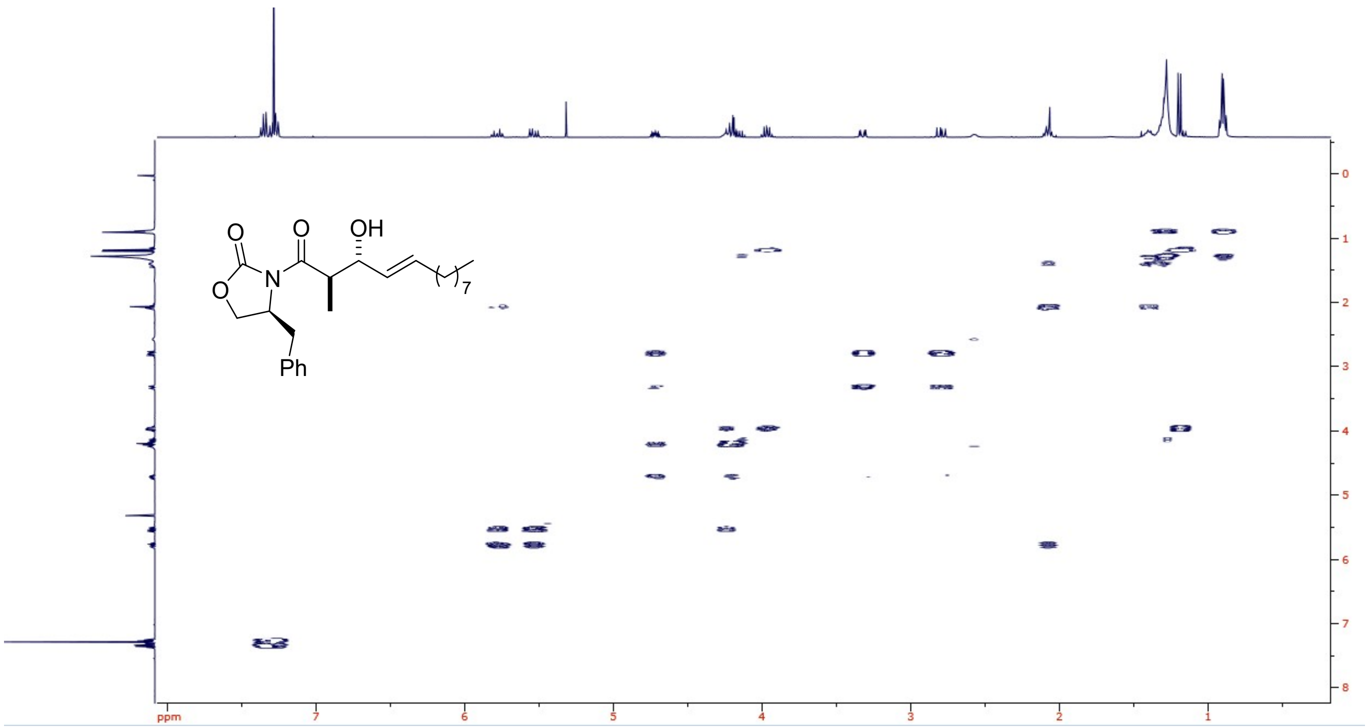

Figure S31: COSY spectrum ($CDCl_3$) of alkene **12**

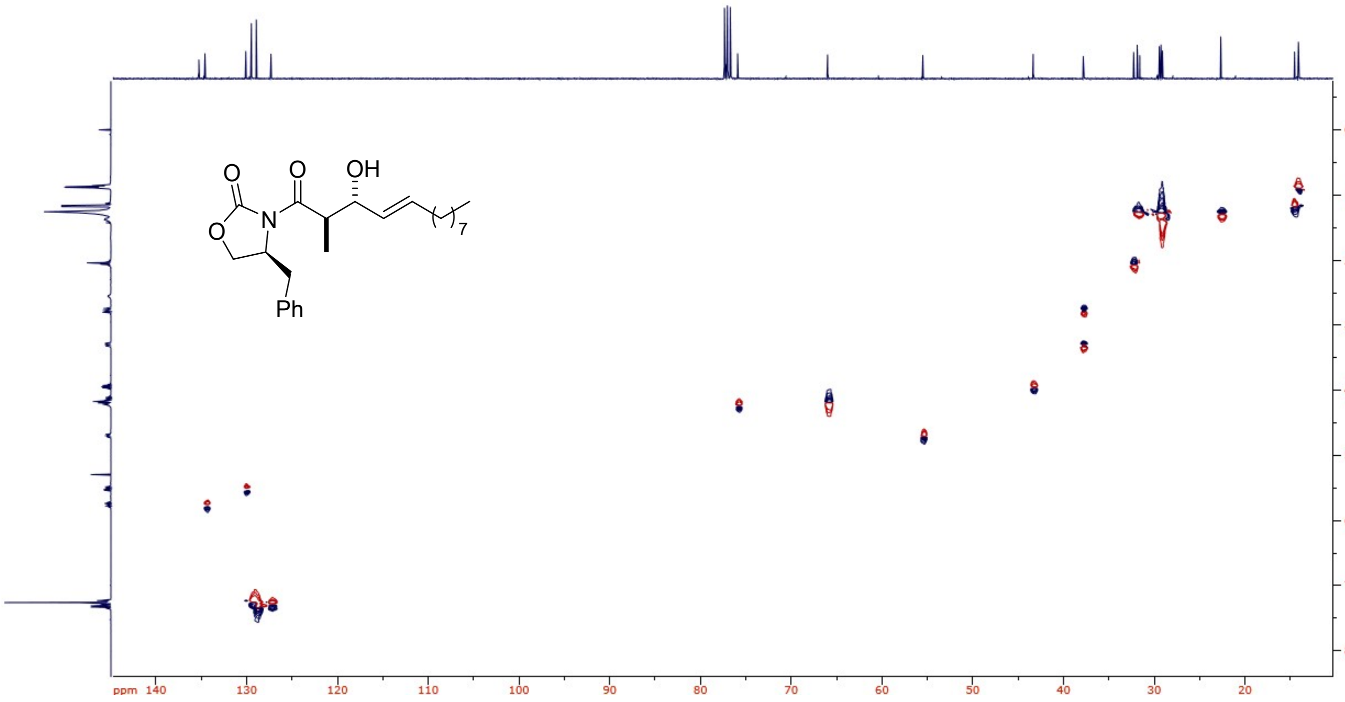

Figure S32: HSQC spectrum ($CDCl_3$) of alkene **12**

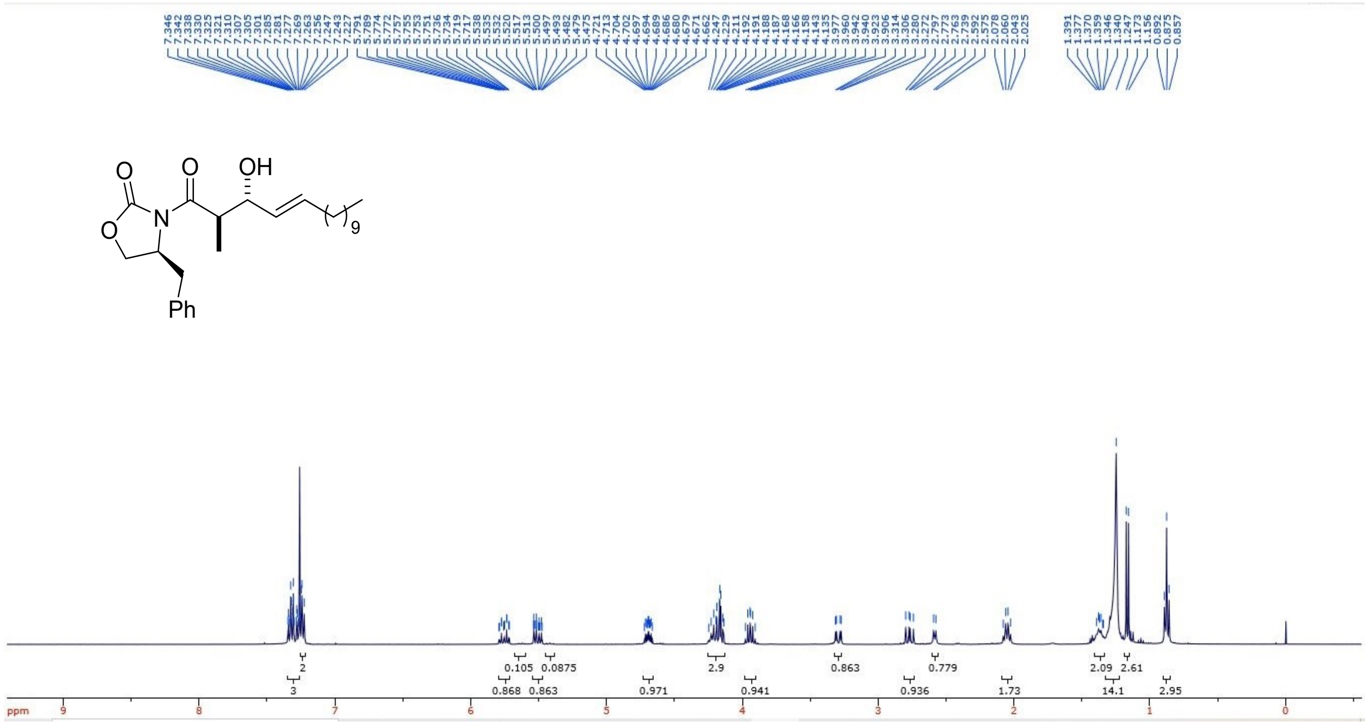

Figure S33: ^{1}H NMR spectrum (400 MHz, $CDCl_3$) of alkene **13**

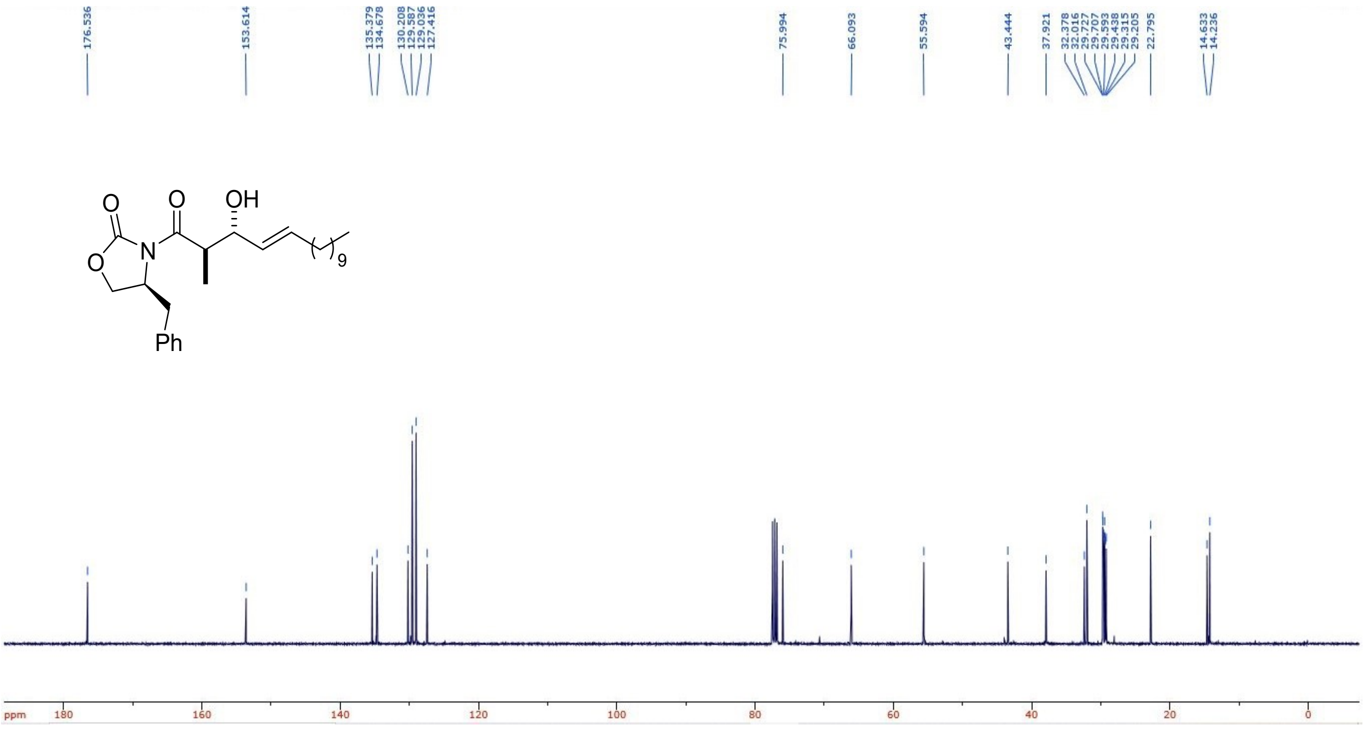

Figure S34: ^{13}C NMR spectrum (101 MHz, $CDCl_3$) of alkene **13**

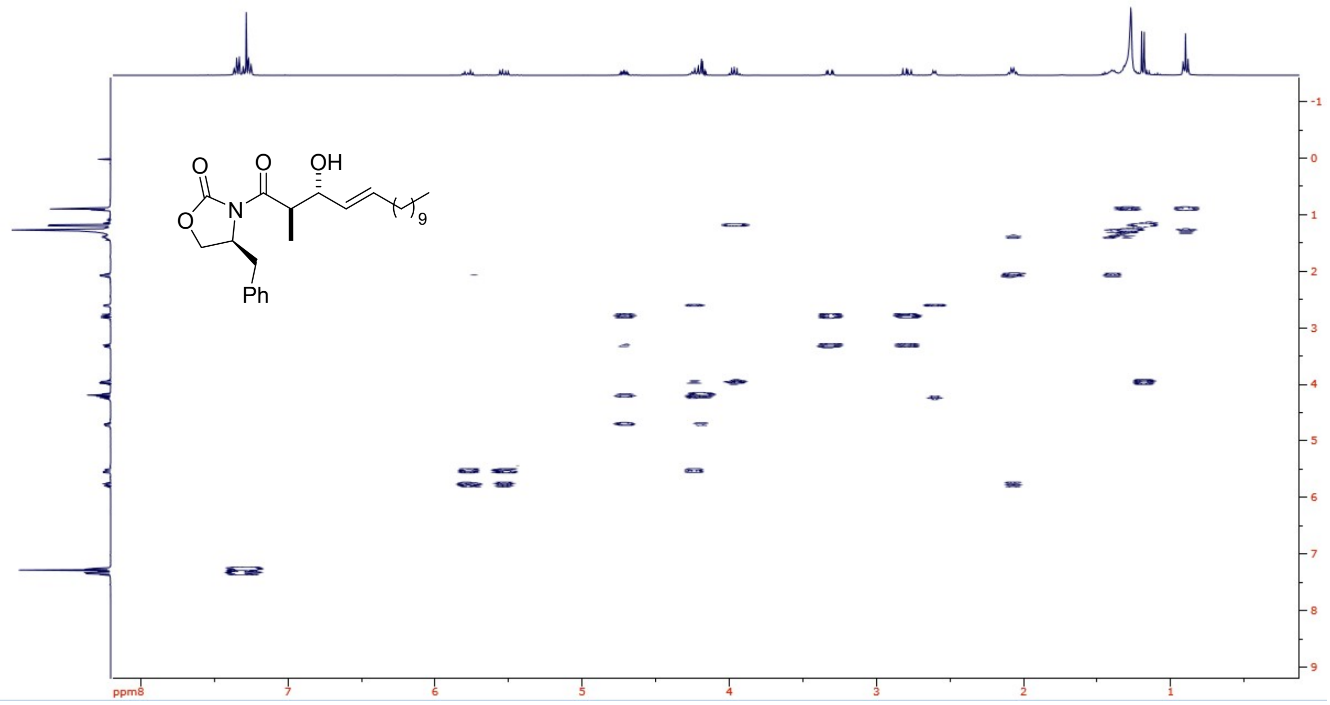

Figure S35: COSY spectrum ($CDCl_3$) of alkene **13**

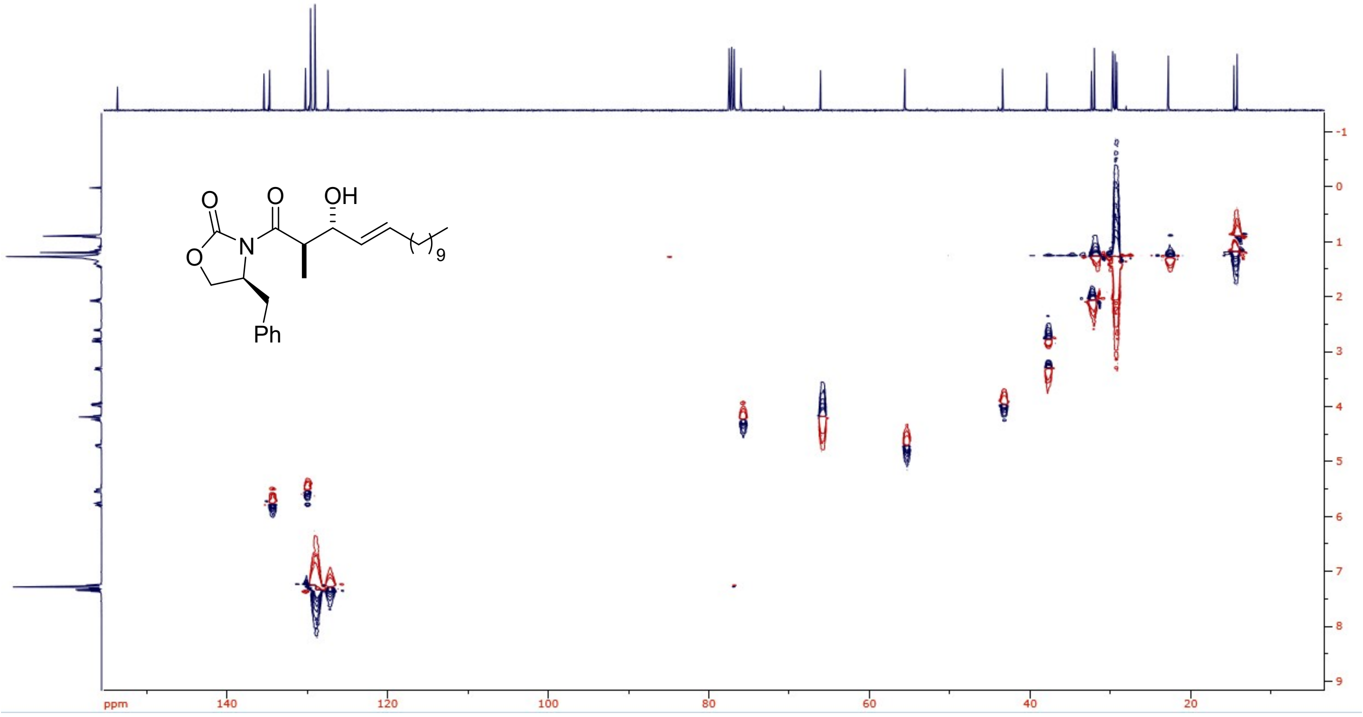

Figure S36: HSQC spectrum ($CDCl_3$) of alkene **13**

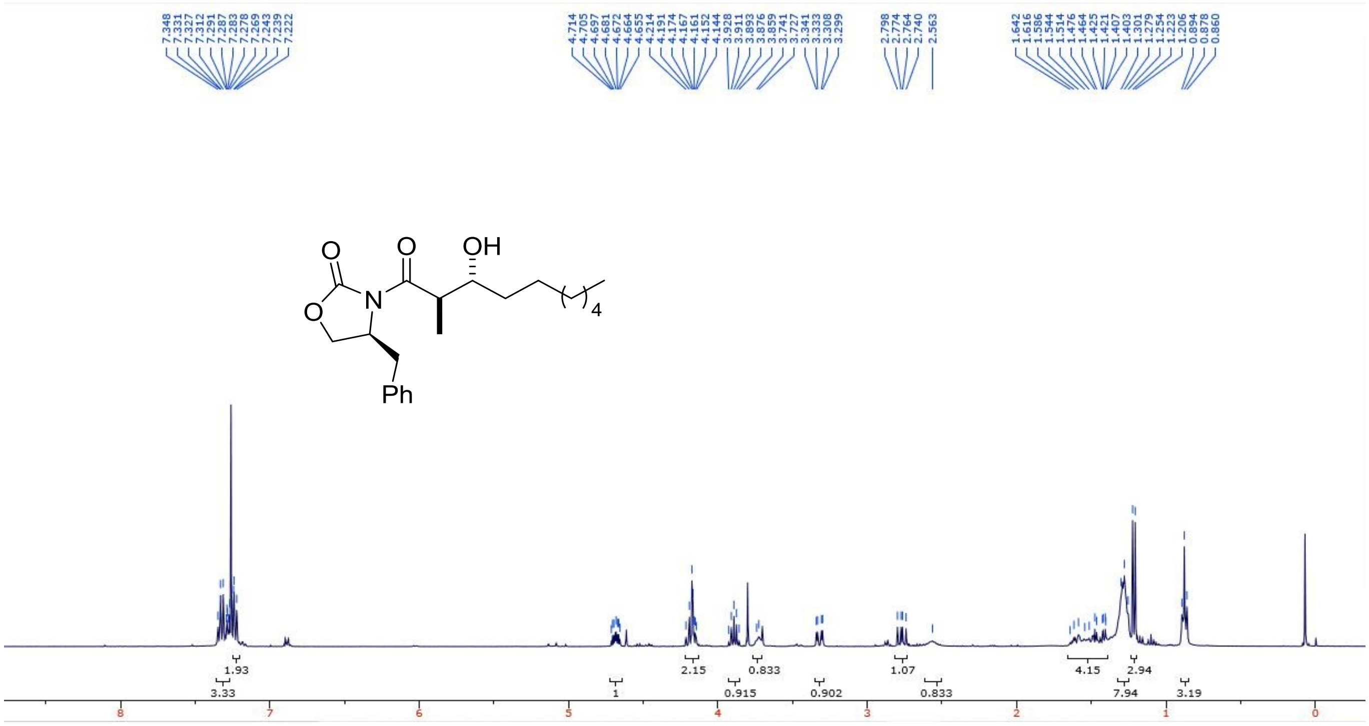

Figure S37: ^{1}H NMR spectrum (400 MHz, $CDCl_3$) of alkane **14**

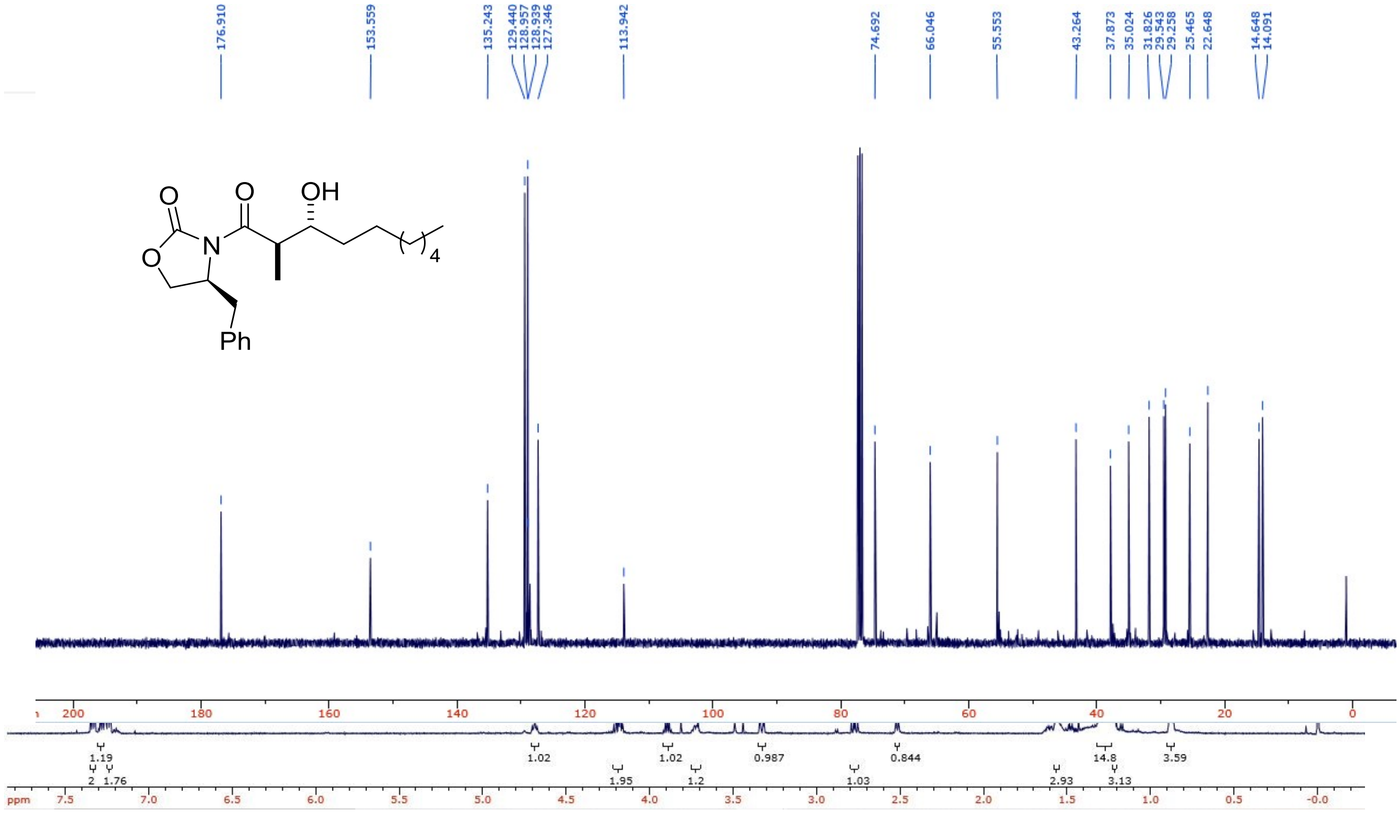

Figure S38: ^{13}C NMR spectrum (101 MHz, $CDCl_3$) of alkane **14**

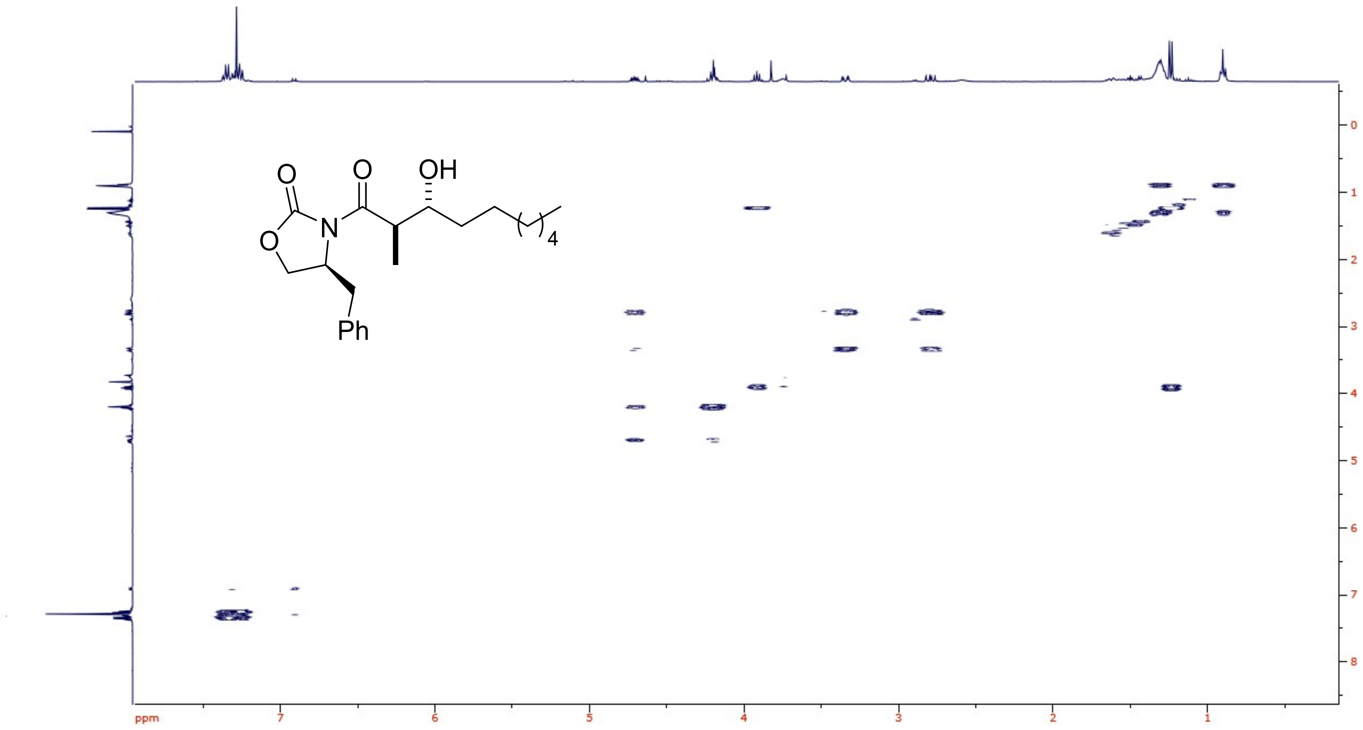

Figure S39: COSY spectrum ($CDCl_3$) of alkane **14**

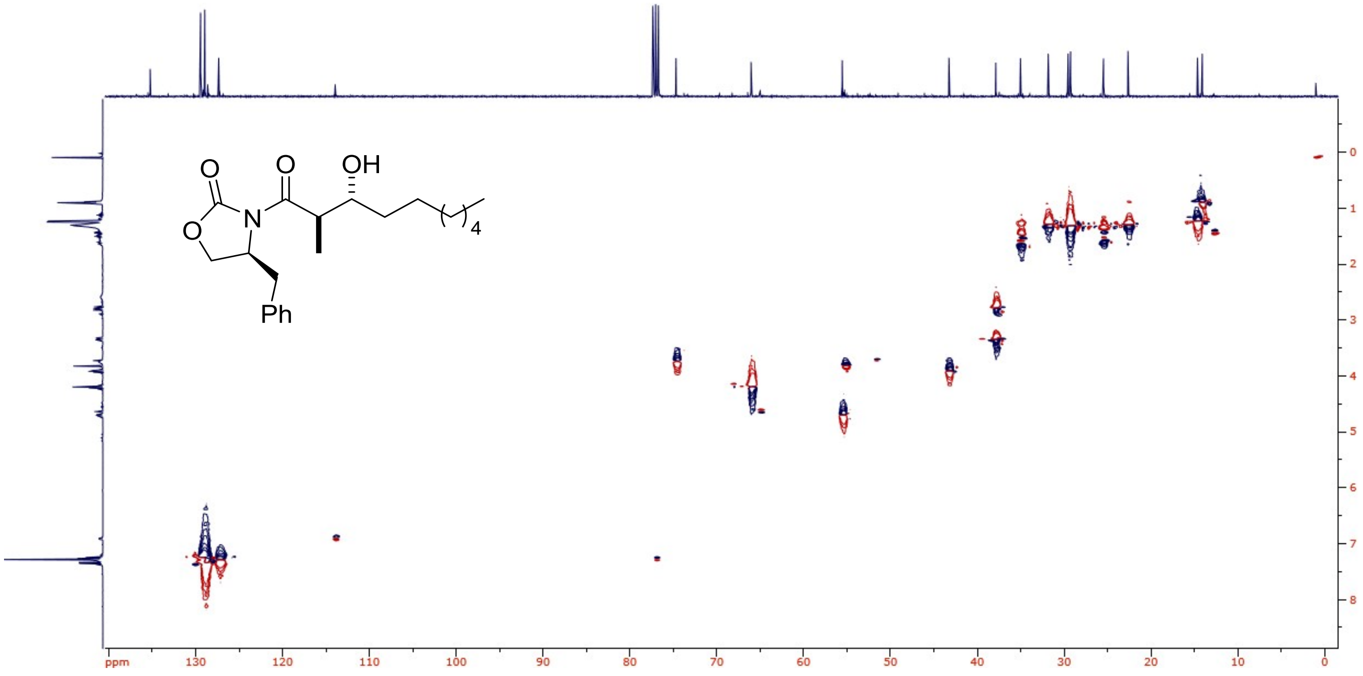

Figure S40: HSQC spectrum ($CDCl_3$) of alkane **14**

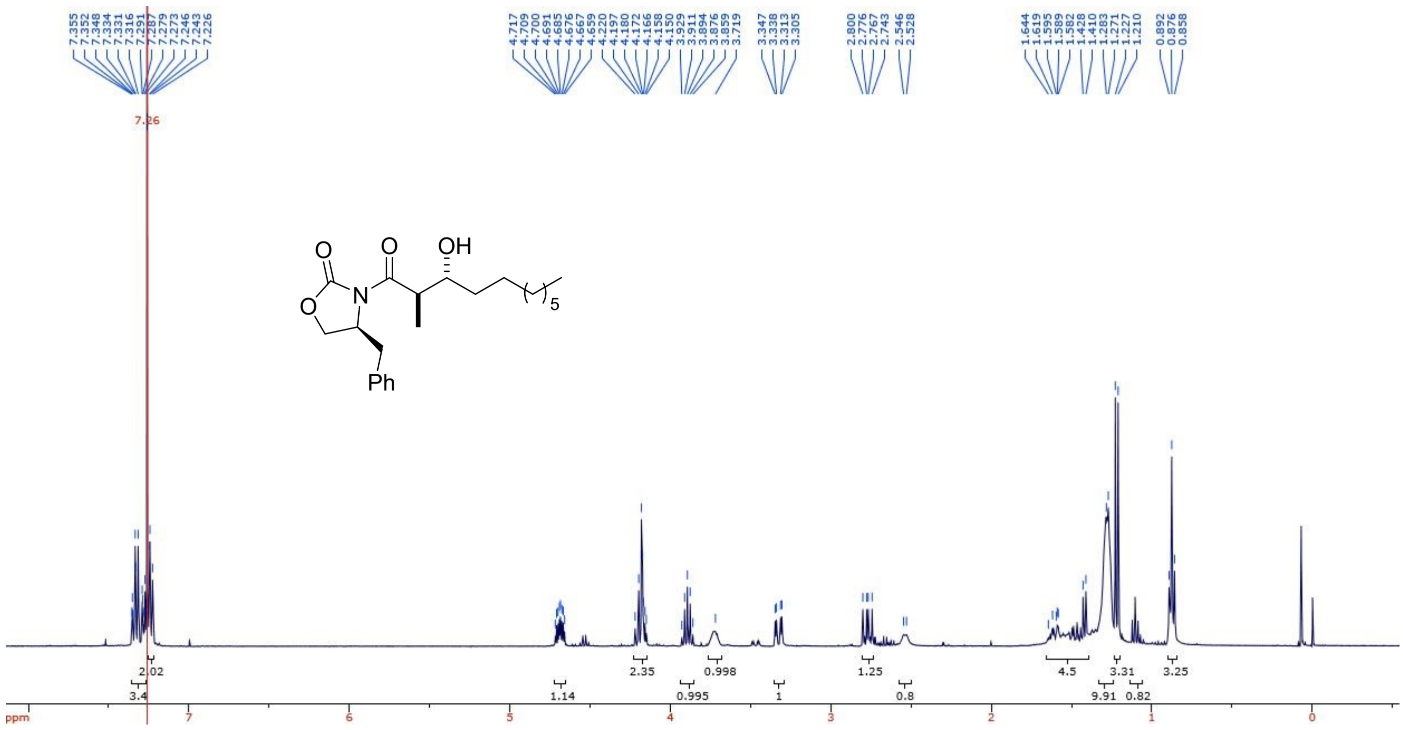

Figure S41: ^{1}H NMR spectrum (400 MHz, $CDCl_3$) of alkane **15**

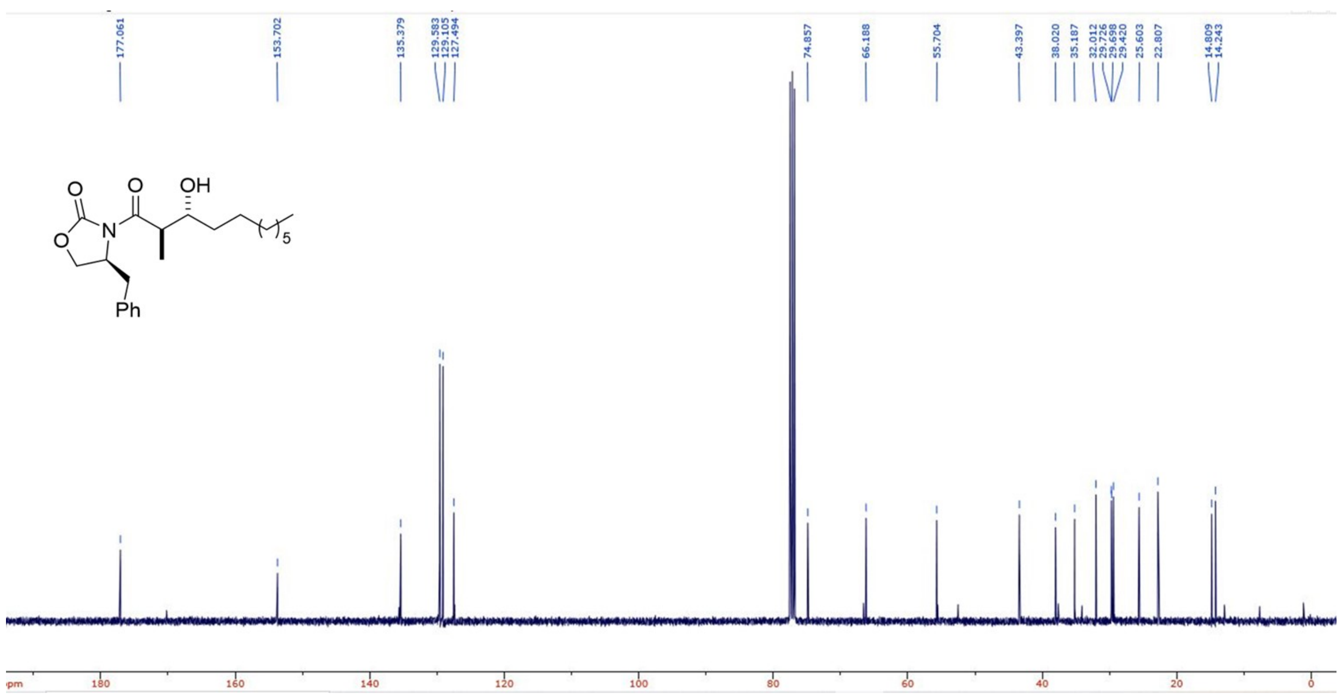

Figure S42: ^{13}C NMR spectrum (101 MHz, $CDCl_3$) of alkane **15**

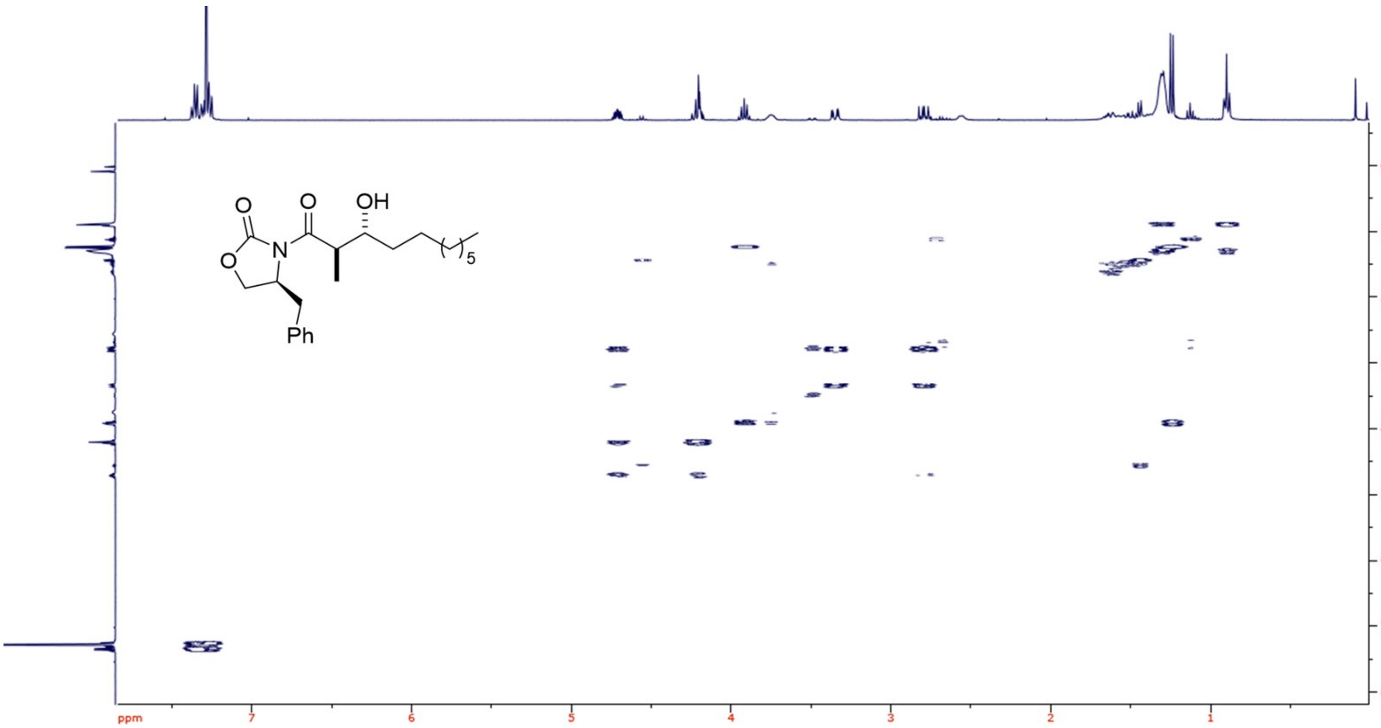

Figure S43: COSY spectrum ($CDCl_3$) of alkane **15**

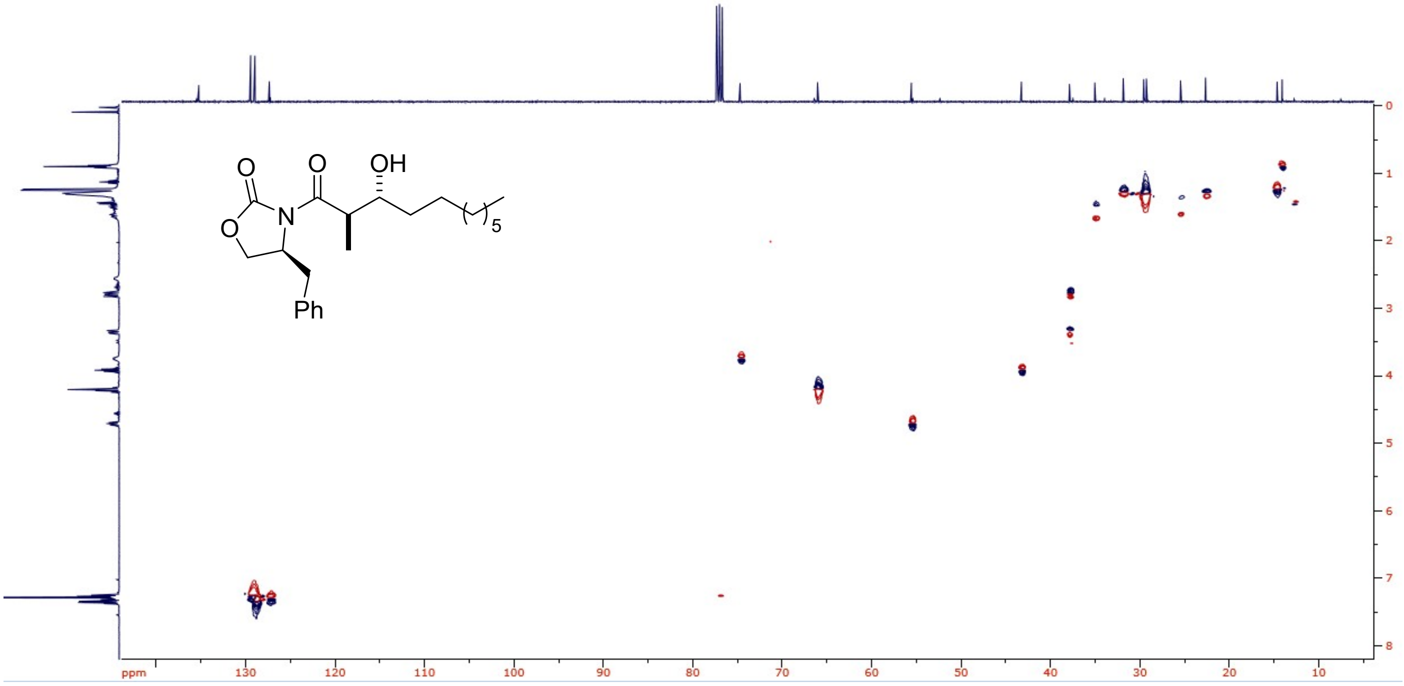

Figure S44: HSQC spectrum ($CDCl_3$) of alkane **15**

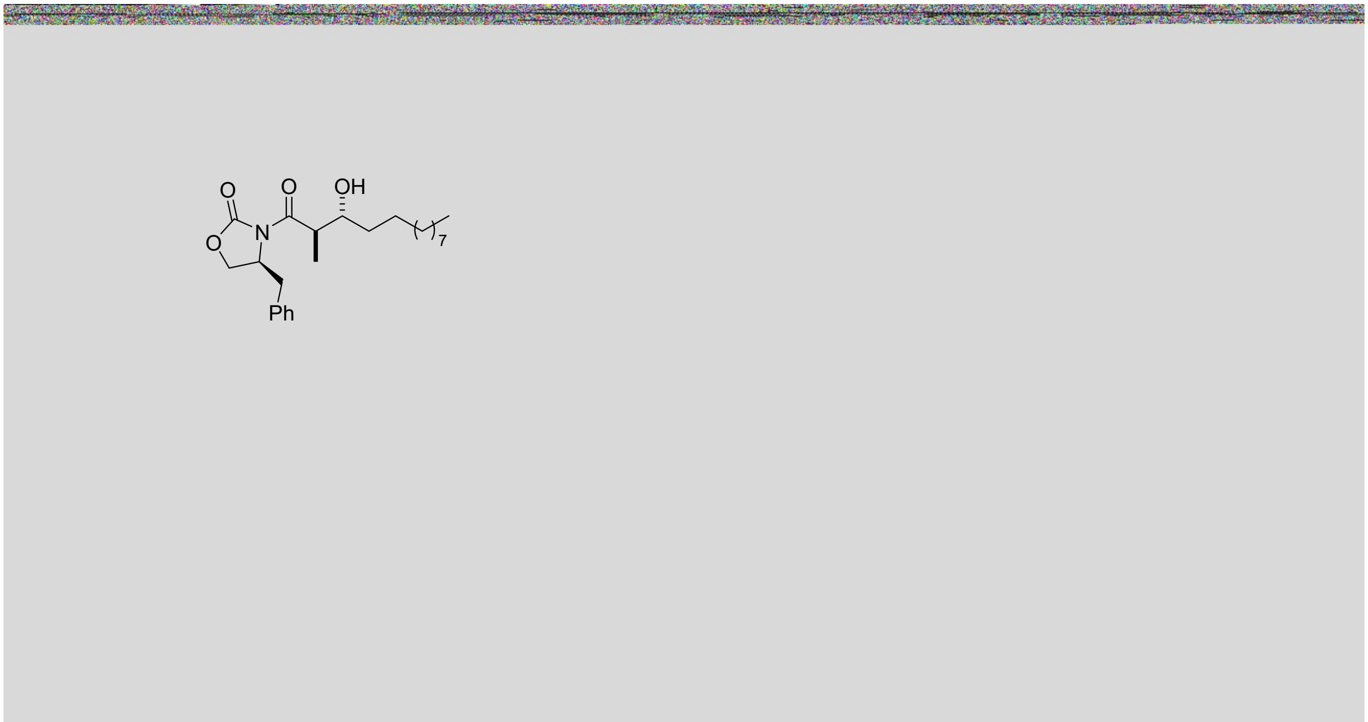

Figure S45: ^{1}H NMR spectrum (400 MHz, $CDCl_3$) of alkane **16**

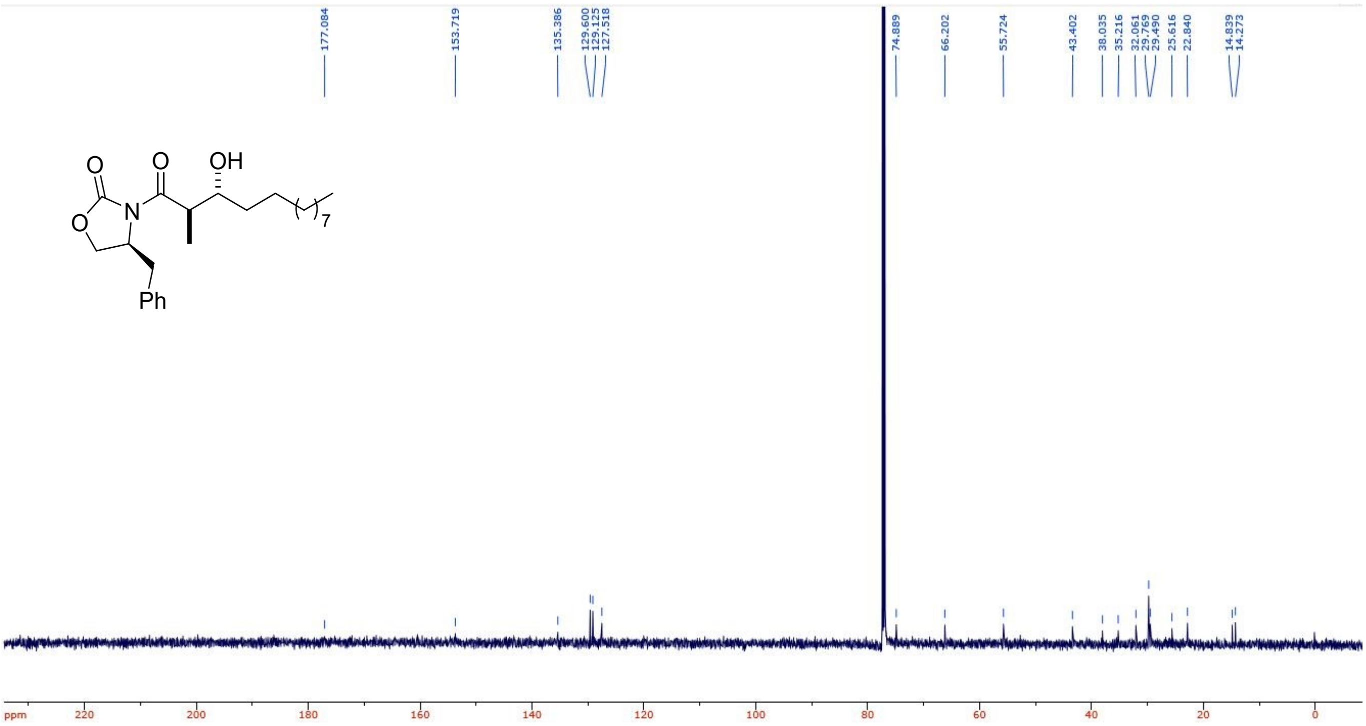

Figure S46: ^{13}C NMR spectrum (101 MHz, $CDCl_3$) of alkane **16**

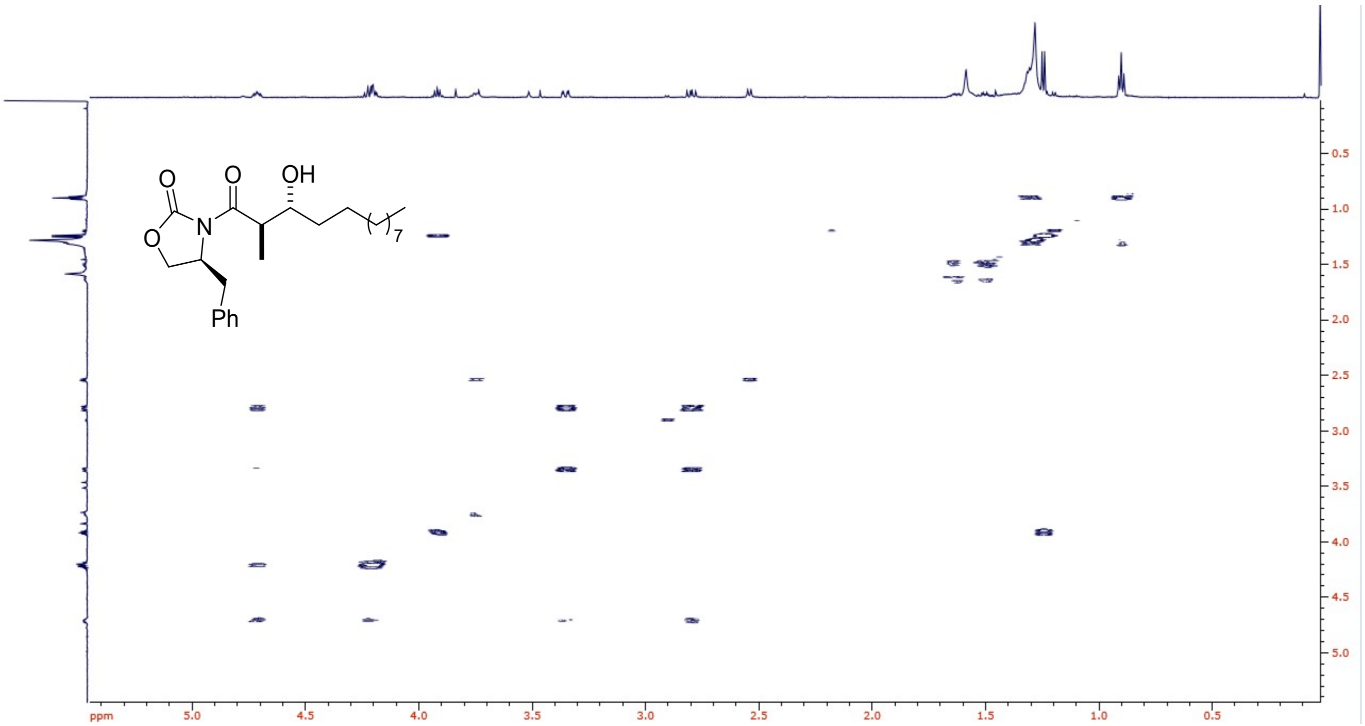

Figure S47: COSY spectrum ($CDCl_3$) of alkane **16**

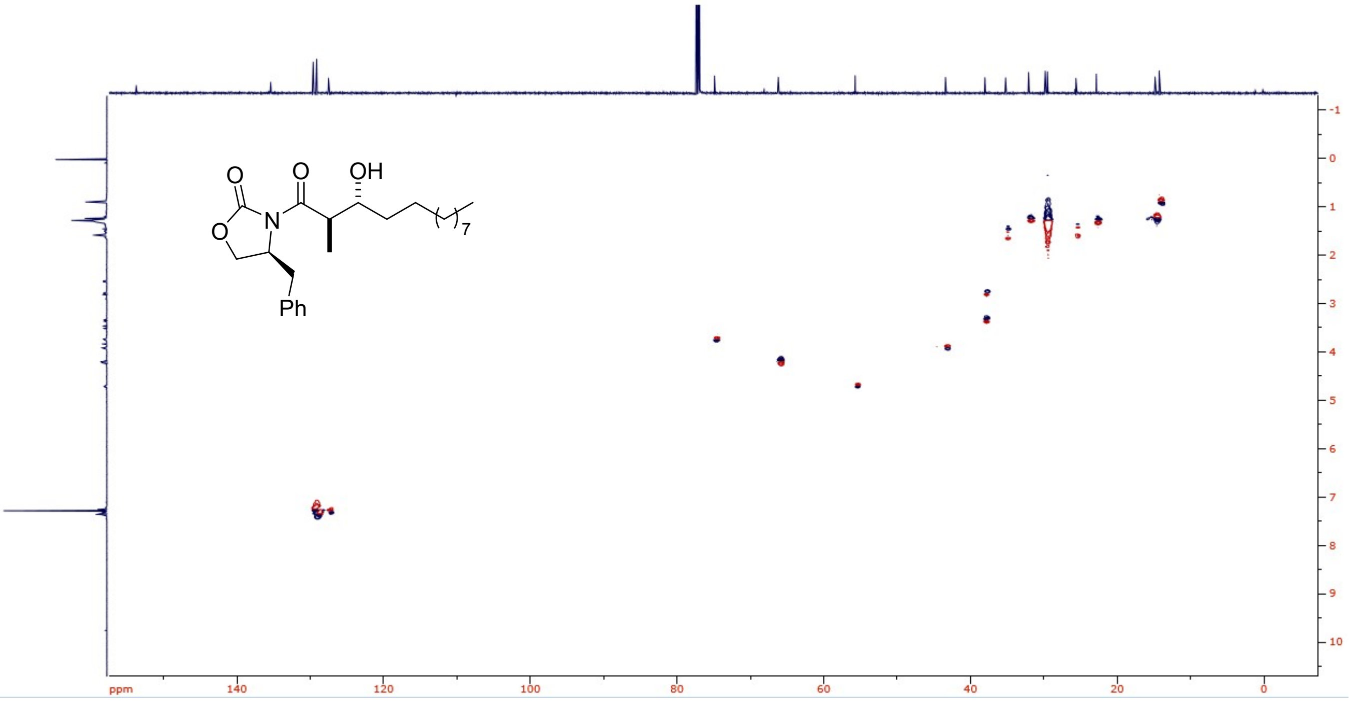

Figure S48: HSQC spectrum ($CDCl_3$) of alkane **16**

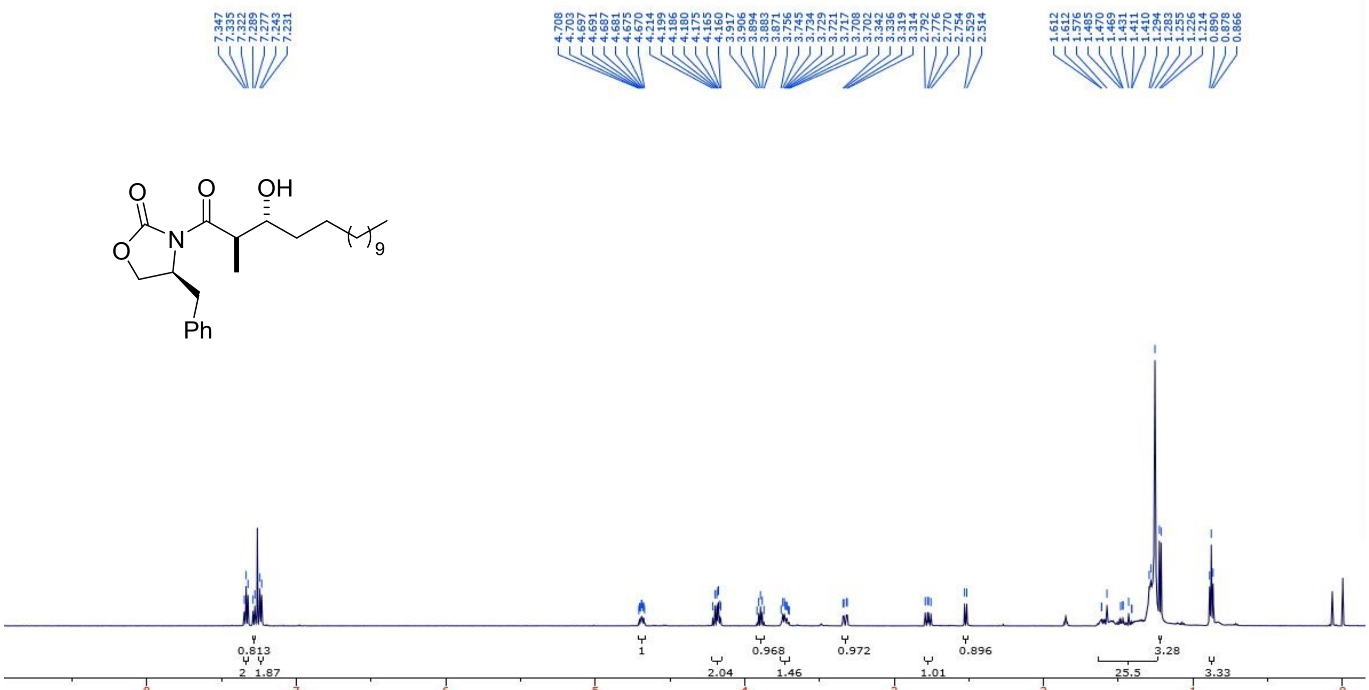

Figure S49: ^{1}H NMR spectrum (400 MHz, $CDCl_3$) of alkane **17**

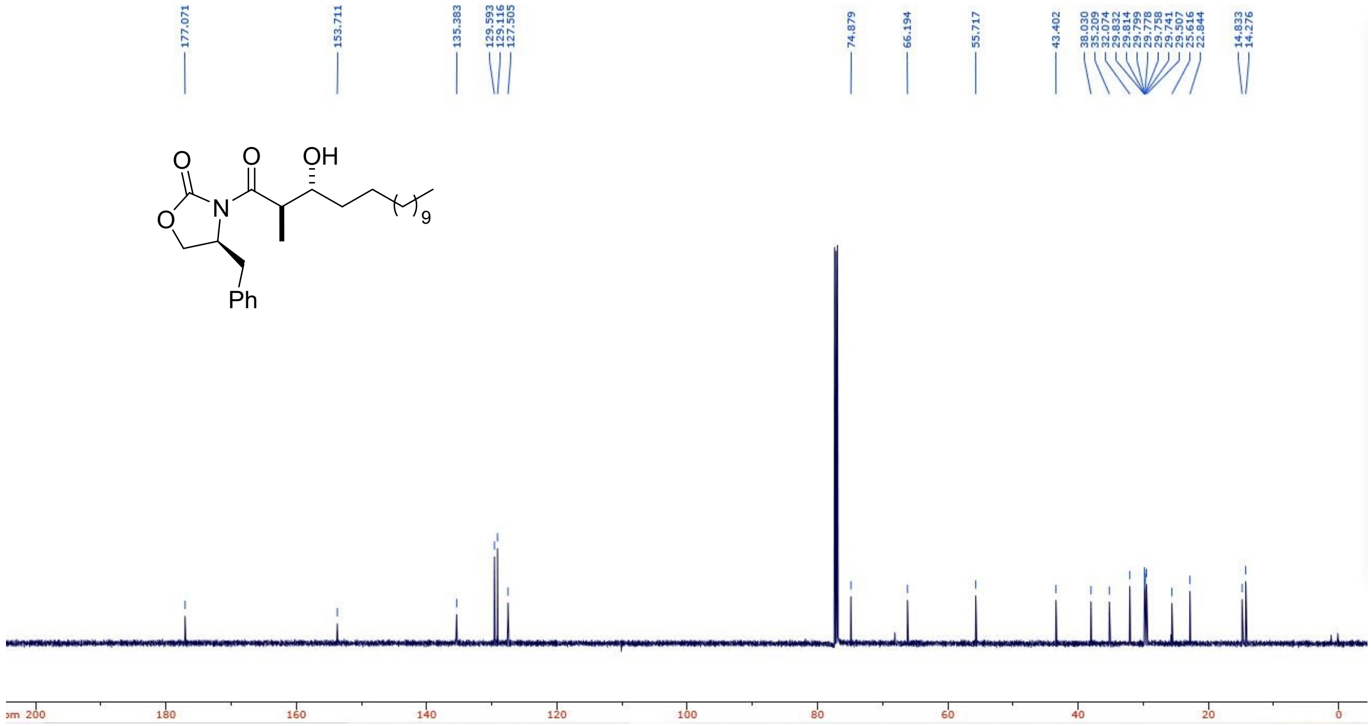

Figure S50: ^{13}C NMR spectrum (101 MHz, $CDCl_3$) of alkane **17**

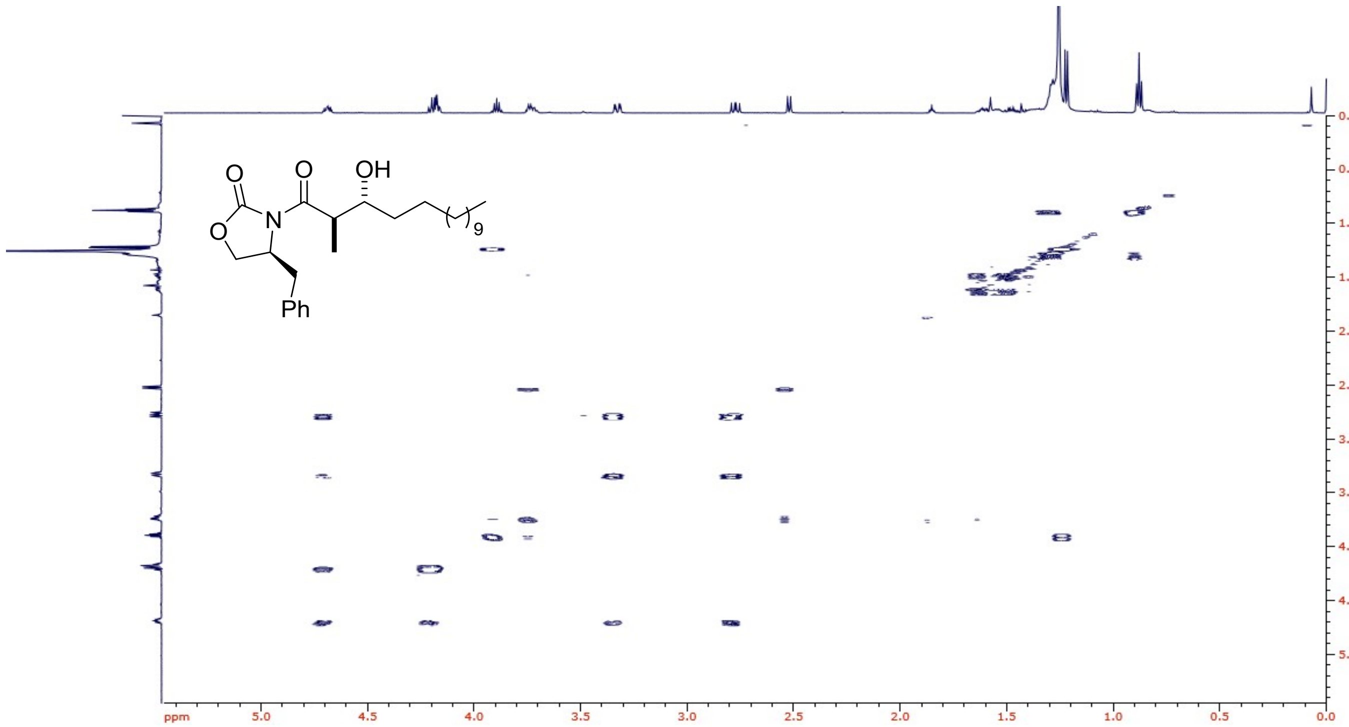

Figure S51: COSY spectrum ($CDCl_3$) of alkane **17**

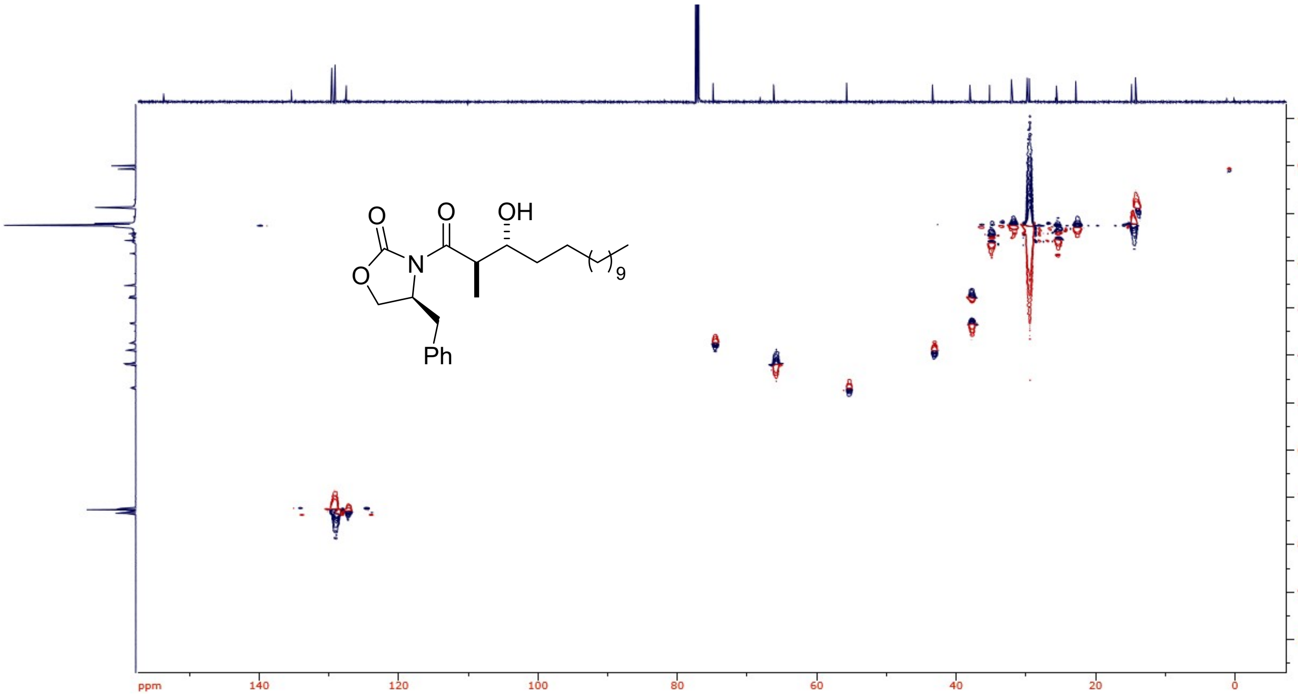

Figure S52: HSQC spectrum ($CDCl_3$) of alkane **17**

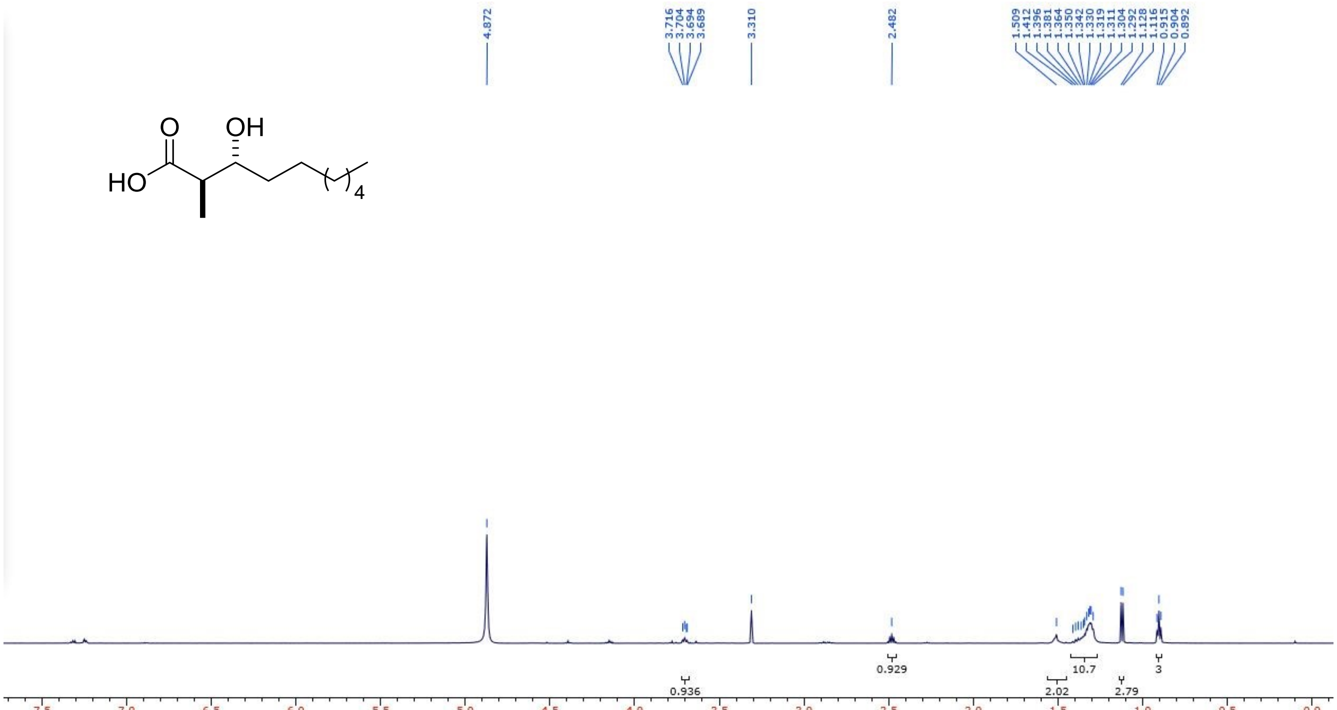

Figure S53: ^{1}H NMR spectrum (600 MHz, CD_3OD) of acid **18**

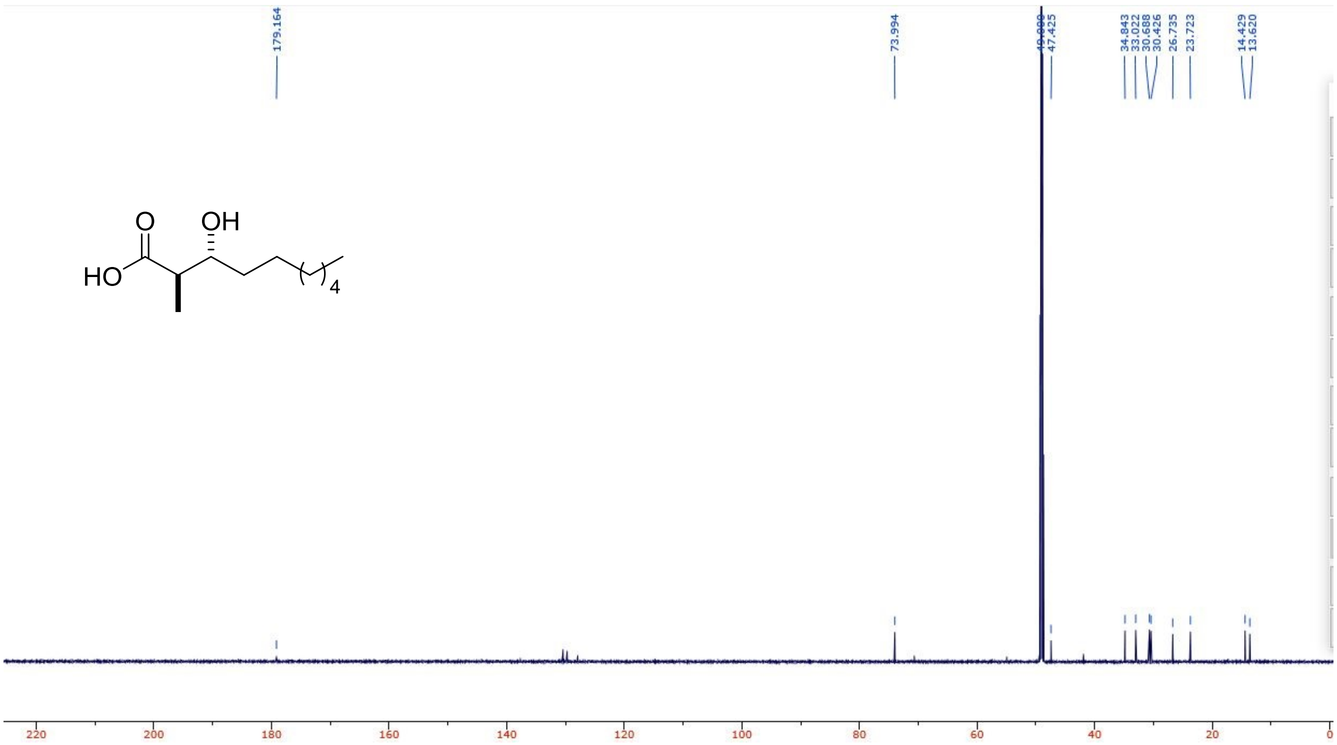

Figure S54: ^{13}C NMR spectrum (151 MHz, CD_3OD) of acid **18**

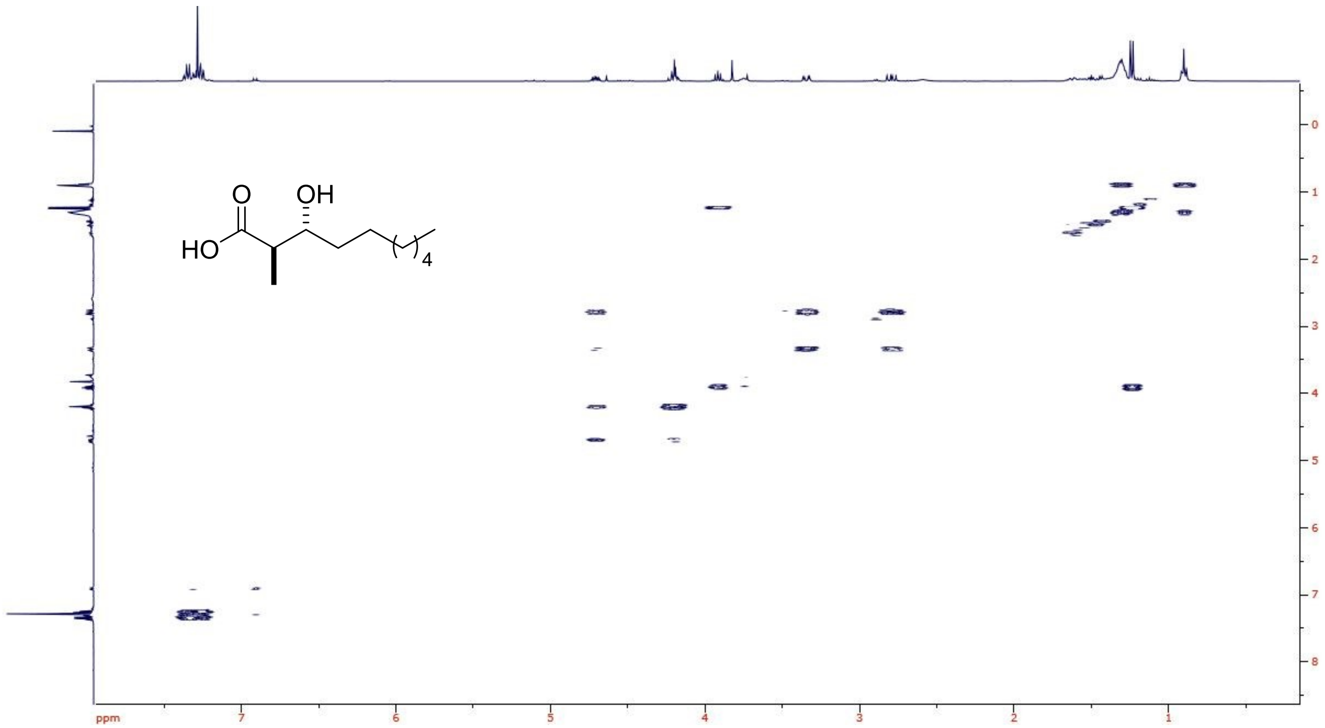

Figure S55: COSY spectrum (CD_3OD) of acid **18**

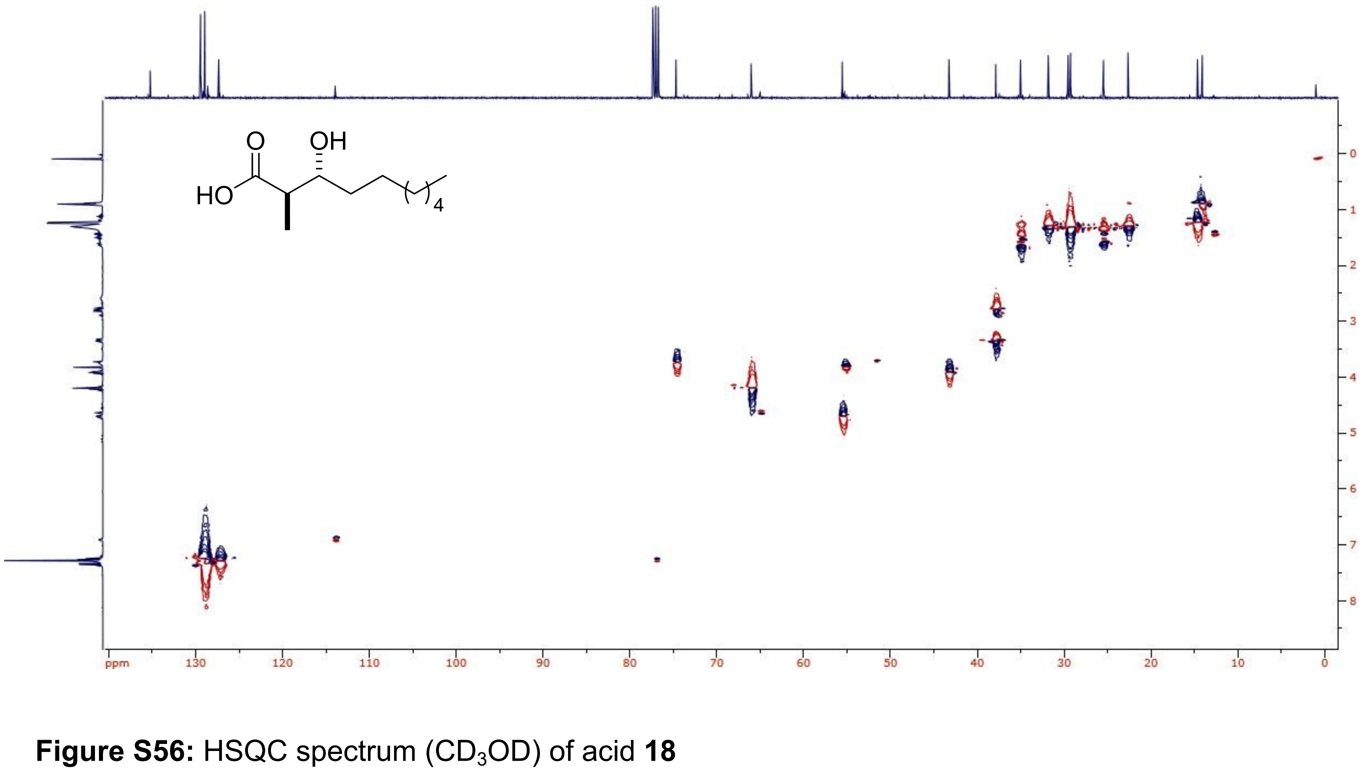

Figure S56: HSQC spectrum (CD_3OD) of acid **18**

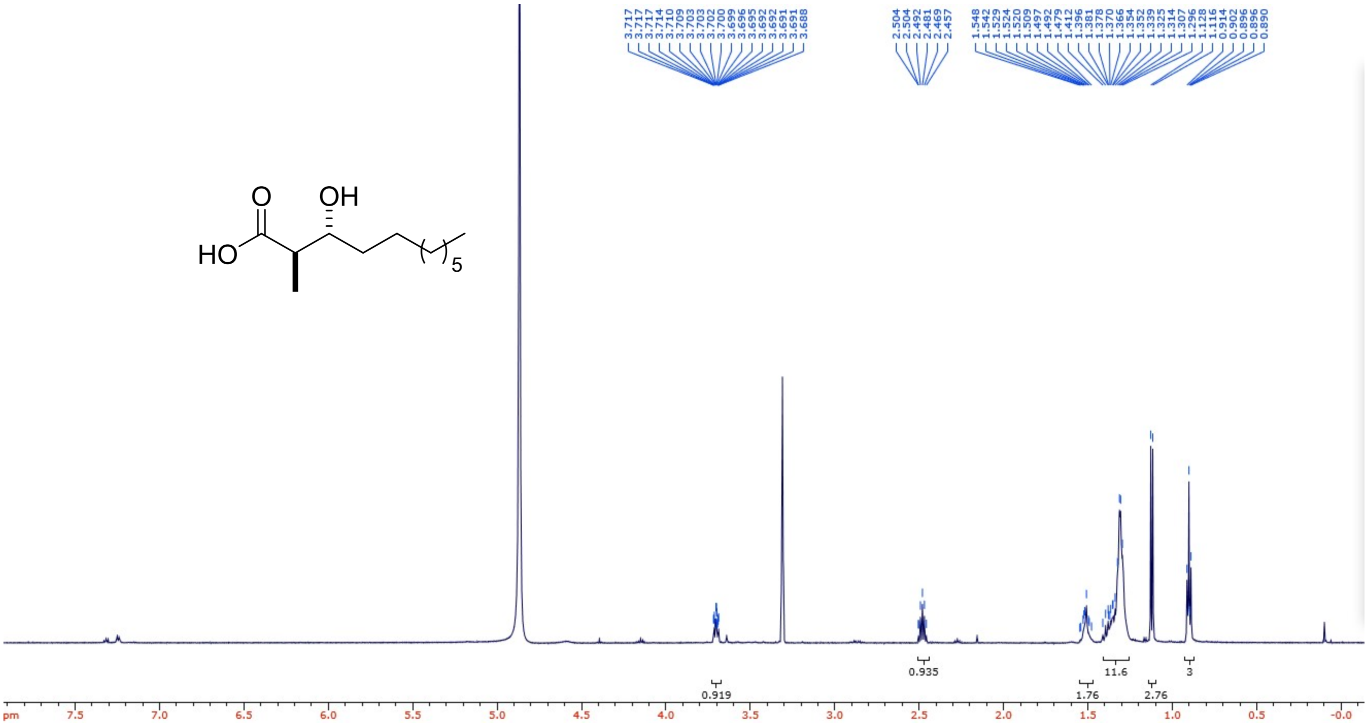

Figure S57: ^{1}H NMR spectrum (600 MHz, CD_3OD) of acid **19**

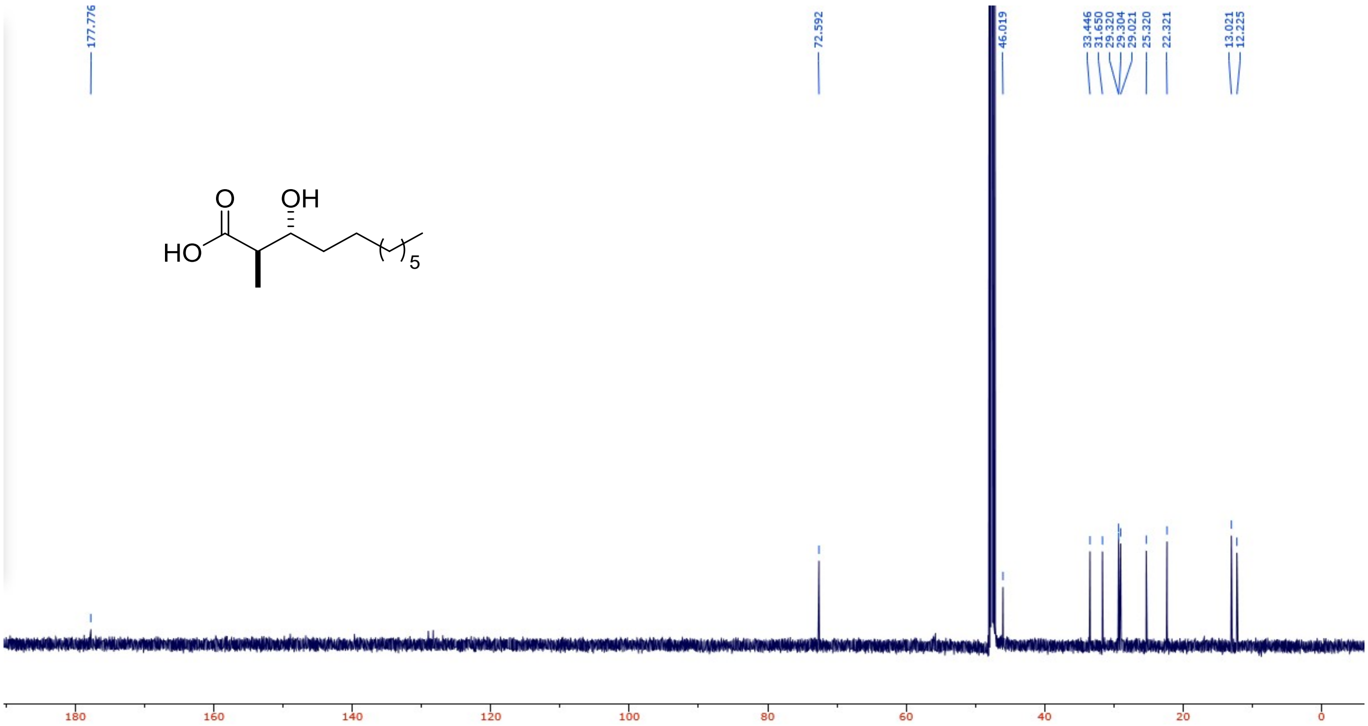

Figure S58: ^{13}C NMR spectrum (151 MHz, CD_3OD) of acid **19**

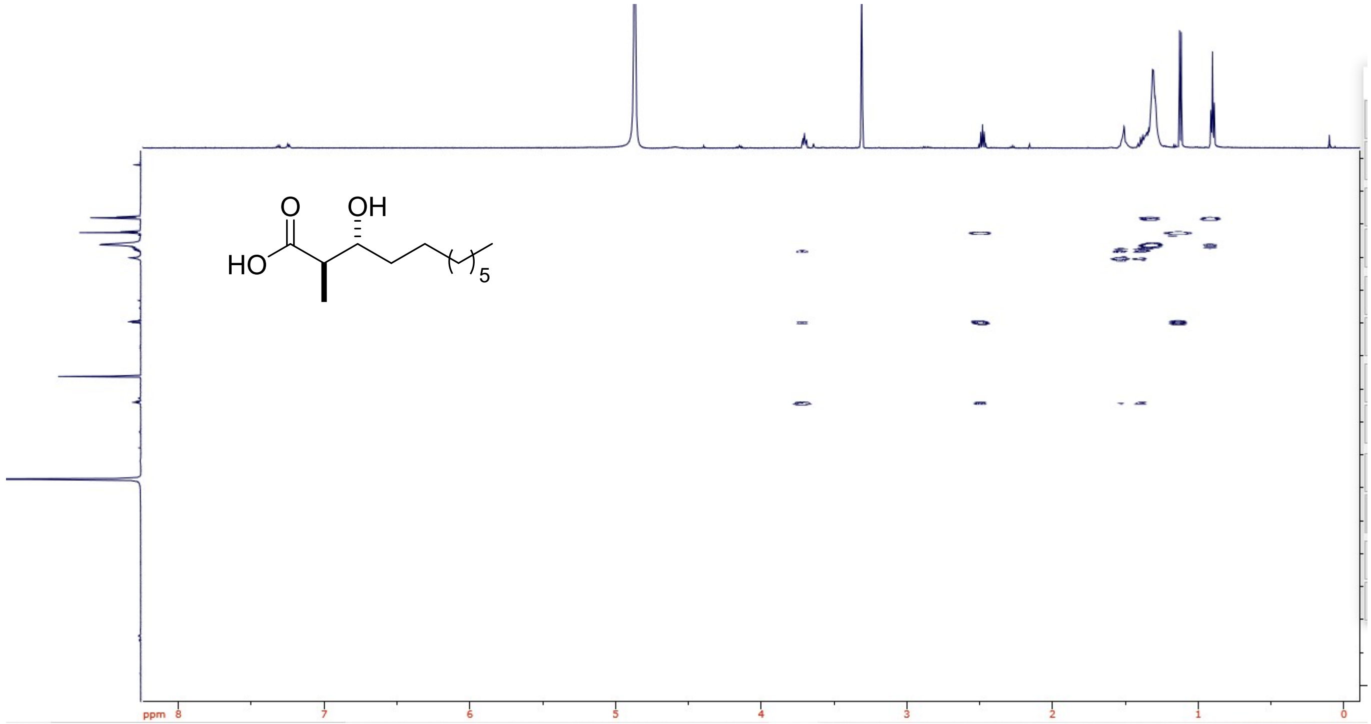

Figure S59: COSY spectrum (CD_3OD) of acid **19**

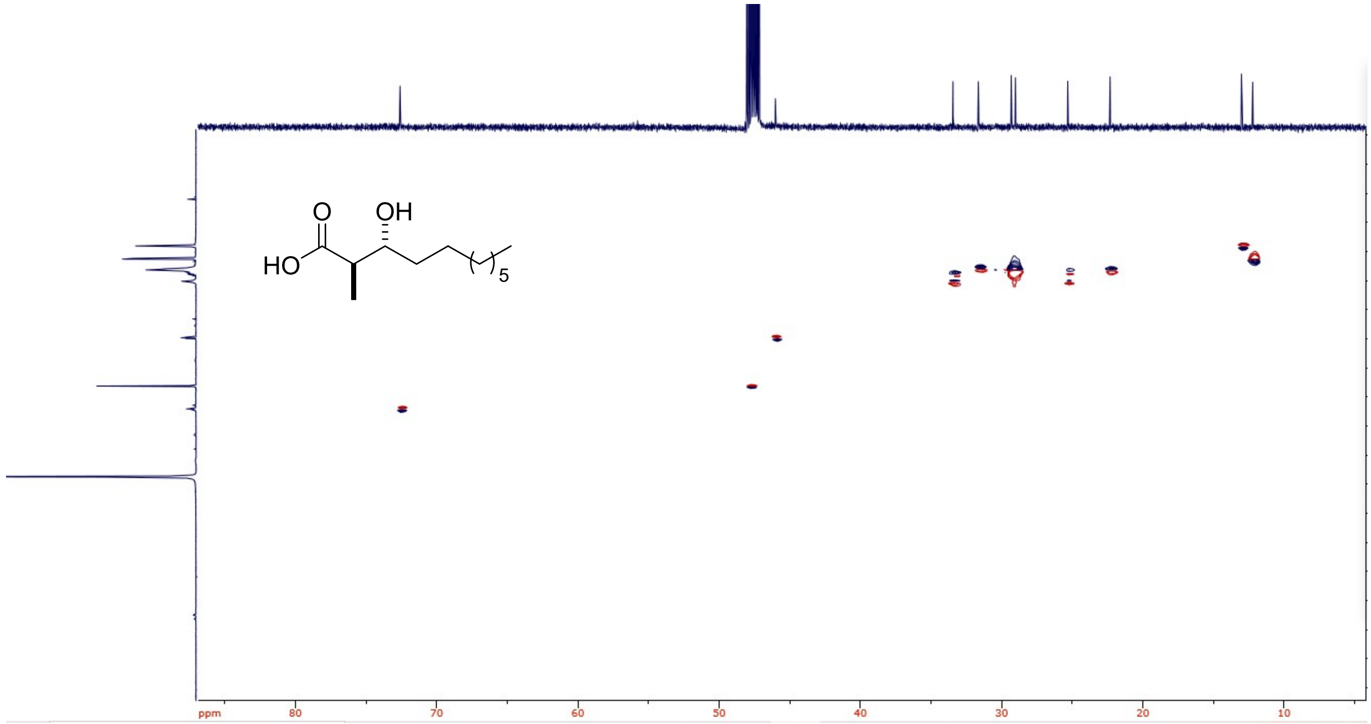

Figure S60: HSQC spectrum (CD_3OD) of acid **19**

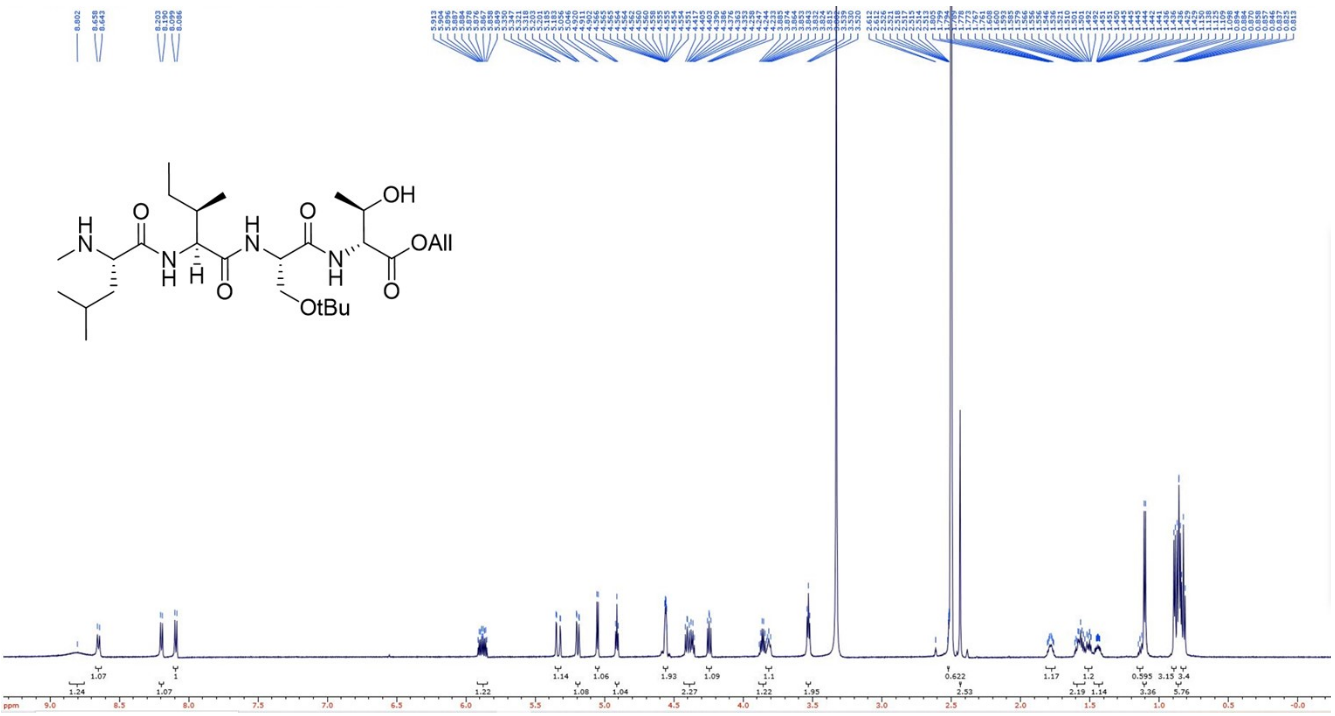

Figure S61: ^{1}H NMR spectrum (600 MHz, DMSO-d_6) of peptide **28**

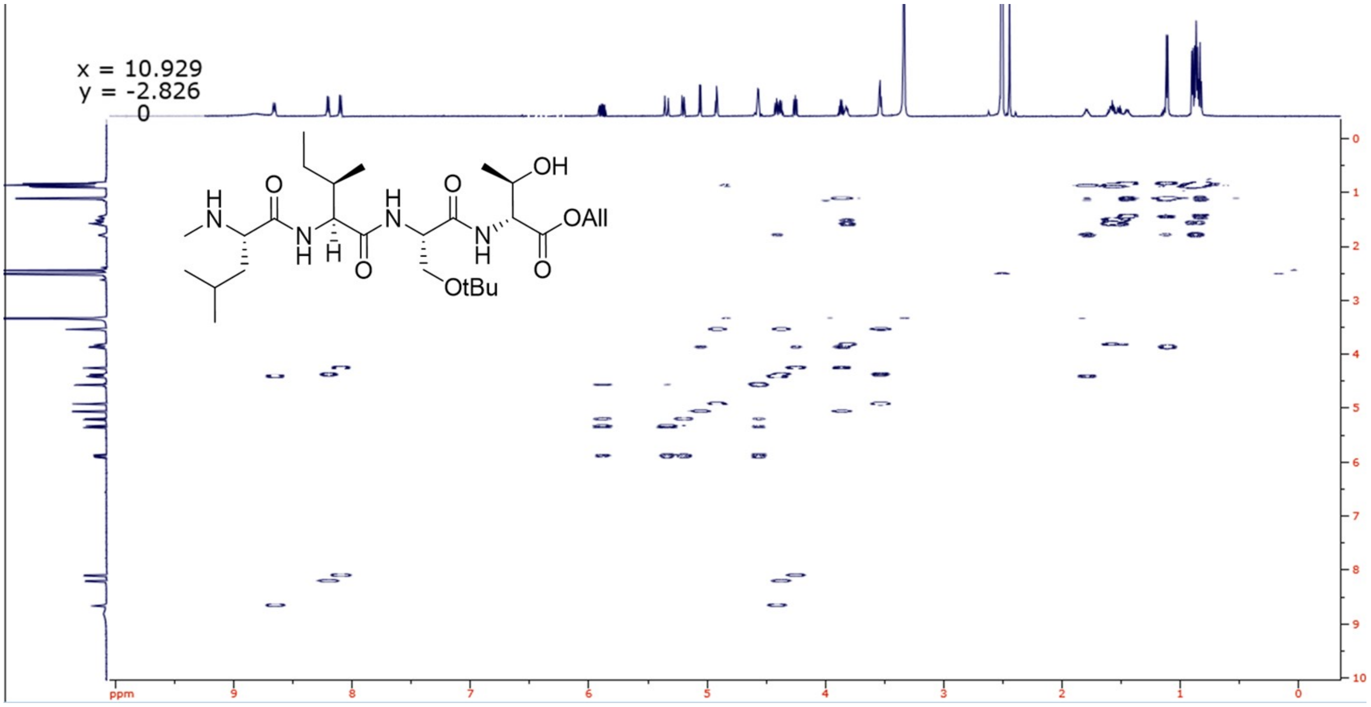

Figure S62: COSY spectrum (600 MHz, DMSO-d_6) of peptide **28**

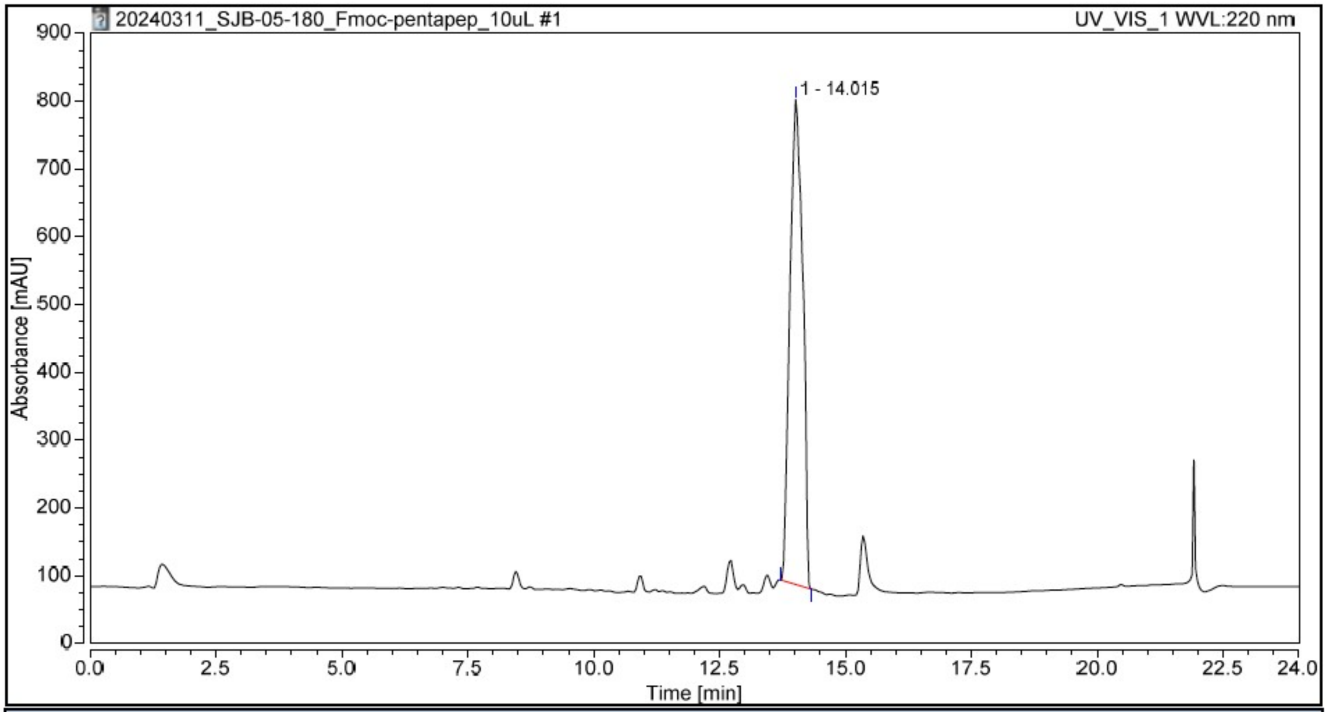

Figure S63: HPLC analysis of microcleavage of pentapeptide **23**

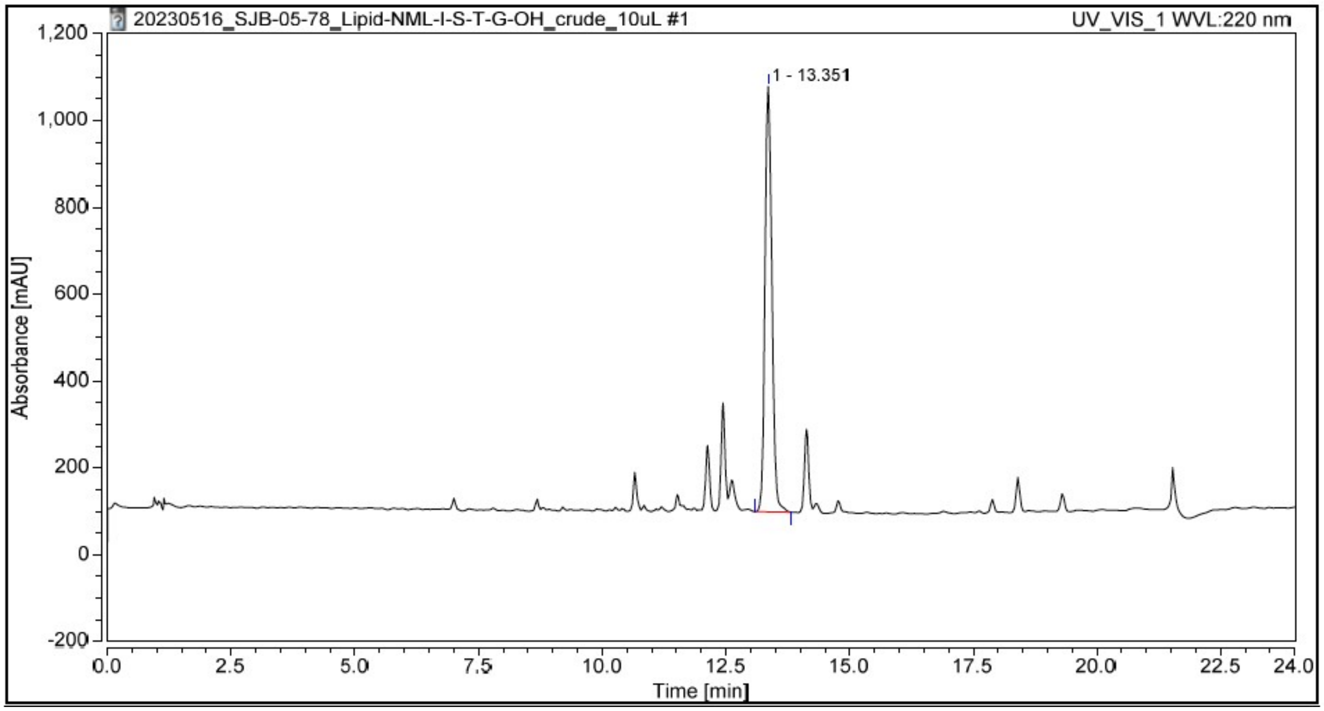

Figure S64: HPLC analysis of microcleavage of acylated pentapeptide **24**

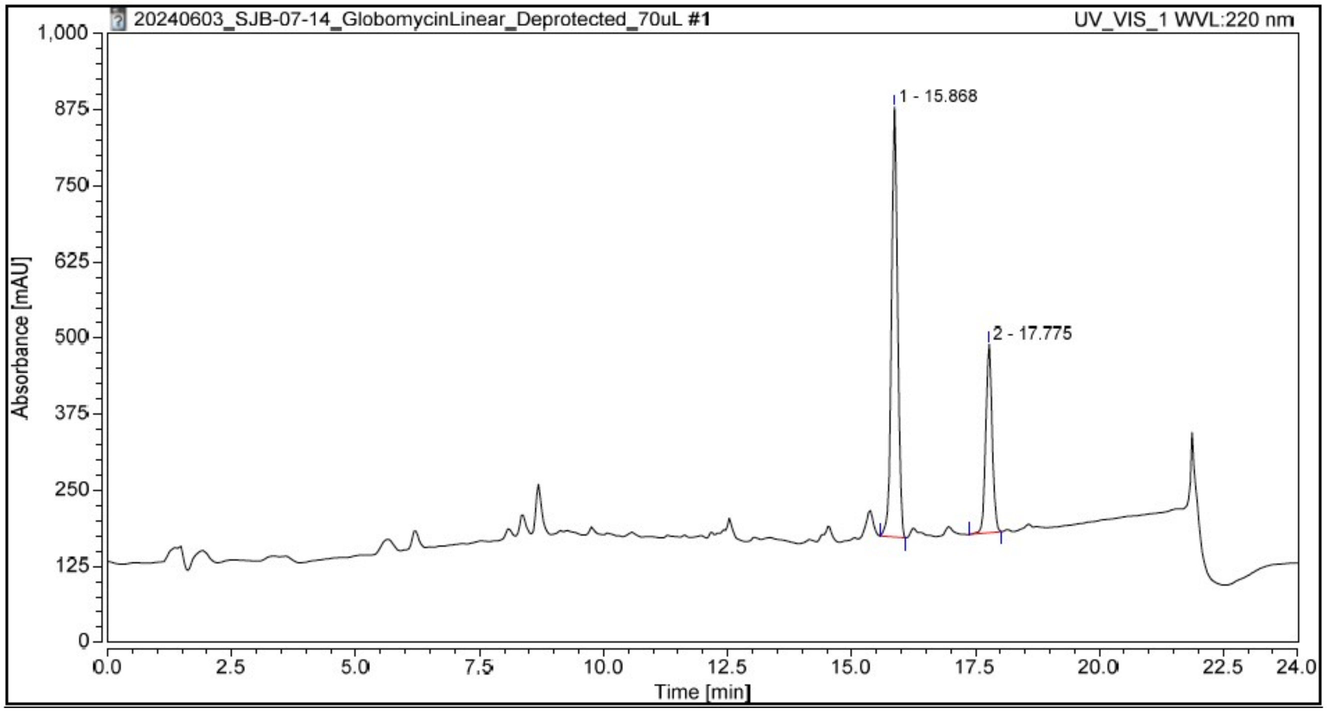

Figure S65: HPLC analysis of microcleavage of peptide **31**
